# Early and dynamic changes in gene expression in septic shock patients: a genome-wide approach

**DOI:** 10.1186/s40635-014-0020-3

**Published:** 2014-08-20

**Authors:** Marie-Angélique Cazalis, Alain Lepape, Fabienne Venet, Florence Frager, Bruno Mougin, Hélène Vallin, Malick Paye, Alexandre Pachot, Guillaume Monneret

**Affiliations:** Joint Unit “Sepsis” Hospices Civils de Lyon - bioMérieux, Hôpital Edouard Herriot, 69437 Lyon, France; Immunology Laboratory, Hôpital Edouard Herriot, 69437 Lyon, France; Intensive Care Units, Centre Hospitalier Lyon-Sud, 69310 Pierre Bénite, France; bioMérieux, Chemin de l’Orme, 69280 Marcy l’Etoile, France; EAM 4174, Université Lyon 1/Hospices Civils de Lyon, 69437 Lyon, France

**Keywords:** Septic shock, Transcriptomic, Microarray, Severity, SAPSII, Genomic response

## Abstract

**Background:**

As early and appropriate care of severe septic patients is associated with better outcome, understanding of the very first events in the disease process is needed. Pan-genomic analyses offer an interesting opportunity to study global genomic response within the very first hours after sepsis.

The objective of this study was to investigate the systemic genomic response in severe intensive care unit (ICU) patients and determine whether patterns of gene expression could be associated with clinical severity evaluated by the severity score.

**Methods:**

Twenty-eight ICU patients were enrolled at the onset of septic shock. Blood samples were collected within 30 min and 24 and 48 h after shock and genomic response was evaluated using microarrays. The genome-wide expression pattern of blood leukocytes was sequentially compared to healthy volunteers and after stratification based on Simplified Acute Physiology Score II (SAPSII) score to identify potential mechanisms of dysregulation.

**Results:**

Septic shock induces a global reprogramming of the whole leukocyte transcriptome affecting multiple functions and pathways (>71% of the whole genome was modified). Most altered pathways were not significantly different between SAPSII-high and SAPSII-low groups of patients. However, the magnitude and the duration of these alterations were different between these two groups. Importantly, we observed that the more severe patients did not exhibit the strongest modulation. This indicates that some regulation mechanisms leading to recovery seem to take place at the early stage.

**Conclusions:**

In conclusion, both pro- and anti-inflammatory processes, measured at the transcriptomic level, are induced within the very first hours after septic shock. Interestingly, the more severe patients did not exhibit the strongest modulation. This highlights that not only the responses mechanisms by themselves but mainly their early and appropriate regulation are crucial for patient recovery. This reinforces the idea that an immediate and tailored aggressive care of patients, aimed at restoring an appropriately regulated immune response, may have a beneficial impact on the outcome.

**Electronic supplementary material:**

The online version of this article (doi:10.1186/s40635-014-0020-3) contains supplementary material, which is available to authorized users.

## Background

Despite improvements in initial resuscitation and supportive care, septic shock remains the major cause of mortality in intensive care units (ICU) worldwide [1]. The failure of 15 years of clinical trials assessing adjunctive therapies together with recent research advances in pathophysiology [2,3] suggest the need to better define the disease and stratify patients allowing for appropriate treatments and targeted therapy. Success in septic shock treatment will rely on a more mediator-directed therapy and thus will require specific and sensitive monitoring tools.

Many studies have aimed at dissecting mechanisms implicated in the disease, and it is now well accepted that injuries in ICU patients lead not only to the initiation of an uncontrolled and exacerbated pro-inflammatory response but also to a deep activation of anti-inflammatory processes [4]. However, the exact chronology of this process remains elusive [5]. It is well accepted that both systemic inflammatory response syndrome (SIRS) and compensatory anti-inflammatory response syndrome (CARS) occur [6,7]. They may be responsible for multiple-organ dysfunction syndrome (MODS) and cell reprograming/immunosuppression [8,9]. From a clinical perspective, several studies have highlighted that the first hours are critical for the response to injury and consequently for the patient’s care and outcome [10–12]. This enforces the importance of characterizing patients early in the process of the disease.

Microarray-based expression profiling provides an interesting opportunity to gain a broader genome level ‘picture’ of a complex and heterogeneous clinical syndromes such as septic shock [13]. These powerful genomic approaches are recommended by opinion leaders to identify specific pathways that could be targeted by different drugs depending on patients’ subset [3]. It is exemplified by a recent genomic description in trauma patients [14], showing that mRNA genes of both immune activation and immune suppression were concomitantly expressed early after injury.

As trauma and septic shock share similarities in pathophysiology and as the understanding of the global host response in sepsis would have a major impact on the successful use of targeted therapies [3], the main objective of the study was to analyze the genome-wide expression patterns of blood leukocytes sequentially during the first 48 h after septic shock. We also investigated whether patterns of gene expression within the first 48 h after septic shock were different in the two extremes of clinical severity using a stratification based on the Simplified Acute Physiology Score II (SAPSII).

## Methods

### Patients

Twenty-eight patients, 18 years and above, admitted to two ICUs of a university hospital for septic shock were included. Their clinical characteristics are shown in Table 1. The diagnosis of septic shock was based on the ACCP/SCCM criteria [15].

**Table 1:** **Patients’ characteristics at admission**

	**SAPSII-low** ***n*** **= 14 (%)**	**SAPSII-high** ***n*** **= 14 (%)**	***p*** **value**	**Total** ***n*** **= 28 (%)**
Female *n* (%)	4 (28.57)	5 (35.72)	ns	9 (32.1)
Male *n* (%)	10 (71.43)	9 (64.28)	ns	19 (67.9)
Age median (Q1-Q3)	59 (46-69)	74 (58-79)	ns	62 (54-76)
Non-survivor in 28 days *n* (%)	1 (7)	4 (28.5)	ns	5 (17.9)
Charlson median (Q1-Q3)	2 (0-2.8)	2.5 (1.3-3.8)	ns	2 (0.75-3.25)
SAPSII on admission median (Q1-Q3)	34 (29-40)	56 (49-63)	<0.0001	45 (34-56)
Duration length in ICU median (Q1-Q3)	10 (5-11)	11 (6-30)	ns	10 (5-14)
SOFA H6	11 (9-13)	10 (9-13)	ns	10 (9-13)
Comorbidity *n* (%)			ns	
0	9 (64.3)	7 (50)		16 (57.1)
1	4 (28.6)	5 (35.7)		9 (32.1)
>2	1 (7.1)	2 (14.3)		3 (10.7)
Type of admission *n* (%)			ns	
Surgery	5 (35.7)	8 (57.1)		13 (46.4)
Medical	9 (64.3)	6 (42.9)		15 (53.6)
Type of infection *n* (%)			ns	
Community acquired	6 (42.86)	9 (64.29)		15 (53.5)
Hospital acquired	8 (57.14)	5 (35.71)		13 (46.4)
Suspected infection *n* (%)				
Clinically documented diagnosis	1 (7)	3 (21.4)		4 (16.6)
Microbiologically documented diagnosis	13 (93)	11 (79)		24 (86)
Bacilli Gram (−)	10 (77)	7 (64)	ns	17 (61)
Cocci Gram (+)	5 (38)	7 (64)	ns	12 (43)
Fungi	2 (15)	0		2 (7)
Cell count				
White blood cells (giga/L)	13.16 (7.52-16.93)	6.97 (3.23-15.68)	ns	11.07 (5.9-16.3)
Lymphocytes	0.73 (0.32-1.22)	0.68 (0.4-1.33)	ns	0.72 (0.35-1.3)
Polymorphonuclear cells	10.14 (6.6-14.31)	5.45 (2.5-12.61)	ns	9.56 (4.21-13.12)
Monocytes	0.57 (0.49-1.66)	0.45 (0.19-0.66)	ns	0.55 (0.38-0.67)

ns, not significant. Significance was designated at the *p* < 0.05 level of confidence. Values represent effectives and percentage indicated in parentheses or median and first and third quartile indicated in parentheses.

Sepsis is defined as the combination of a SIRS and infection diagnosed macroscopically and microbiologically. SIRS is defined as a clinical situation involving at least two of the following clinical criteria: hypothermia (<36°C) or hyperthermia (>38°C), tachycardia (>90/min), tachypnea (>20 breaths/min) and/or arterial PCO_2_ of 32 mmHg or lower and/or mechanical ventilation, and leukocytosis (>12,000/mm^3^) or leukopenia (<4,000/mm^3^).

Septic shock was defined as acute circulatory failure (systolic blood pressure <90 mmHg, mean arterial pressure <65 mmHg, or a reduction in systolic blood pressure >40 mmHg from baseline) despite adequate volume resuscitation. In order to limit potential confounding effects of other conditions affecting immunity, patients with one or more severe comorbidities (i.e., the human immunodeficiency syndrome, hematologic malignancies evolving from insulin-dependent diabetes, the dialyzed chronic renal failure, chronic liver disease stages III and more) or patients receiving immunosuppressive therapy were excluded.

The onset of the septic shock was defined as the beginning of vasopressor therapy. All patients were treated similarly according to the standardized recommendations of our ICU. Severity was assessed using the SAPSII [16]. The first blood sample was collected at the onset of shock (i.e., within 30 min after the beginning of vasoactive treatment: 0, 24, and 48 h after (H0, H24, and H48)). Septic shock patients were divided into SAPSII-low and SAPSII-high groups according to the median of SAPSII score (SAPSII scores of <45 and >45, respectively).

Twenty-five healthy volunteers (median age [Q1-Q3], 48 [40-52] years) with no known comorbidities were also included to provide a panel of control values for mRNA expression.

Protocol was approved by Comité consultatif de Protection de Personnes (CPP) de Lyon A, and informed consent forms were signed by patients (or by a third party based on the patient’s state of consciousness).

### Sample collection, processing, and microarray hybridization

Peripheral blood samples were collected in PAXgene™ Blood RNA tubes (PreAnalytix, Hombrechtikon, Switzerland) in order to stabilize mRNA [17]. Total RNA was extracted according to the manufacturer’s instructions. Briefly, total RNA was isolated using the PAXgene^TM^ blood RNA kit (PreAnalytix). The residual genomic DNA was digested using the RNase-Free DNase set (Qiagen Valencia, CA, USA). The integrity of the total RNA was assessed using Agilent 2100 Bioanalyzer (Agilent Technologies, Santa Clara, CA, USA). The 104 microarray experiments (corresponding to the three time points of the 28 septic shock and the 25 healthy volunteers) were performed as previously described [18]. Briefly, gene expressions were generated using GeneChip® Human Genome U133 Plus 2.0 arrays (Affymetrix, Sta. Clara, CA, USA) according to manufacturer’s protocol. Affymetrix GeneChip Operating Software version 1.4 (GCOS) was used to manage GeneChip array data and to automate the control of GeneChip fluidics stations (FS450) and scanner (GeneChip® Scanner 3000). Data from this experiment have been deposited in the National Center for Biotechnology Information (NCBI) and are available in the GEO DataSets site under accession number GSE57065.

### Reverse transcription and quantitative PCR

Total RNA was reverse transcribed into cDNA using the SuperScript III reverse transcription-polymerase chain reaction (PCR) system (Invitrogen Life Technologies, Carlsbad, CA, USA) according to the manufacturer’s instructions. mRNA expression was quantified using quantitative real-time polymerase chain reaction. Briefly, PCR reactions were performed using a LightCycler instrument with the Fast-Start DNA Master SYBR Green I real-time PCR kit according to the manufacturer’s instructions (Roche, Basel, Switzerland). Thermocycling was performed in a final volume of 20 μL containing 3 mM of MgCl_2_ and 0.5 μM each of the required primers. PCR was performed with an initial denaturation step of 10 min at 95°C followed by 40 cycles of a touchdown PCR protocol (10 s at 95°C, 10 s annealing at 68°C to 58°C, and 16 s extension at 72°C). mRNA expression of genes was investigated using specific cDNA standards. The cDNA standard was prepared from purified PCR amplicons obtained for each candidate genes: TBX21 (FP: 5′-TGTGACCCAGATGATTGTGCT-3′, RP: 5′-AGCTGAGTAATCTCGGCATTC-3′), GATA3 (FP: 5′-AAGCGAAGGCTGTCTGCAGC-3′, RP: 5′-GGGTCTGTTAATATTGTGAAGC-3′), CX3CR1 (FP: 5′-AGTCTGAGCAGGACAGGGTG-3′, RP: 5′-GTCCCAAAGACCACGATGTCC-3′), HLA-DRA (FP: 5′-GCCTCTTCTCAAGCACTGGGA-3′, RP: 5′-CCACCAGACCCACAGTCAGG-3′), IRAK-3 (FP: 5′-CTCGGAATTTCTCTGCCAAG-3′, RP: 5′-GTGGGAGGATCTTCAGCAAA-3′), and for the housekeeping gene peptidylpropyl isomerase B encoding for cyclophilin B; PPIB (FP: 5′-GGAGATGGCACAGGAGGAAAGA-3′, RP: 5′-GGGAGCCGTTGGTGTCTTTG-3′). The second derivative maximum method was used with the LightCycler software to automatically determine the crossing point for individual samples. Standard curves were generated by quadruplicate cDNA standard. Relative standard curves describing the PCR efficiency of selected genes were created and used to perform efficiency-corrected quantification with the LightCycler Relative Quantification Software (Roche Molecular Biochemicals). The results were expressed as a concentration ratio between the target gene mRNA and peptidylpropyl isomerase B mRNA levels.

### Data analysis

Univariate analysis was performed to compare characteristics between groups using either the chi-squared or Mann-Whitney *U* test.

Gene expression data were imported into Partek Genomics Suite 6.5 (Partek, St Louis, MO, USA) as .CEL files using default parameters. Transcriptomic data were normalized with gc-Robust Multi-array Average (gcRMA) algorithm. The RMA method [19] consists of three steps: background adjustment, quantile normalization [20], and probe set summarization of the log-normalized data applying a median polishing procedure. Differential expression analysis was performed using analysis of variance (ANOVA). A step-up false discovery rate (FDR) was applied to *p* values from the linear contrasts to determine a cutoff for significantly differentially expressed genes. Gene lists were created using cutoff of FDR <0.05 and twofold change. Hierarchical clustering was performed using the gene expression module from Partek. Euclidian distance method after normalization by shift mean columns to mean of zero and scale to standard deviation of 1 was used. Gene ontology, functional enrichment, and canonical pathways analyses were performed using Ingenuity Pathway Analysis (IPA) [12,21] (www.ingenuity.com). Fisher’s exact test was used to calculate the *p* value for determining the probability that each function or pathway assigned to the dataset was due to chance alone. The Human Genome U133 Plus 2.0 array was used as the reference.

## Results

### Patients’ clinical characteristics

The patient’s characteristics at admission are presented in Table 1. The age and sex distribution was similar to what is usually observed in septic shock patients’ cohorts, with a percentage of male patients (63.3%) higher than female and a median age of 62 years. The SAPSII and the Sequential Organ Failure Assessment (SOFA) scores were high (median [Q1-Q3]: 45 [34-56] and 11 [9–13] respectively), although the mortality rate was lower (18%) than usually described in the literature (>30%) for such severe patients [22].

### Gene expression patterns in septic shock patients over time

**Figure 1:**
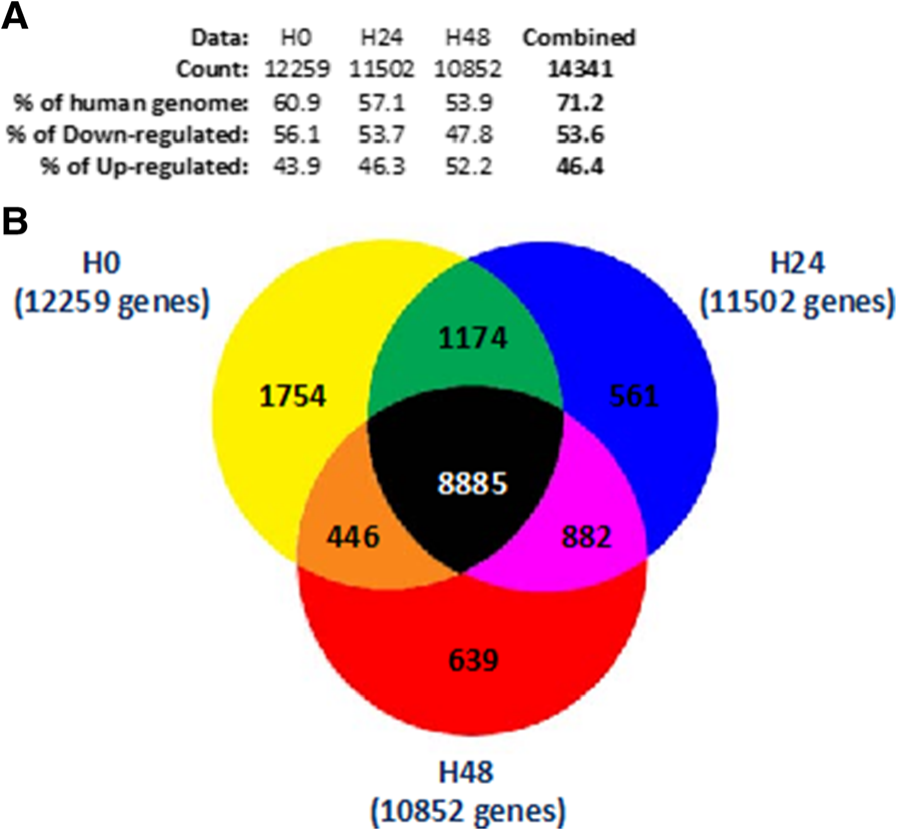
**Genes differentially expressed (GDE) between septic shock patients and healthy volunteers. (A)** Results summary table representing the percentage of modulated genes using a false discovery rate (FDR) adjusted probability <0.05 over the 48 h compared to the human genome (Entrez genes included in HG-U133 plus 2.0 microarray). The table also shows the percentage of significant (FDR <0.05 and FC >2) down- and upregulated genes. **(B)** Venn diagram representing the GDE between septic shock and healthy volunteers using a false discovery rate (FDR) adjusted probability <0.05. Values represent the number of unique Entrez genes found significantly changed in whole blood of patients at the onset of shock (H0) and 24 and 48 h after (H24 and H48).

Our microarray data showed clearly that septic shock patients developed major genomic alterations during the first 48 h after the onset of shock affecting more than 71% of the human genome. As shown in Figure 1A, most of these alterations were already detectable within the first 30 min following admission since more than 60% of the human genome was already altered at H0.

In addition, the transcriptome disruptions observed at H0 appeared to be particularly stable during the study period (Figure 1B). Indeed, over a total of 14,341 unique Entrez genes differentially expressed (FDR adjusted probability <0.05), 8,885 were in common between the three time points (i.e., H0, H24, and H48). While the mRNA expression of most of these genes was already altered at H0, their number seemed to decrease slowly up to H48, indicating that most of the phenomenon had already occurred at admission.

When compared to healthy volunteers, the percentage of downregulated genes at H0 (56.1%) was higher than the percentage of upregulated ones (43.9%). The percentage of downregulated genes trended toward a decrease, while the percentage of upregulated ones increased over the first 48 h (53.7% and 47.8% of the downregulated genes at H24 and H48, respectively).

### Biological functions and pathways involved in septic shock response

The gene ontology analysis performed on 8,885 genes commonly modulated at the three time points showed an enrichment of infectious disease processes (Table 2). In addition, most of the molecular functions deregulated during the first 48 h were associated with cellular rearrangement.

**Table 2:** **Top biological functions enriched by genomic variations in septic shock patients**

**Name**	***p*** **value**	**Number of genes**	**Percentage in tGDE**	**Percentage in function**
Diseases and disorders				
Infectious disease	2.70E−15 to 3.60E−03	1,097	12.3	32.5
Respiratory disease	9.97E−12 to 4.57E−03	194	2.2	5.7
Organismal injury and abnormalities	2.56E−10 to 5.01E−03	316	3.6	8.6
Dermatological diseases and conditions	5.33E−10 to 3.80E−03	295	3.3	9.9
Renal and urological disease	7.73E−10 to 7.73E−10	206	2.3	8.7
Molecular and cellular functions				
Cellular development	3.15E−14 to 5.88E−03	933	10.5	15.6
RNA post-transcriptional modification	4.22E−14 to 8.93E−04	196	2.2	45.1
Cell death	3.53E−13 to 6.16E−03	1,880	21.2	30.7
Cellular function and maintenance	8.28E−12 to 5.94E−03	739	8.3	13.4
Cell morphology	1.16E−09 to 4.80E−03	400	4.5	8.9
Physiological system development and function				
Hematological system development and function	3.88E−15 to 6.17E−03	1,158	13.0	29.7
Hematopoiesis	3.88E−15 to 4.66E−03	681	7.7	38.4
Lymphoid tissue structure and development	5.06E−11 to 5.10E−03	528	5.9	35.4
Cell-mediated immune response	2.16E−10 to 1.80E−03	305	3.4	33.0
Tissue morphology	2.52E−10 to 4.84E−03	628	7.1	14.0

Results are obtained from the 8,885 common genes differentially expressed between septic shock patients and healthy volunteers over time using false discovery rate <0.05. The ‘*p* value’ column represents the range of significance for the specific functions included in the high level categories described. The *p* value is a measure of the likelihood that the association between a set of genes in the dataset and a related function is due to random association. *p* values <0.05 indicate a statistically significant, non-random association. The *p* value is calculated by the Fisher’s exact test. The ‘percentage in tGDE’ column represents the percentage of genes found in a function compared to the total number of gene differentially expressed (*n* = 8,885). The ‘percentage in function’ column represents the percentage of genes found in a function compared to the total number of genes described by IPA to be associated with the function.

**Figure 2:**
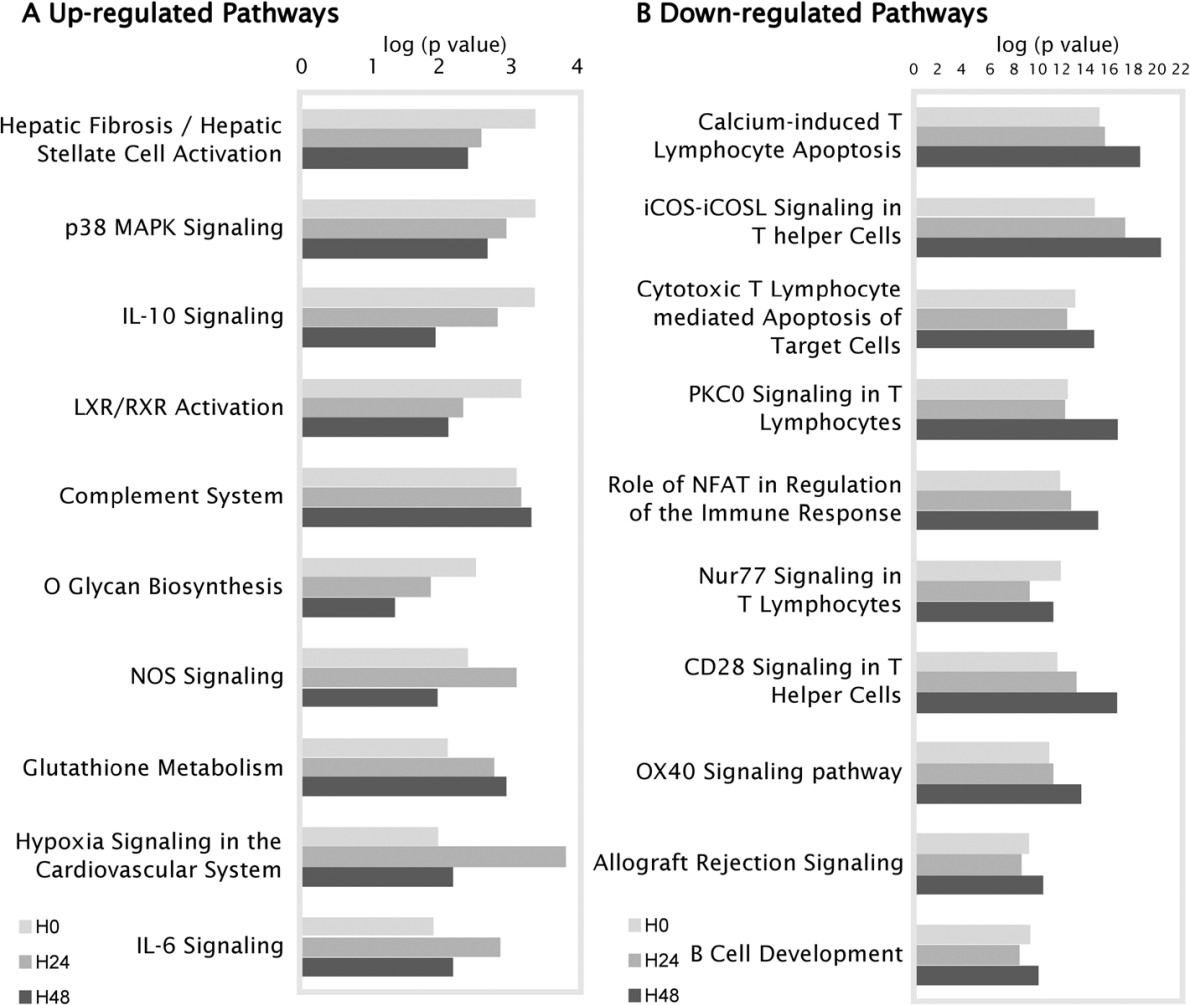
**Top canonical pathways affected in septic shock.** **(A and** **B)** The 10 top up- and downregulated canonical pathways enriched by genes differentially expressed over time. The graph shows the −log10 (*p* value) of the enrichment of the pathway.

Among the most increased canonical pathways enriched at H0 (Figure 2A), 6 out of 10 were related to molecules involved in the endotoxin tolerance or pathogen recognition as well as cytokines/cytokine receptors, leading to increased inflammation and innate immune response. For example, the expressions of IL1R1, IL1R2, IL1RAP, IL1RN, IL18, IL18RAP, IL4R, IL10, IFNGR1, TGFBR1, IRAK3, MAP2K6, MAPK1, MAPK14, SOCS3, S100A8, MMP9, LY96, JAK2, JAK3, and NFKBIA were significantly increased after shock.

In parallel, most of the top 10 gene families suppressed after septic shock were related to T cell signaling and antigen-mediated response (Figure 2B). For example, expressions of gene part of the T cell receptor (TCR) complex such as CD247, CD3E, CD3G, and CD3D were markedly decreased, as well as antigen-presentation genes like HLA-DMA, HLA-DOA, HLA-DRA, HLA-DQA1, CIITA, HLA-DRB1, HLA-DOB, HLA-DMB, CD74, HLA-DPB1, TAP2, and HLA-DPA1. Importantly, the transcriptional modulation observed was very consistent over the time period as illustrated by the fold change of these selected genes (Additional file 1: Table S1).

**Figure 3:**
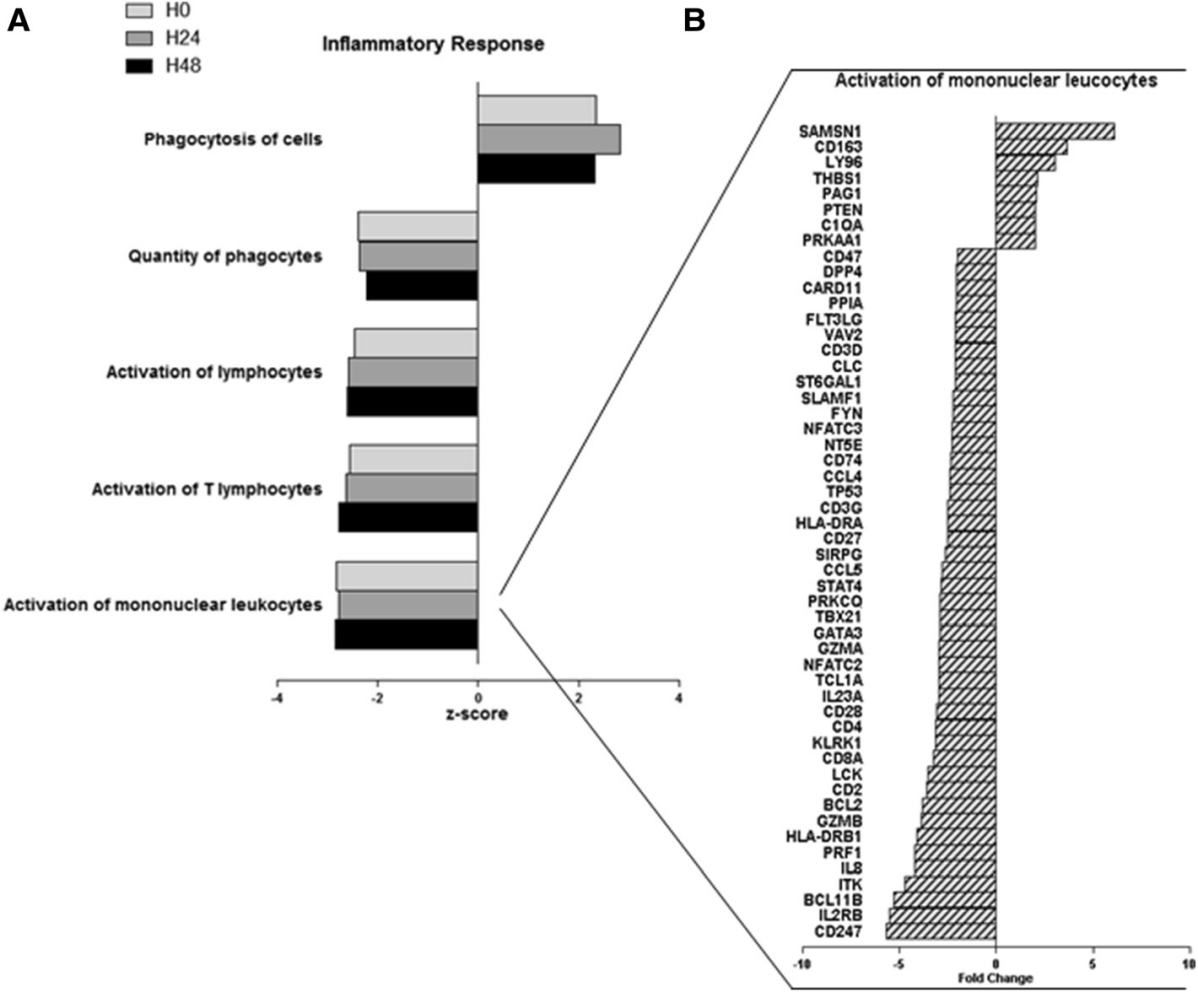
**Enriched biological category analysis illustrated by inflammatory response function. (A)** Inflammatory response functions (high-level functional category) found to be enriched by genes differentially expressed between septic shock patients and healthy volunteers over the 48 h. The *z*-score value predicts the direction of change for the function. An absolute *z*-score of ≥2 is considered significant. A function is predicted to be increased if the *z*-score is ≥2 and decreased if the *z*-score is less than or equal to −2. Data for the comparison of septic shock patients versus healthy volunteers are represented at the onset of shock (H0) and 24 and 48 h after (H24, H48). **(B)** Highlight on the 52 genes that have expression direction consistent with decreases in activation of mononuclear leukocytes process.

Similarly, we looked at the global host response with the predicted activation or inhibition status of these enriched functions (Additional file 2: Table S2). The inflammatory response process was the most enriched category over time, including functions predicted to be inhibited as well as activated (Figure 3A). For example, 52 genes included in the function ‘activation of mononuclear leukocytes’ showed expression direction consistent with the decrease of the function, therefore suggesting an anergy of those cells. In particular, the gene whose expression increased the most was SAMSN1 (fold change 9.04) (Figure 3B). Inversely, the gene whose expression decreased the most was CD247 (i.e., CD3 zeta chain, fold change −5.19). In details, among these 52 genes included in the function activation of mononuclear leukocytes, several could be directly linked to the T cell receptor signaling pathway (CD247, CD28, CD3D, CD3G, CD4, CD8A, FYN, ITK, LCK, NFATC2, NFATC3, PAG1, PRKCQ, and VAV2) or to some activation pathway like glucocorticoid receptor signaling (BCL2, CCL5, CD163, CD247, CD3D, CD3G, IL8, NFATC2, NFATC3, and PRKAA1) or p53 signaling (BCL2, PTEN, THBS1, and TP53). Most of them were transmembrane receptors like CD molecules (CD2, CD3D, CD3G, CD4, CD8A, CD27, CD28, CD47, CD163, and CD247), MHC genes (CD74, HLA-DRA, and HLA-DRB1), or transcription factors (GATA3, NFATC2, NFATC3, STAT4, TBX21, and TP53).

Inversely, the function ‘phagocytosis of cells’ was predicted to be activated during the kinetic. The 22 genes involved in this function were found with expressions consistent with its activation, either increased (e.g., PROS1, MERTK, FCGR1A, THBS1, or ITGAM), or decreased (e.g., CD47, FOXP1, or FYN).

Five genes selected within these functional categories were validated by qRT-PCR. The gene expressions measured by either microarray or qRT-PCR were highly correlated (Additional file 3: Figure S1).

### Functional analysis of genes in severe versus less severe patients

SAPSII is a reliable score for severity assessment in septic patients and is therefore frequently used to stratify patients in clinical studies [23]. In order to investigate whether the two extremes of severity after septic shock could be associated with different genomic responses and overtime evolutions, we used the median SAPSII values to stratify patients in our cohort in severe (SAPSII-high group, SAPSII >45) and less severe (SAPSII-low group, SAPSII <45) groups. The clinical characteristics for the SAPSII-high and SAPSII-low groups are presented in Table 1. A difference, although not significant, was observed in mortality rate between the two severity groups. We also observed a small difference between cell counts at admission, with decreased number of white blood cells in SAPSII-high group at admission (6.97 versus 13.16 in SAPSII-low group). This difference tended to decrease over the time period, with 13.98 and 11.47 giga/L white blood cells at H48 for SAPSII-low and SAPSII-high groups, respectively (data not shown).

**Figure 4:**
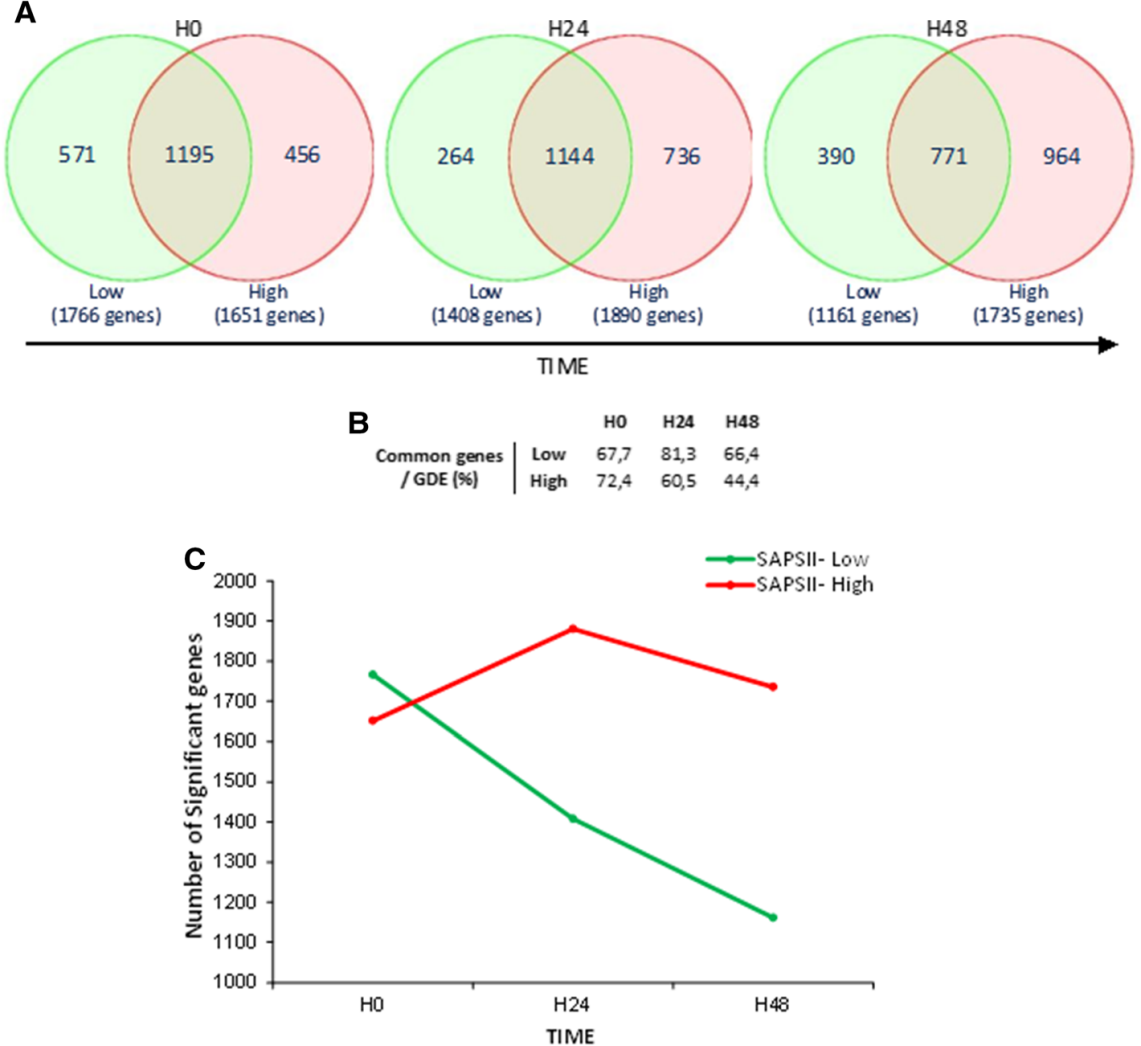
**GDE between SAPSII-high and SAPSII-low patients versus healthy volunteers. (A)** Venn diagram representing the GDE using FDR-adjusted probability <0.05. Values represent the number of unique Entrez genes found significantly changed in whole blood of SAPSII-high and SAPSII-low groups at the onset of shock (H0) and 24 and 48 h after (H24 and H48) compared to healthy volunteers. **(B)** Results summary table representing the percentage of common genes in both groups over the 48 h. **(C)** Number of SAPSII-low and SAPSII-high significant upregulated genes (FDR <0.05, fold change >2) compared to healthy volunteers at H0, H24, and H48.

Interestingly, an equal number of genes was significantly modulated (FDR <0.05; fold change >2) in SAPSII-high and SAPSII-low groups compared to healthy volunteers at the onset of shock (more than 67% of genes differentially expressed were in common - Figure 4B). The highest difference in the response between SAPSII-low and SAPSII-high groups was observed at H48 (1,735 genes differentially expressed in SAPSII-high analysis compared to 1,161 in SAPSII-low group leading to only 44% of commonly regulated genes at this time point) (Figure 4). Although the two severity groups seemed to have the same genomic response at H0, the number of altered genes in SAPSII-low patients decreased over the 48 h study period, returning to control values, whereas it remained stable in the SAPSII-high group even with a trend toward increase (Figure 4C).

**Figure 5:**
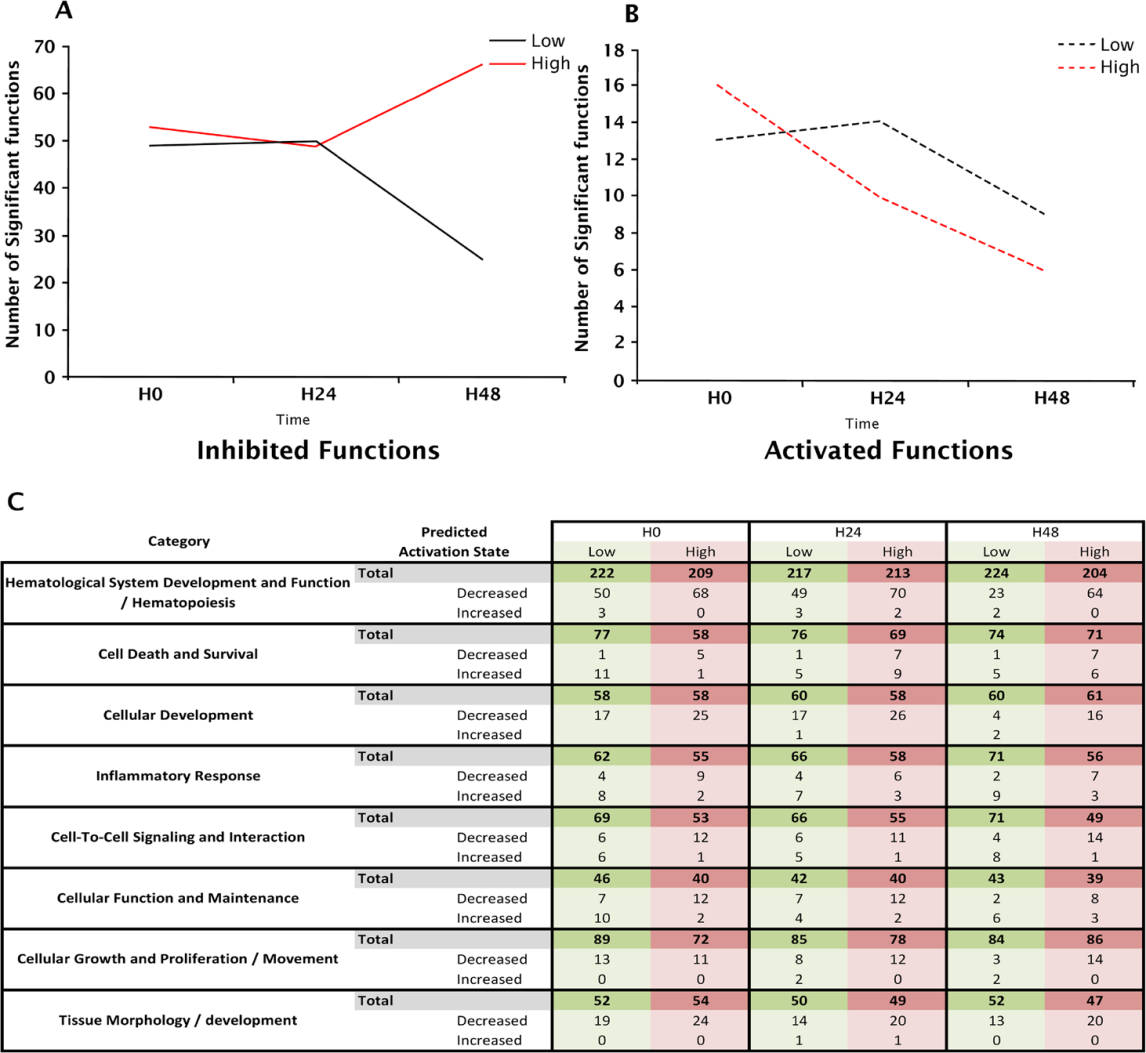
**Enriched functions of SAPSII-low and SAPSII-high patients compared to healthy volunteers.** **(A and** **B)** The number of SAPSII-high and SAPSII-low significantly decreased and increased functions (absolute *z*-score ≥2) compared to healthy volunteers at H0, H24, and H48. **(C)** Table of principal enriched functions in SAPSII-low and SAPSII-high groups compared to healthy volunteers (HV) at the onset of shock 0, 24, and 48 h after. For each category, the total number and the number of enriched functions predicted to be activated or inhibited are indicated.

A functional analysis showed that the number of significantly enriched inhibited functions was much higher than the number of activated ones (≈50 versus 15) (Figure 5A,B). In the SAPSII-low patients group, a steady decrease in the number of statistically enriched functions was observed (both inhibited and activated), while the number of activated functions remained quite stable in the SAPSII-high patients group (from 13 at H0 to 10 at H48) and the number of inhibited functions increased over time (50 at H0 to 70 at H48).

The same functions were enriched in both groups reflecting that the overall changes (direction of the responses) in gene expression at H0 between the two severity groups was very similar (Figure 5C). For example, cellular and metabolic functions (e.g., cellular development or hematological systems) were found significantly enriched and mostly inhibited in both groups. Conversely, some differences were observed in the predicted activation state of functions. For example at H0, in the ‘cell death and viability’ category, a number of functions were predicted to be activated in SAPSII-low group whereas these were predicted to be inhibited in SAPSII-high group. Most of the activated functions were related to cell death and apoptosis, trending toward a decrease during the kinetic in SAPSII-low group. Therefore, the predicted *z*-score for cell death of mononuclear leukocytes decreased from 2.9 to 1 in SAPSII-low group whereas it increased from 1.7 to 2.4 in SAPSII-high group (Additional file 4: Table S3). In this function, PTEN gene, whose main function consists in the induction of apoptosis via caspase activation, was overexpressed in the SAPSII-low group at H0 (FC = 2.26 and 1.52 compared to healthy volunteers for SAPSII-low and SAPSII-high, respectively). In agreement, the expression of pro-apoptotic CASP1 and CASP4 was more increased in SAPSII-low group (FC = 2.15 and 2.07 compared to SAPSII-high FC = 1.55 and 1.14, respectively).

The inhibiting functions exhibited by the SAPSII-high patient group were mostly related to cytotoxicity of cells, cytolysis, and viability functions. Thus, the predicted *z*-scores of ‘cytotoxicity of lymphocytes’ functions were −2.9, −2.5, and −3.2 at H0, H24, and H48, respectively.

### Genes differentially expressed between severe and less severe patients

**Figure 6:**
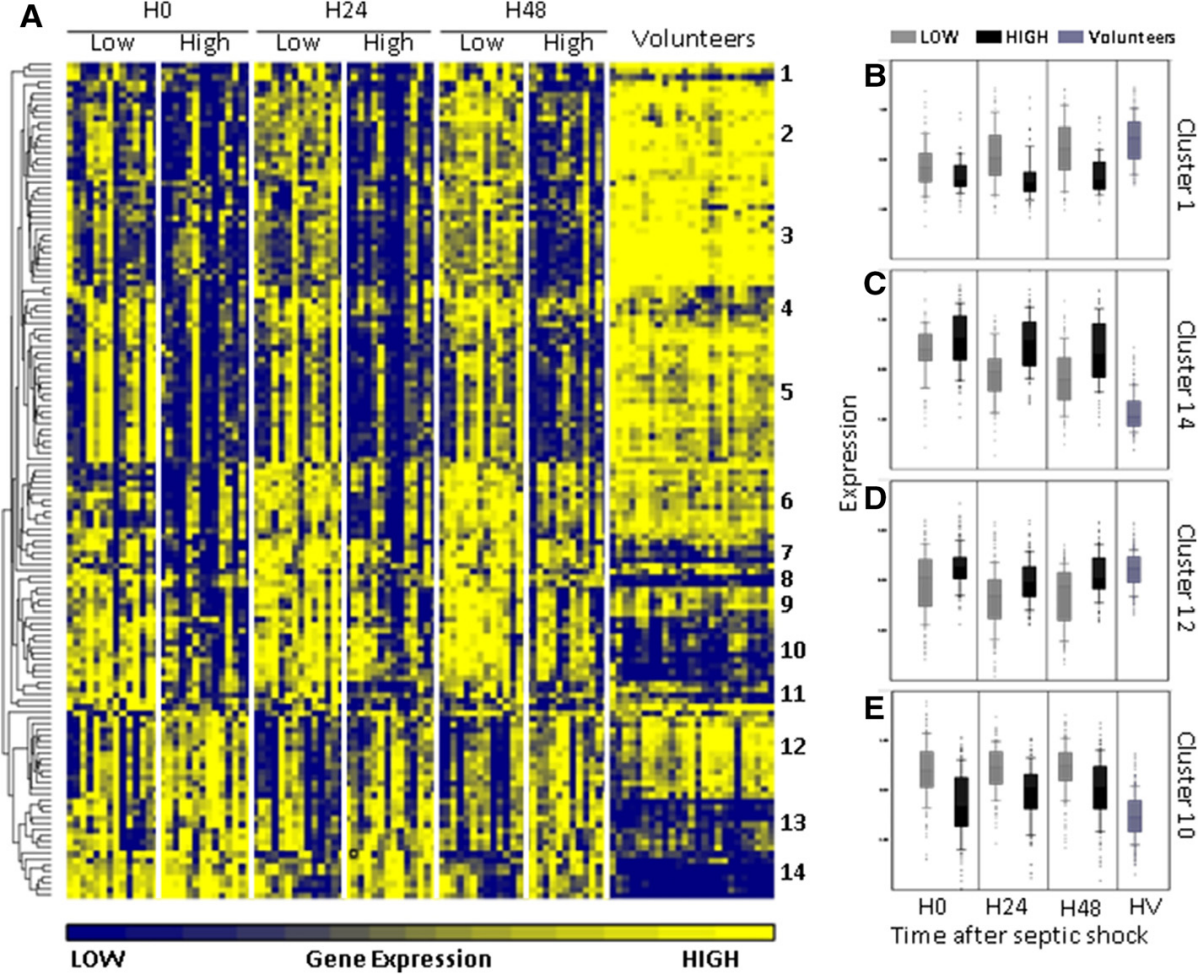
**Differences in gene expression between SAPSII-low and SAPSII-high groups. (A)** Heat map of 142 probe sets whose expression was (i) greater than or equal to twofold different (FDR <0.05) when comparing healthy volunteers with SAPSII-low (Low) or SAPSII-high (High) groups and (ii) also significantly different (FDR <0.05) between both groups of septic shock patients. Clustering was done using Euclidian distance method after normalization (shift probe set expression to mean of zero and scale to standard deviation of 1). **(B)** A cluster illustrating genes that are downmodulated in patients versus controls, with genes remaining downmodulated over time in SAPSII-high group only. **(C)** A cluster illustrating genes that are upmodulated in patients versus controls, with genes remaining upmodulated over time in SAPSII-high group only. **(D)** A cluster illustrating a downmodulation in SAPSII-low group only. **(E)** A cluster illustrating an upmodulation in SAPSII-low group only.

Among the genes differentially expressed between septic shock patients and healthy volunteers (twofold difference - FDR <0.05), only 142 probe sets (corresponding to 122 unique Entrez gene) allowed to significantly distinguish SAPSII-low and SAPSII-high groups of patients (FDR <0.05). Figure 6 represents a heat map of these 142 probe sets.

Most of the genes that were differentially expressed between the two groups were implicated in inflammatory and infection responses (Additional file 5: Table S4). The trend of the response was similar in both groups, and differences were observed only at the level and the duration of the genomic response between the two severity groups.

These 142 genes were hierarchically arranged into 14 clusters (Additional file 6: Figure S2 and Figure 6). Those 14 clusters followed four distinct patterns of expressions illustrated by the 4 clusters displayed in Figure 6 B,C,D,E.

For example, clusters 1 to 6 (Figure 6B) consisted in a panel of genes whose expressions were less decreased and recovered rapidly to normal value during the 48 h in SAPSII-low group but were decreased in SAPSII-high group with a delayed recovery. The genes following this trend of expression were related to leukocytes activation and immune response (BCL11B, BTN3A1, BTN3A2, BTN3A3, CX3CR1, DDX58, F2RL1, HLA-DQA1, HLA-DQB1, HLA-DPB1, IGHM, IGKC, IL7R, IRF1, LCK, LTB, LY75, MME, MMP12, PDE4B, PECAM1, PI3, PRKCH, PSMB9, RASGRP1, RORA, SFN, STAT1, and ZFP36L2) and mostly linked to cell death (APOL6, AQP3, BCL11B, CAMK1D, CX3CR1, DDX58, F2RL1, FGL2, GIMAP4, GZMA, IGHM, IGKC, IL32, IL7R, IRF1, ITGA4, LCK, LEF1, LTB, MME, MX1, PARP14, PDE4B, PECAM1, PI3, POLB, PRKCH, PTGER4, RASGRP1, SEMA4D, SGK1, STAT1, TAP2, XAF1, and ZFP36L2).

Clusters 13 and 14 (Figure 6C) illustrated genes with a small increase in gene expression and a rapid recovery in SAPSII-low group of patients and a high increase over time in SAPSII-high patients. Among these, we observed genes related to infection and cell viability (ALOX12, ANKRD9, CA2, LCN2, MS4A4A, OLFM4, PF4, PPBP, RETN, and TCN1).

The last two patterns of expressions are illustrated by clusters 7 to 11 and 12 (Figure 6D,E). In contrast, to the previous patterns (clusters 1 to 6 and 13 and 14), they showed an earlier and more pronounced response (over- and under-expressed) in the SAPSII-low group. Genes found in these clusters were mostly implicated in bacterial or viral infections (AGTRAP, CARD16, CASP1, CD274, CD46, CFD, CLEC2B, DDX60L, HSP90AB1, MAN1A1, PKN2, and SLPI) and cell death or viability (AGTRAP, BAG1, CASP1, CD274, CD46, CFD, HSP90AB1, HSPB1, LRRK2, PKN2, RBM3, SLAMF7, SLC6A8, SLPI, and SNCA).

## Discussion

To our knowledge, this is the first study describing the very early genomic response to septic shock. This study provides an important overview of the genome-wide expression patterns of blood leukocytes over three time points (within 30 min after diagnosis and 24 and 48 h afterwards). Septic shock generates a massive genomic modulation with more than 71% of the host transcriptome altered during the first 48 h after shock. This phenomenon is a very aggressive process as it occurs rapidly and is already present at the very early stage of the syndrome with more than 60% of the modification already in place (Figure 1). As described in trauma patients [14], septic shock appears to produce a global reprogramming of the leukocyte transcriptome affecting multiple cellular and molecular functions and pathways. However, the magnitude of the transcriptome modifications seemed to be earlier, faster, and bigger in septic shock, as regards of the extent and rapidity by which the deregulation occurred. Indeed, more than 71% of the genomic modifications occurred during the first 48 h, whereas most of the alterations described by Xiao et al. in trauma patients were observed over the first 28 days.

Most of the altered molecular functions were associated to cellular rearrangement (morphology, function, maintenance, and development), therefore suggesting metabolic adaptations (Table 2). Indeed, several studies have shown that metabolic modifications occur during severe injuries and could lead to a metabolic hibernation. This phenomenon plays a role in protecting cells from death leading to multiple organ failures [24,25] and immune-inflammation control [26].

The decrease of leukocyte pathways and functions (Figures 2B and 5A) suggests an anergy of those cells. As an example, SAMSN1, whose expression increased the most (Figure 3B), has been defined as an inhibitor of B cell spreading [27]. Inversely, CD247, whose expression decreased the most, is the CD3ζ-chain, considered as the rate-limiting factor of TCR/CD3 complex formation [28]. This was consistent with the decreased expression of CD3 on circulating lymphocytes observed in septic shock patients [29]. Inversely, the function ‘phagocytosis of cells’ was predicted to be activated during the kinetic. These results could be related to efferocytosis of dying/dead cells triggered by anti-inflammatory signals in a process of resolving inflammation [30].

Taken together, the patterns of gene expression highlighted that the mechanisms of immune activation and repression are obviously concomitant at the very early phase of septic shock (Figure 3 and Additional file 6: Figure S2), probably before obvious clinical signs. We cannot exclude that earlier modification in gene expression might also have occurred before patients fulfilled the diagnostic criteria of septic shock. Here, we described only the modulation that were observed during the first 48 h after beginning vasopressor therapy. This is in accordance with numerous clinical observations made in the literature showing altered leukocyte functions after septic shock [4,31–33]. This also agrees with previously published data regarding transcription profiles in human sepsis [34] and in trauma patients [14].

Of utmost importance, the early regulation mechanisms observed suggests that the time window to improve the outcome of patient with appropriate therapeutic begins very early after the onset of shock. This observation has been also suggested recently [35].

Interestingly, when comparing genomic responses of the two severity groups, we observed that the overall changes (direction of the responses) in gene expression at H0 between these groups were very similar. Importantly, the difference between the two severity groups was particularly noticeable in the degree and the duration of the altered acute inflammatory response.

Most of the activated functions in both groups were related to cell death and apoptosis. However, they were predicted to be activated and trending toward a decrease during the kinetic in SAPSII-low group, whereas these were predicted to be inhibited in SAPSII-high group. These modifications could be related to T cells undergoing apoptosis, therefore limiting self-harmful effects for the host [36]. This observation emphasized that an appropriate response very early in the process might limit or avoid exacerbated inflammation that would lead to increase severity. Inhibited cytotoxicity of cells and ‘viability’ functions, observed in SAPSII-high patient group, again suggests that the SAPSII-low patient group may have developed a better response (or an appropriate response regulation) and a rapid trend toward recovery, while the SAPSII-high group of patients showed a delayed response and an impaired immune dysfunction.

Four distinct patterns associated with the two groups of severity were observed. The two first patterns indicated that SAPSII-low group seemed to have smaller and shorter deregulation of their genomic response as compared with the more severe patients. For example, in clusters 13 and 14 (Figure 6C), OLFM4 (negative regulator of bacterial killing) and RETN and MS4A4A (related to PPARγ that overexpression has been linked to poor outcome [37]) had increased expressions and no return to baseline in SAPSII-high group. This suggested that the bacterial clearance and cell viability were more jeopardized in this group.

Once again, these first two patterns indicated that SAPSII-low group seemed to have smaller and shorter deregulation of their genomic response as compared with the more severe patients.

In contrast, the two last patterns showed an earlier and more pronounced response in the SAPSII-low group. These results suggest that those genes may play a role in the very early negative feedback mechanisms that limit the process of inflammation during septic shock. Thus, the SAPSII-low group may exhibit a better and rapidly appropriate regulation of the immune and inflammatory response leading to a subsequent better outcome. This agrees with the observation that the number of significant upregulated genes is lower in the SAPSII-high group and increases during the kinetic while decreasing in the SAPSII-low group (Figure 4C).

These results of the utmost importance may suggest that regulatory mechanisms leading to recovery may take place very early after septic shock and thus that the time window to improve the outcome of patients with appropriate therapeutics may begin very early after the onset of shock. Indeed, beyond the fact that differences between the two severity groups were particularly noticeable in the degree and the duration of the altered acute inflammatory response, we observed that the more severe patients did not exhibit the strongest modulation for every process changed (e.g., cell death at H0) in part suggesting that an early and appropriate regulation may be the key for a suitable response. Thus, genes involved in these regulatory processes might be of major interest in additional studies.

There are some limitations of our study. First, our healthy volunteers’ cohort was not age/sex matched with patients (5 men and 20 women in healthy volunteers group). In our opinion, this is not a major issue as important results were mostly obtained from comparison between patients regarding kinetics approach. Second, as the SAPSII-high group tends to be older, the delayed and impaired immune response might have been related to age rather than severity. An additional analysis using ANOVA indicated that the modulation of gene expression was not related to age of patients (data not shown). Third, a reduced number of patients was enrolled and despite a high level of severity (median SAPSII = 45; median SOFA = 10), a low mortality rate was observed. We were therefore not able to study gene expression according to prognosis. Fourth, gene expression analyses were performed in whole blood samples that do not reveal the entire genomic dysfunctions leading to organ failure. Nevertheless, this compartment could indirectly reflect the damages observed in organs and is more easily accessible. In addition, there was a trend toward a lower number of polymorphonuclear cells in high SAPSII patients, but this was not statistically significant. Moreover, we did not found any difference between SAPSII-low and SAPSII-high groups regarding the lymphocyte counts. The differences observed and functions highlighted in our study are unlikely to be due to the differences in cell subpopulations between these groups. Lastly, no functional testing was done to validate that SAPSII-low group has a better integrity of their immune system or a better and earlier recovery. However, the results observed were coherent with findings already published in the literature. Although further studies will be needed to study deeper the mechanisms of systemic inflammatory response regulation (especially the link between genomic disturbances and immune functional testing), this study provides a first overview of the mechanisms involved at the earliest time and at the transcriptomic level after septic shock and may contribute to orientate the next investigations.

## Conclusions

Pan-genomic approaches, allowing global investigation, should provide crucial keys to understand the much focused orientation of the immune response induced by stress events.

These results could contribute to improved management of patients. Indeed, genes implicated very early in the regulatory mechanisms of the response to septic shock could become high medical value biomarkers allowing individualized treatments aimed at restricting the dysfunctions induced by exacerbated or uncontrolled inflammation.

## Additional files

Additional file 1: Table S1. Modulation over the first 48 h of septic shock for selected genes highlighted by the functional analysis. Additional file 2: Table S2. Significant category in septic shock patients over time compared to healthy volunteers. The table shows the predicted activation or inhibition status of the enriched functions. Additional file 3: Figure S1. qRT-PCR correlation with Affymetrix data. Additional file 4: Table S3. Significant ‘cell death’ functions in septic shock patients over time compared to healthy volunteers. Additional file 5: Table S4. Significant categories enriched by the 142 probe sets differentially expressed between. Additional file 6: Figure S2. Comparison of gene expression patterns of the 142 probe sets differentially expressed (FDR <0.05) between both groups of septic shock patients. Expressions at 0, 24, and 48 h after septic shock of genes in each of the 14 clusters from hierarchical clustering in Figure 6.
