# Supplementary material for: Early and dynamic changes in gene expression in septic shock patients: a genome-wide approach
Source: Intensive Care Med Exp. 2014 Aug 20;2:20. doi: 10.1186/s40635-014-0020-3 (PMC4512996; doi:10.1186/s40635-014-0020-3)
Supplement: Additional file 1: Table S1. — Modulation over the first 48 h of septic shock for selected genes highlighted by the functional analysis. [file 40635_2014_20_MOESM1_ESM.pdf]

Figure S2. qRT-PCR correlation with affymetrix data.

1- Correlation for IRAK-3 identified in the list of up regulated genes from the most increased canonical pathways enriched at H0, and HLA-DRA identified from the top 10 gene families suppressed after septic shock.

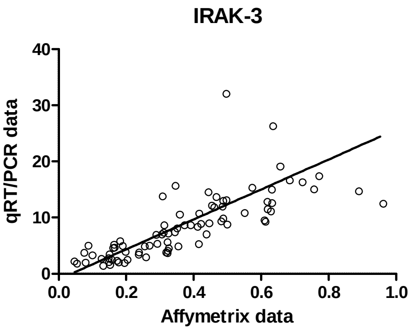

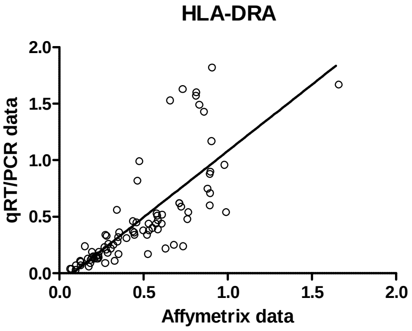

Spearman r	0,8858
95% confidence interval	0,826 to 0,926
P value (two-tailed)	< 0,0001
Number of XY Pairs	82

Spearman r	0,8864
95% confidence interval	0,827 to 0,926
P value (two-tailed)	< 0,0001
Number of XY Pairs	82

2- Correlation for the T cells transcription factor GATA3 and TBX21

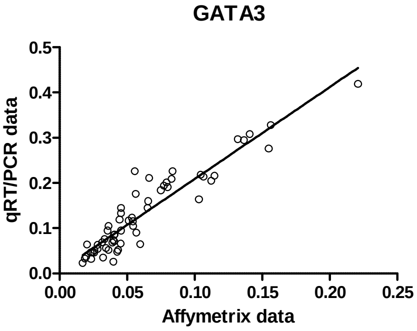

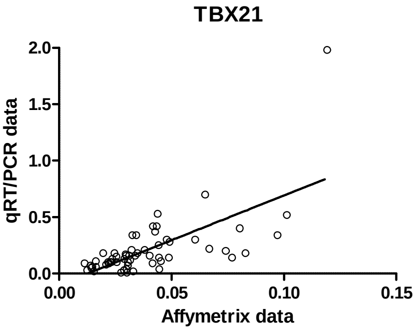

Spearman r	0,9148
95% confidence interval	0,858 to 0,949
P value (two-tailed)	< 0,0001
Number of XY Pairs	59

Spearman r	0,6718
95% confidence interval	0,496 to 0,795
P value (two-tailed)	< 0,0001
Number of XY Pairs	59

3- Correlation for CX3CR1 differentially expressed between SAPSII-high and -low group of patients

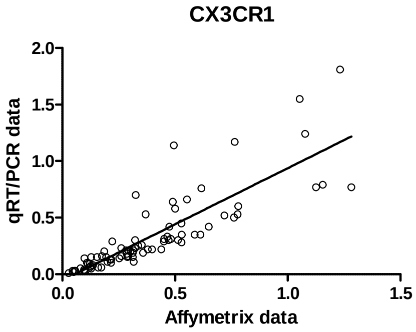

Spearman r	0,94
95% confidence interval	0,906 to 0,961
P value (two-tailed)	< 0,0001
Number of XY Pairs	82
