# Supplementary material for: Early and dynamic changes in gene expression in septic shock patients: a genome-wide approach
Source: Intensive Care Med Exp. 2014 Aug 20;2:20. doi: 10.1186/s40635-014-0020-3 (PMC4512996; doi:10.1186/s40635-014-0020-3)

Figure S1. Comparison of gene expression patterns of the 142 probesets differentially expressed (FDR <0.05) between both group of septic shock patients. Expression at 0, 24 and 48 hours after septic shock of genes in each of the 14 clusters from hierarchical clustering in Fig. 6.

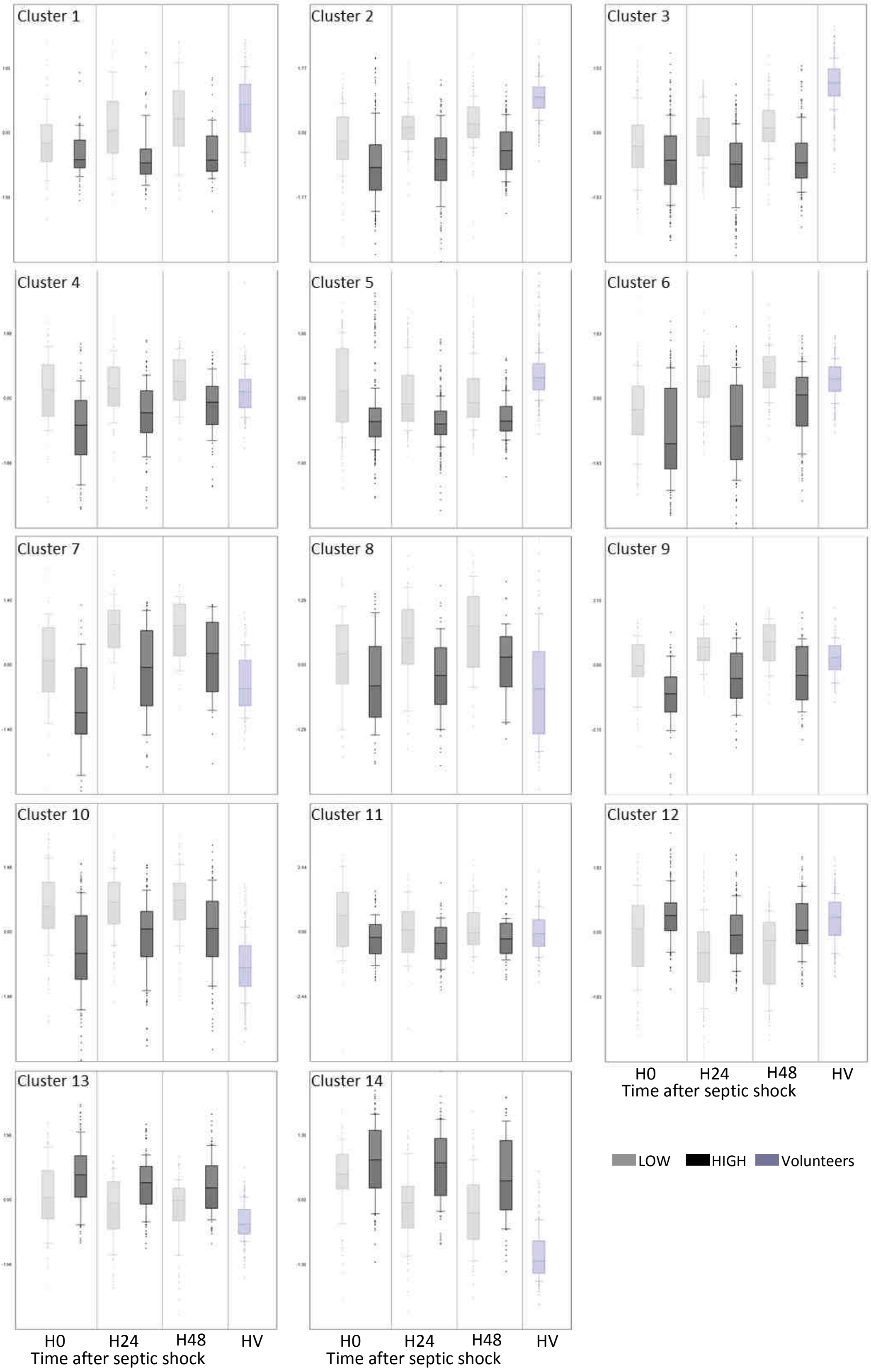

Supplement: Additional file 2: Table S2. — Significant category in septic shock patients over time compared to healthy volunteers. The table shows the predicted activation or inhibition status of the enriched functions. [file 40635_2014_20_MOESM2_ESM.pdf]
