# Supplementary material for: Early and dynamic changes in gene expression in septic shock patients: a genome-wide approach
Source: Intensive Care Med Exp. 2014 Aug 20;2:20. doi: 10.1186/s40635-014-0020-3 (PMC4512996; doi:10.1186/s40635-014-0020-3)
Supplement: Additional file 3: Figure S1. — qRT-PCR correlation with Affymetrix data. [file 40635_2014_20_MOESM3_ESM.pdf]

Supplementary Table 1: Modulation over the first 48h of septic shock for selected genes highlighted by the functional analysis.

genes	H0	H24	H48
CD247	-5.46	-6.55	-5.55
CD3D	-2.64	-2.56	-2.08
CD3E	-4.01	-4.21	-3.47
CD3G	-3.51	-3.20	-2.45
CD74	-2.24	-4.0	-2.27
CIITA	-2.38	-2.51	-2.33
CIITA	-2.38	-2.51	-2.33
HLA-DMA	-3.61	-3.14	-2.58
HLA-DMB	-4.43	-3.14	-2.88
HLA-DOA	-2.75	-2.67	-2.28
HLA-DOB	-2.62	-2.61	-2.17
HLA-DPA1	-4.54	-4.96	-4.16
HLA-DPB1	-4.17	-4.21	-3.73
HLA-DQA1	-3.45	-5.19	-3.14
HLA-DRA	-2.49	-2.96	-2.44
HLA-DRB1	-2.81	-3.35	-2.40
IFNGR1	2.21	2.68	2.3
IL10	2.41	1.99	1.61
IL18	1.85	2.17	2.25
IL18RAP	4.53	4.91	4.05
IL1R1	3.52	6.16	4.74
IL1R2	9.84	17.06	9.54
IL1RAP	2.173	NA	NA
IL1RN	2.09	1.56	1.76
IL4R	2.25	1.81	1.89
IRAK3	6.12	7.73	6.62
JAK2	2.46	2.32	2.07
JAK3	2.36	1.43	1.4
LY96	2.43	2.99	3.11
MAP2K6	2.79	2.9	2.33
MAPK1	-1.19	2.0	1.41
MAPK14	3.64	3.49	2.91
MMP9	8.2	7.51	6.75
NFKBIA	1.78	2.01	1.89
S100A8	2.26	2.24	2.11
SOCS3	2.73	3.25	2.57
TAP2	-2.28	-2.21	-2.10
TGFBR1	2.15	2.06	1.38
