# Supplementary material for: Early and dynamic changes in gene expression in septic shock patients: a genome-wide approach
Source: Intensive Care Med Exp. 2014 Aug 20;2:20. doi: 10.1186/s40635-014-0020-3 (PMC4512996; doi:10.1186/s40635-014-0020-3)
Supplement: Additional file 4: Table S3. — Significant ‘cell death’ functions in septic shock patients over time compared to healthy volunteers. [file 40635_2014_20_MOESM4_ESM.pdf]

Table S2. Significant category in septic shock patients over time compared to Healthy volunteers. The table shows the predicted activation or inhibition status of the enriched functions

© 2000-2012 Ingenuity Systems, Inc. All rights reserved.

Category	Functions Annotation	p-Value	Predicted Activation State	Regulation z-score	# Molecules
Inflammatory Response	immune response	6,67E-32		-0,35	316
Inflammatory Response	activation of leukocytes	2,94E-25		-1,92	154
Inflammatory Response	activation of mononuclear leukocytes	4,29E-18	Decreased	-2,82	110
Inflammatory Response	activation of lymphocytes	5,42E-17	Decreased	-2,56	104
Inflammatory Response	activation of T lymphocytes	8,77E-17	Decreased	-2,37	87
Inflammatory Response	inflammatory response	5,95E-15		1,69	141
Inflammatory Response	inflammation	9,12E-12		-0,15	117
Inflammatory Response	cell movement of phagocytes	3,51E-10		0,16	98
Inflammatory Response	cell-mediated response	1,40E-08		-1,23	42
Inflammatory Response	chemotaxis of phagocytes	2,71E-08		1,23	57
Inflammatory Response	immune response of cells	3,48E-08		1,68	50
Inflammatory Response	activation of phagocytes	3,55E-08		0,31	60
Inflammatory Response	activation of myeloid cells	4,09E-08		0,46	52
Inflammatory Response	cell movement of neutrophils	7,36E-08		0,84	58
Inflammatory Response	chemotaxis of neutrophils	2,40E-07		1,76	37
Inflammatory Response	recruitment of neutrophils	2,44E-07		1,54	36
Inflammatory Response	chemotaxis of granulocytes	3,23E-07		0,69	42
Inflammatory Response	quantity of phagocytes	6,14E-07	Decreased	-2,45	71
Inflammatory Response	chemotaxis of leukocytes	6,58E-07		0,82	63
Inflammatory Response	recruitment of phagocytes	1,26E-06		1,24	42
Inflammatory Response	degranulation of cells	1,37E-06		0,46	39
Inflammatory Response	antibody response	2,40E-06		-1,45	35
Inflammatory Response	activation of antigen presenting cells	2,51E-06		-0,63	45
Inflammatory Response	accumulation of leukocytes	2,86E-06		0,11	45
Inflammatory Response	chemotaxis of myeloid cells	4,37E-06		0,97	50
Inflammatory Response	migration of phagocytes	6,18E-06		-0,70	49
Inflammatory Response	antimicrobial response	6,40E-06		-0,78	38
Inflammatory Response	accumulation of myeloid cells	1,28E-05		0,02	31
Inflammatory Response	migration of neutrophils	1,35E-05		-0,16	25
Inflammatory Response	quantity of neutrophils	2,16E-05		-0,82	39
Inflammatory Response	phagocytosis of cells	3,74E-05	Increased	2,34	35
Inflammatory Response	phagocytosis	4,38E-05	Increased	2,05	42
Inflammatory Response	accumulation of phagocytes	1,05E-04		0,12	25
Inflammatory Response	immune response of tumor cell lines	1,36E-04		1,20	19
Inflammatory Response	degranulation of phagocytes	1,65E-04		0,79	25
Inflammatory Response	TH1 immune response	1,93E-04		-1,17	16
Inflammatory Response	accumulation of T lymphocytes	1,99E-04		-0,21	17
Inflammatory Response	antibody-dependent cell-mediated cytotoxicity	2,00E-04		-0,60	11
Inflammatory Response	response of phagocytes	2,00E-04		1,64	34
Inflammatory Response	function of immune system	2,29E-04			20
Inflammatory Response	activation of macrophages	2,50E-04		0,00	29
Inflammatory Response	quantity of blood platelets	2,61E-04		-0,04	23
Inflammatory Response	inflammation of organ	2,71E-04		-0,72	67
Amino Acid Metabolism	phosphorylation of L-amino acid	1,06E-05		-0,48	37
Amino Acid Metabolism	phosphorylation of L-tyrosine	8,33E-05		-0,38	28
Antigen Presentation	chemotaxis of phagocytes	2,71E-08		1,23	57
Antigen Presentation	activation of phagocytes	3,55E-08		0,31	60
Antigen Presentation	chemotaxis of neutrophils	2,40E-07		1,76	37
Antigen Presentation	activation of antigen presenting cells	2,51E-06		-0,63	45
Antigen Presentation	function of macrophages	1,53E-05			36
Antigen Presentation	activation of macrophages	2,50E-04		0,00	29
Antimicrobial Response	antimicrobial response	6,40E-06		-0,78	38
Cancer	neoplasia	5,60E-19		1,68	555
Cancer	cancer	1,41E-18		1,68	543
Cancer	tumorigenesis	2,01E-17		0,75	594
Cancer	carcinoma	1,12E-15		1,29	443
Cancer	solid tumor	1,97E-15		1,24	449
Cancer	hematological neoplasia	1,14E-10		-0,11	142
Cancer	genital tumor	1,94E-09		0,66	152
Cancer	colorectal cancer	3,23E-08		-0,59	141

Cancer	colorectal tumor	3,24E-08	-0,59	142
Cancer	gastrointestinal tract cancer	4,66E-08	-0,54	159
Cancer	lymphomagenesis	5,44E-08	-0,28	77
Cancer	lymphoid cancer	5,93E-08	-0,28	78
Cancer	lymphatic node tumor	8,08E-08	0,15	62
Cancer	non-Hodgkin's disease	8,83E-08	0,15	60
Cancer	digestive organ tumor	9,77E-08	0,27	218
Cancer	adenocarcinoma	1,31E-07	0,95	134
Cancer	renal cancer	1,31E-07		61
Cancer	B-cell non-Hodgkin's disease	4,68E-07	0,28	38
Cancer	growth of tumor	6,05E-07	-0,62	60
Cancer	lymphocytic leukemia	1,09E-06		42
Cancer	lung adenocarcinoma	1,62E-06	-0,14	32
Cancer	mammary tumor	2,85E-06	-1,30	153
Cancer	prostate cancer	3,81E-06	-0,19	84
Cancer	B-cell leukemia	4,24E-06		29
Cancer	prostatic tumor	4,56E-06	-0,32	85
Cancer	leukemia	7,03E-06	0,06	62
Cancer	metastasis	7,83E-06	1,16	81
Cancer	gonadal tumor	9,15E-06	1,08	81
Cancer	tumorigenesis of B-cell non-Hodgkin lymphon	1,17E-05	0,32	16
Cancer	diffuse large B-cell lymphoma	1,18E-05		12
Cancer	development of malignant tumor	1,62E-05	1,03	23
Cancer	uterine serous papillary cancer	1,63E-05		50
Cancer	hematologic cancer	1,78E-05	0,27	87
Cancer	head and neck cancer	2,52E-05	1,40	87
Cancer	squamous cell tumor	2,71E-05	1,19	56
Cancer	thyroid carcinoma	2,92E-05		23
Cancer	endometrial cancer	3,46E-05		64
Cancer	endometrial carcinoma	3,56E-05		68
Cancer	tumorigenesis of non-Hodgkin lymphoma	4,20E-05	-0,23	17
Cancer	squamous-cell carcinoma	6,04E-05	1,19	52
Cancer	cell transformation	6,34E-05	0,72	78
Cancer	tumorigenesis of lymphoma	7,45E-05	-0,40	22
Cancer	uterine cancer	8,84E-05		107
Cancer	chronic B-cell leukemia	8,90E-05		22
Cancer	breast cancer	9,17E-05	0,57	133
Cancer	differentiated thyroid cancer	9,26E-05		20
Cancer	tumorigenesis of thymus gland	1,07E-04	-1,00	4
Cancer	development of tumor	1,13E-04	0,57	35
Cancer	metastasis of tumor	1,19E-04	1,01	23
Cancer	thyroid cancer	1,22E-04		31
Cancer	hyperplasia of exocrine cells	1,67E-04	0,24	9
Cancer	papillary carcinoma	2,18E-04		21
Cancer	chronic leukemia	2,21E-04		29
Cancer	carcinoma in situ	2,24E-04		34
Cancer	transformation	2,39E-04	0,88	83
Cancer	quantity of tumor	2,41E-04	1,11	22
Cancer	papillary thyroid cancer	2,44E-04		17
Cancer	liver cancer	2,72E-04	0,49	69
Cancer	incidence of tumor	2,98E-04	0,57	46
Cancer	diffuse B-cell lymphoma	3,03E-04		13
Cardiovascular Disease	ischemic stroke	1,95E-06		15
Cardiovascular Disease	brain ischemia	3,21E-06		16
Cardiovascular Disease	cerebrovascular dysfunction	1,23E-05		19
Cardiovascular Disease	myocarditis	1,79E-05	0,64	12
Cardiovascular Disease	infarction	1,33E-04	0,77	46
Cardiovascular Disease	vascular disease	2,09E-04	0,96	94
Cardiovascular Disease	carditis	2,65E-04	0,20	14
Cardiovascular Disease	vascular lesion	3,21E-04	-1,00	31
Cardiovascular System Develop	mitosis of cardiomyocytes	1,09E-05	0,36	5
Cell Cycle	arrest in interphase	1,63E-06		71
Cell Cycle	cell cycle progression	3,39E-06	0,76	158
Cell Cycle	arrest in cell cycle progression	9,45E-06		59
Cell Cycle	mitosis of cardiomyocytes	1,09E-05	0,36	5
Cell Cycle	arrest in G2/M phase transition	1,17E-05		16
Cell Cycle	arrest in cell cycle progression of lymphocyte	2,38E-05		9

Cell Cycle	arrest in cell cycle progression of blood cells	2,38E-05			10
Cell Cycle	G1 phase of lymphoma cell lines	3,79E-05			8
Cell Cycle	cell division process of chromosomes	4,09E-05		0,95	46
Cell Cycle	G1 phase of brain cancer cell lines	7,07E-05			6
Cell Cycle	arrest in interphase of connective tissue cells	7,70E-05			13
Cell Cycle	arrest in cell cycle progression of thymocytes	1,07E-04			4
Cell Cycle	G2 phase of fibroblasts	1,08E-04			9
Cell Cycle	cell cycle progression of blood cells	1,12E-04		1,32	19
Cell Cycle	arrest in interphase of fibroblasts	1,45E-04			12
Cell Cycle	interphase of connective tissue cells	1,65E-04		0,83	18
Cell Cycle	G1 phase	1,86E-04		0,64	63
Cell Cycle	arrest in cell cycle progression of hematopoie	1,92E-04			5
Cell Cycle	mitosis of muscle cells	1,95E-04		0,12	8
Cell Cycle	interphase of brain cancer cell lines	1,95E-04		1,21	8
Cell Cycle	delay in mitosis of tumor cell lines	2,02E-04		-1,60	7
Cell Cycle	cell cycle progression of lymphocytes	2,65E-04		1,39	14
Cell Cycle	arrest in G1 phase	3,12E-04			43
Cell Death	cell death of immune cells	6,14E-23	Increased	2,33	150
Cell Death	cell death of blood cells	5,14E-22	Increased	2,11	153
Cell Death	cell death	1,43E-19		0,83	512
Cell Death	apoptosis of leukocytes	4,10E-17		1,48	106
Cell Death	apoptosis	5,80E-17		1,38	401
Cell Death	apoptosis of blood cells	9,20E-17		1,32	111
Cell Death	cell death of mononuclear leukocytes	4,52E-13	Increased	2,06	87
Cell Death	cell death of lymphocytes	1,42E-12		1,68	84
Cell Death	cell death of tumor cell lines	1,76E-12		0,89	218
Cell Death	cytotoxicity	4,16E-12		-0,74	57
Cell Death	cell death of T lymphocytes	6,14E-12		0,99	71
Cell Death	apoptosis of mononuclear leukocytes	8,15E-12		1,61	77
Cell Death	apoptosis of tumor cell lines	1,19E-11		1,46	188
Cell Death	apoptosis of lymphocytes	2,55E-11		1,24	74
Cell Death	cell death of leukemia cell lines	4,38E-11		1,01	65
Cell Death	apoptosis of T lymphocytes	5,66E-11		0,56	63
Cell Death	cytotoxicity of leukocytes	1,20E-10		-1,02	42
Cell Death	cytotoxicity of cells	2,10E-10		-0,80	52
Cell Death	cytotoxicity of lymphocytes	2,18E-09		-1,70	38
Cell Death	apoptosis of myeloid cells	9,54E-09		1,47	42
Cell Death	cell death of myeloid cells	1,13E-08	Increased	2,58	45
Cell Death	cell survival	1,30E-08		0,34	183
Cell Death	killing of cells	1,34E-08		-0,05	39
Cell Death	apoptosis of leukemia cell lines	1,43E-08		1,15	54
Cell Death	apoptosis of thymocytes	3,49E-08		0,43	33
Cell Death	cytotoxicity of natural killer cells	2,38E-07		-1,33	25
Cell Death	cytolysis	2,84E-07		-1,06	44
Cell Death	apoptosis of hematopoietic progenitor cells	4,02E-07		0,13	41
Cell Death	removal of cells	5,20E-07		1,45	20
Cell Death	cell death of thymocytes	5,23E-07		0,40	34
Cell Death	cell death of lymphoma cell lines	6,87E-07		0,63	40
Cell Death	apoptosis of hematopoietic cells	8,14E-07		0,23	42
Cell Death	killing of leukocytes	9,29E-07		-1,62	18
Cell Death	apoptosis of phagocytes	1,00E-06		0,90	39
Cell Death	apoptosis of lymphoid organ	1,08E-06		0,40	34
Cell Death	cell death of phagocytes	1,37E-06		1,92	41
Cell Death	clearance of cells	1,80E-06		1,22	18
Cell Death	cell death of lymphoid organ	4,89E-06		0,23	36
Cell Death	survival of blood cells	6,19E-06		-0,17	48
Cell Death	apoptosis of organ	7,67E-06		0,01	104
Cell Death	killing of lymphocytes	7,72E-06		-1,27	14
Cell Death	cell death of hematopoietic progenitor cells	9,52E-06		0,11	42
Cell Death	survival of leukocytes	1,10E-05		-0,27	44
Cell Death	cell death of granulocytes	1,27E-05		1,22	22
Cell Death	killing of T lymphocytes	1,43E-05		-1,61	10
Cell Death	cell death of hematopoietic cells	1,56E-05		0,21	43
Cell Death	cytolysis of blood cells	1,85E-05		-1,34	27
Cell Death	apoptosis of granulocytes	2,31E-05		0,37	21
Cell Death	cytolysis of lymphocytes	2,38E-05			22
Cell Death	survival of mononuclear leukocytes	5,94E-05		-0,57	33

Cell Death	apoptosis of lymphoma cell lines	9,45E-05	Increased	2,00	31
Cell Death	survival of cervical cancer cell lines	1,00E-04		-1,67	35
Cell Death	cell death of organ	1,04E-04		1,72	143
Cell Death	pyroptosis of bone marrow-derived macropha	1,07E-04		1,34	4
Cell Death	cytotoxicity of T lymphocytes	1,36E-04		-1,38	18
Cell Death	survival of lymphocytes	1,39E-04		-0,61	31
Cell Death	cytolysis of tumor cell lines	1,45E-04		-0,92	11
Cell Death	apoptosis of macrophages	1,61E-04		1,20	20
Cell Death	cell death of B lymphocytes	1,91E-04		0,55	25
Cell Death	cell death of macrophages	2,05E-04		1,96	22
Cell Death	apoptosis of B lymphocytes	2,61E-04		0,70	23
Cell Death	survival of tumor cell lines	2,70E-04		-1,09	74
Cell Death	activation-induced cell death	2,96E-04		0,06	17
Cell Death	apoptosis of breast cancer cell lines	3,12E-04		-0,32	43
Cell Morphology	morphology of leukocytes	2,69E-20			96
Cell Morphology	abnormal morphology of leukocytes	4,50E-20			75
Cell Morphology	morphology of mononuclear leukocytes	2,90E-19			65
Cell Morphology	morphology of lymphocytes	6,52E-18			61
Cell Morphology	morphology of blood cells	1,30E-17			105
Cell Morphology	abnormal morphology of lymphocytes	2,73E-16			53
Cell Morphology	morphology of T lymphocytes	1,43E-14			41
Cell Morphology	abnormal morphology of T lymphocytes	2,92E-13			36
Cell Morphology	lack of T lymphocytes	3,18E-09			15
Cell Morphology	morphology of cells	1,68E-08			234
Cell Morphology	morphology of B lymphocytes	4,36E-08			24
Cell Morphology	abnormal morphology of B lymphocytes	6,26E-06			16
Cell Morphology	lack of B lymphocytes	2,93E-05			13
Cell Morphology	abnormal morphology of cytotoxic T cells	3,84E-05			12
Cell Morphology	lack of CD4+ T-lymphocytes	7,07E-05			6
Cell Morphology	lack of Single positive thymocytes	1,07E-04			4
Cell Morphology	abnormal morphology of double-negative T ly	1,95E-04			8
Cell Morphology	morphology of hematopoietic progenitor cells	2,50E-04			29
Cell Signaling	tyrosine phosphorylation of protein	7,74E-09		-0,15	46
Cell Signaling	cell surface receptor linked signal transductio	1,84E-05	Increased	2,18	63
Cell Signaling	mobilization of Ca2+	6,22E-05		-0,85	59
Cell-mediated Immune Respons	T cell development	3,16E-22		-1,67	123
Cell-mediated Immune Respons	T cell homeostasis	1,13E-21		-1,72	124
Cell-mediated Immune Respons	differentiation of T lymphocytes	1,56E-16		-1,07	89
Cell-mediated Immune Respons	selection of T lymphocytes	1,10E-12		-1,16	24
Cell-mediated Immune Respons	selection of thymocytes	2,36E-12		-1,00	22
Cell-mediated Immune Respons	positive selection of T lymphocytes	3,03E-08		-0,89	12
Cell-mediated Immune Respons	positive selection of thymocytes	8,15E-08		-0,96	11
Cell-mediated Immune Respons	T cell migration	2,46E-07		-0,74	46
Cell-mediated Immune Respons	development of thymocytes	2,71E-07		-1,23	23
Cell-mediated Immune Respons	differentiation of helper T lymphocytes	1,18E-06		-0,13	36
Cell-mediated Immune Respons	cell movement of T lymphocytes	2,88E-06		-0,16	39
Cell-mediated Immune Respons	negative selection of T lymphocytes	3,25E-06	Decreased	-2,06	9
Cell-mediated Immune Respons	adhesion of T lymphocytes	1,27E-05		0,68	22
Cell-mediated Immune Respons	development of helper T lymphocytes	2,10E-05		-0,99	16
Cell-mediated Immune Respons	negative selection of thymocytes	2,55E-05		-1,79	7
Cell-mediated Immune Respons	differentiation of Th2 cells	3,98E-05		-0,22	19
Cell-mediated Immune Respons	arrest in differentiation of T lymphocytes	1,03E-04			11
Cell-To-Cell Signaling and Intera	activation of cells	1,21E-25		-0,33	200
Cell-To-Cell Signaling and Intera	activation of blood cells	1,74E-25		-1,22	168
Cell-To-Cell Signaling and Intera	activation of leukocytes	2,94E-25		-1,92	154
Cell-To-Cell Signaling and Intera	activation of mononuclear leukocytes	4,29E-18	Decreased	-2,82	110
Cell-To-Cell Signaling and Intera	activation of lymphocytes	5,42E-17	Decreased	-2,56	104
Cell-To-Cell Signaling and Intera	activation of T lymphocytes	8,77E-17	Decreased	-2,37	87
Cell-To-Cell Signaling and Intera	selection of T lymphocytes	1,10E-12		-1,16	24
Cell-To-Cell Signaling and Intera	selection of thymocytes	2,36E-12		-1,00	22
Cell-To-Cell Signaling and Intera	response of leukocytes	2,20E-11		1,11	76
Cell-To-Cell Signaling and Intera	selection of cells	5,03E-11		-0,89	25
Cell-To-Cell Signaling and Intera	response of blood cells	7,59E-11		1,25	77
Cell-To-Cell Signaling and Intera	binding of lymphocytes	2,20E-10		-1,19	31
Cell-To-Cell Signaling and Intera	binding of mononuclear leukocytes	2,89E-10		-0,76	35
Cell-To-Cell Signaling and Intera	binding of blood cells	2,90E-10		-0,46	56
Cell-To-Cell Signaling and Intera	binding of leukocytes	1,72E-09		-0,67	47

Cell-To-Cell Signaling and Intera	positive selection of cells	4,08E-09		-0,66	14
Cell-To-Cell Signaling and Intera	binding of cells	7,29E-09		0,78	95
Cell-To-Cell Signaling and Intera	positive selection of lymphocytes	1,12E-08		-0,39	13
Cell-To-Cell Signaling and Intera	response of mononuclear leukocytes	2,54E-08		0,12	47
Cell-To-Cell Signaling and Intera	positive selection of T lymphocytes	3,03E-08		-0,89	12
Cell-To-Cell Signaling and Intera	activation of phagocytes	3,55E-08		0,31	60
Cell-To-Cell Signaling and Intera	activation of myeloid cells	4,09E-08		0,46	52
Cell-To-Cell Signaling and Intera	recruitment of granulocytes	4,96E-08		0,87	43
Cell-To-Cell Signaling and Intera	adhesion of blood cells	5,16E-08		0,07	73
Cell-To-Cell Signaling and Intera	positive selection of thymocytes	8,15E-08		-0,96	11
Cell-To-Cell Signaling and Intera	recruitment of neutrophils	2,44E-07		1,54	36
Cell-To-Cell Signaling and Intera	binding of T lymphocytes	2,79E-07		-1,58	22
Cell-To-Cell Signaling and Intera	recruitment of cells	4,29E-07		-0,19	60
Cell-To-Cell Signaling and Intera	recruitment of blood cells	5,34E-07		-0,13	56
Cell-To-Cell Signaling and Intera	recruitment of leukocytes	5,96E-07		-0,03	55
Cell-To-Cell Signaling and Intera	T cell response	8,57E-07		-0,23	28
Cell-To-Cell Signaling and Intera	interaction of T lymphocytes	1,07E-06	Decreased	-2,00	25
Cell-To-Cell Signaling and Intera	interaction of leukocytes	1,17E-06		-1,77	26
Cell-To-Cell Signaling and Intera	recruitment of phagocytes	1,26E-06		1,24	42
Cell-To-Cell Signaling and Intera	adhesion of immune cells	2,10E-06		0,14	63
Cell-To-Cell Signaling and Intera	activation of antigen presenting cells	2,51E-06		-0,63	45
Cell-To-Cell Signaling and Intera	stimulation of blood cells	2,65E-06		-0,67	36
Cell-To-Cell Signaling and Intera	response of lymphocytes	2,75E-06		-0,24	32
Cell-To-Cell Signaling and Intera	negative selection of T lymphocytes	3,25E-06	Decreased	-2,06	9
Cell-To-Cell Signaling and Intera	stimulation of leukocytes	3,30E-06		-0,83	35
Cell-To-Cell Signaling and Intera	stimulation of T lymphocytes	3,87E-06		-1,30	24
Cell-To-Cell Signaling and Intera	stimulation of cells	4,85E-06		-0,95	52
Cell-To-Cell Signaling and Intera	adhesion of T lymphocytes	1,27E-05		0,68	22
Cell-To-Cell Signaling and Intera	stimulation of lymphocytes	1,60E-05		-1,20	26
Cell-To-Cell Signaling and Intera	binding of tumor cell lines	1,93E-05		1,04	35
Cell-To-Cell Signaling and Intera	negative selection of thymocytes	2,55E-05		-1,79	7
Cell-To-Cell Signaling and Intera	response of tumor cell lines	3,49E-05		1,04	21
Cell-To-Cell Signaling and Intera	phagocytosis of cells	3,74E-05	Increased	2,34	35
Cell-To-Cell Signaling and Intera	interaction of cells	4,22E-05		-1,75	27
Cell-To-Cell Signaling and Intera	response of antigen presenting cells	5,04E-05		1,14	32
Cell-To-Cell Signaling and Intera	adhesion of lymphocytes	8,15E-05		0,56	24
Cell-To-Cell Signaling and Intera	adhesion of mononuclear leukocytes	1,10E-04		0,32	28
Cell-To-Cell Signaling and Intera	binding of B lymphocytes	1,19E-04		-0,65	8
Cell-To-Cell Signaling and Intera	immune response of tumor cell lines	1,36E-04		1,20	19
Cell-To-Cell Signaling and Intera	response of phagocytes	2,00E-04		1,64	34
Cell-To-Cell Signaling and Intera	activation of macrophages	2,50E-04		0,00	29
Cell-To-Cell Signaling and Intera	binding of granulocytes	2,84E-04		-0,28	15
Cellular Assembly and Organizat	rearrangement of microtubule organizing cent	1,07E-04		-1,78	4
Cellular Compromise	degranulation of cells	1,37E-06		0,46	39
Cellular Compromise	degranulation of phagocytes	1,65E-04		0,79	25
Cellular Development	development of mononuclear leukocytes	3,39E-24		-1,85	137
Cellular Development	developmental process of lymphocytes	3,58E-24	Decreased	-2,38	157
Cellular Development	development of leukocytes	3,97E-24		-1,82	142
Cellular Development	developmental process of T lymphocytes	5,56E-24	Decreased	-2,46	133
Cellular Development	development of lymphocytes	1,33E-23		-1,82	135
Cellular Development	development of blood cells	6,16E-23		-1,97	152
Cellular Development	T cell development	3,16E-22		-1,67	123
Cellular Development	differentiation of mononuclear leukocytes	5,84E-22		-1,93	131
Cellular Development	differentiation of blood cells	6,55E-22		-1,78	164
Cellular Development	differentiation of lymphocytes	9,70E-22		-1,87	123
Cellular Development	differentiation of leukocytes	1,50E-21		-1,69	148
Cellular Development	differentiation of T lymphocytes	1,56E-16		-1,07	89
Cellular Development	selection of T lymphocytes	1,10E-12		-1,16	24
Cellular Development	selection of thymocytes	2,36E-12		-1,00	22
Cellular Development	developmental process of thymocytes	4,02E-11		-1,91	45
Cellular Development	differentiation of cells	2,21E-10		-1,60	281
Cellular Development	differentiation	2,36E-09		-1,54	292
Cellular Development	growth of blood cells	1,55E-08		-0,56	55
Cellular Development	growth of immune cells	1,95E-08		-1,46	49
Cellular Development	positive selection of T lymphocytes	3,03E-08		-0,89	12
Cellular Development	positive selection of thymocytes	8,15E-08		-0,96	11
Cellular Development	development of thymocytes	2,71E-07		-1,23	23

Cellular Development	expansion of blood cells	3,43E-07		-1,34	41
Cellular Development	expansion of leukocytes	6,85E-07	Decreased	-2,22	37
Cellular Development	differentiation of helper T lymphocytes	1,18E-06		-0,13	36
Cellular Development	developmental process of helper T lymphocyt	1,59E-06		-0,24	39
Cellular Development	growth of prostate cancer cell lines	2,37E-06		-1,10	31
Cellular Development	development of hematopoietic cells	2,48E-06		-1,92	37
Cellular Development	developmental process of B lymphocytes	2,64E-06		-0,77	57
Cellular Development	expansion of mononuclear leukocytes	2,65E-06		-1,85	33
Cellular Development	developmental process of cytotoxic T cells	2,72E-06		-0,07	13
Cellular Development	growth of lymphocytes	2,89E-06		-1,67	37
Cellular Development	growth of mononuclear leukocytes	3,12E-06		-1,53	38
Cellular Development	negative selection of T lymphocytes	3,25E-06	Decreased	-2,06	9
Cellular Development	differentiation of hematopoietic cells	3,86E-06	Decreased	-2,01	48
Cellular Development	expansion of lymphocytes	3,87E-06	Decreased	-2,09	32
Cellular Development	arrest in developmental process of lymphocyt	5,64E-06			23
Cellular Development	proliferation of hematopoietic cells	6,10E-06		-1,12	40
Cellular Development	proliferation of hematopoietic progenitor cells	6,70E-06		-1,29	39
Cellular Development	arrest in differentiation of lymphocytes	1,23E-05			19
Cellular Development	expansion of T lymphocytes	1,56E-05	Decreased	-2,68	27
Cellular Development	differentiation of hematopoietic progenitor cell	1,56E-05	Decreased	-2,15	45
Cellular Development	development of hematopoietic progenitor cells	1,59E-05		-1,93	34
Cellular Development	development of helper T lymphocytes	2,10E-05		-0,99	16
Cellular Development	growth of T lymphocytes	2,32E-05	Decreased	-2,41	29
Cellular Development	negative selection of thymocytes	2,55E-05		-1,79	7
Cellular Development	developmental process of Th2 cells	2,84E-05		-0,23	21
Cellular Development	developmental process of alpha-beta T lymph	3,83E-05		-0,72	10
Cellular Development	differentiation of B lymphocytes	3,95E-05		-0,67	41
Cellular Development	differentiation of Th2 cells	3,98E-05		-0,22	19
Cellular Development	arrest in differentiation of cells	7,60E-05			21
Cellular Development	growth of tumor cell lines	9,96E-05	Decreased	-2,17	118
Cellular Development	arrest in differentiation of T lymphocytes	1,03E-04			11
Cellular Development	developmental process of pre-B lymphocytes	1,63E-04		-1,50	17
Cellular Development	arrest in developmental process of B lymphoc	1,63E-04			14
Cellular Development	maturation of leukocytes	1,69E-04	Decreased	-2,25	38
Cellular Development	developmental process of pro-B lymphocytes	2,35E-04		-1,60	13
Cellular Development	arrest in developmental process of T lymphoc	2,35E-04			13
Cellular Development	differentiation of pre-B lymphocytes	2,73E-04		-1,32	10
Cellular Function and Maintenan	function of blood cells	4,30E-27		-1,34	151
Cellular Function and Maintenan	function of leukocytes	2,03E-26		-1,78	143
Cellular Function and Maintenan	homeostasis of leukocytes	5,21E-25		-1,67	136
Cellular Function and Maintenan	Lymphocyte homeostasis	5,31E-25		-1,67	135
Cellular Function and Maintenan	T cell development	3,16E-22		-1,67	123
Cellular Function and Maintenan	T cell homeostasis	1,13E-21		-1,72	124
Cellular Function and Maintenan	function of lymphocytes	3,44E-19		-0,51	90
Cellular Function and Maintenan	function of T lymphocytes	2,46E-17		-0,66	74
Cellular Function and Maintenan	differentiation of T lymphocytes	1,56E-16		-1,07	89
Cellular Function and Maintenan	cellular homeostasis	8,21E-14		-1,44	227
Cellular Function and Maintenan	selection of T lymphocytes	1,10E-12		-1,16	24
Cellular Function and Maintenan	selection of thymocytes	2,36E-12		-1,00	22
Cellular Function and Maintenan	function of phagocytes	4,59E-11	Decreased	-2,42	70
Cellular Function and Maintenan	function of CD4+ T-lymphocytes	7,99E-11			25
Cellular Function and Maintenan	function of myeloid cells	1,69E-09		-1,79	55
Cellular Function and Maintenan	positive selection of cells	4,08E-09		-0,66	14
Cellular Function and Maintenan	positive selection of lymphocytes	1,12E-08		-0,39	13
Cellular Function and Maintenan	function of helper T lymphocytes	2,35E-08			25
Cellular Function and Maintenan	positive selection of T lymphocytes	3,03E-08		-0,89	12
Cellular Function and Maintenan	positive selection of thymocytes	8,15E-08		-0,96	11
Cellular Function and Maintenan	function of antigen presenting cells	1,30E-07	Decreased	-2,06	52
Cellular Function and Maintenan	development of thymocytes	2,71E-07		-1,23	23
Cellular Function and Maintenan	function of granulocytes	5,11E-07			27
Cellular Function and Maintenan	differentiation of helper T lymphocytes	1,18E-06		-0,13	36
Cellular Function and Maintenan	clearance of cells	1,80E-06		1,22	18
Cellular Function and Maintenan	function of Th1 cells	2,25E-06			16
Cellular Function and Maintenan	negative selection of T lymphocytes	3,25E-06	Decreased	-2,06	9
Cellular Function and Maintenan	function of Th2 cells	5,96E-06			15
Cellular Function and Maintenan	function of macrophages	1,53E-05			36
Cellular Function and Maintenan	function of neutrophils	1,86E-05			21

Cellular Function and Maintenan	development of helper T lymphocytes	2,10E-05		-0,99	16
Cellular Function and Maintenan	negative selection of thymocytes	2,55E-05		-1,79	7
Cellular Function and Maintenan	phagocytosis of cells	3,74E-05	Increased	2,34	35
Cellular Function and Maintenan	differentiation of Th2 cells	3,98E-05		-0,22	19
Cellular Function and Maintenan	phagocytosis	4,38E-05	Increased	2,05	42
Cellular Function and Maintenan	arrest in differentiation of T lymphocytes	1,03E-04			11
Cellular Function and Maintenan	engulfment of cells	1,39E-04	Increased	2,26	37
Cellular Function and Maintenan	function of natural killer cells	1,62E-04			6
Cellular Function and Maintenan	function of dendritic cells	2,18E-04			21
Cellular Growth and Proliferation	proliferation of immune cells	1,50E-23	Decreased	-2,09	169
Cellular Growth and Proliferation	proliferation of blood cells	2,31E-23	Decreased	-2,20	176
Cellular Growth and Proliferation	proliferation of mononuclear leukocytes	2,71E-23		-1,98	163
Cellular Growth and Proliferation	proliferation of lymphocytes	8,24E-23		-1,81	161
Cellular Growth and Proliferation	proliferation of T lymphocytes	3,43E-19		-1,51	132
Cellular Growth and Proliferation	proliferation of cells	5,15E-15		0,78	410
Cellular Growth and Proliferation	proliferation of B lymphocytes	1,55E-10		-1,05	63
Cellular Growth and Proliferation	growth of blood cells	1,55E-08		-0,56	55
Cellular Growth and Proliferation	growth of immune cells	1,95E-08		-1,46	49
Cellular Growth and Proliferation	expansion of blood cells	3,43E-07		-1,34	41
Cellular Growth and Proliferation	growth of cells	4,16E-07		-1,02	264
Cellular Growth and Proliferation	generation of T lymphocytes	5,16E-07		-1,83	25
Cellular Growth and Proliferation	expansion of leukocytes	6,85E-07	Decreased	-2,22	37
Cellular Growth and Proliferation	expansion of cells	8,46E-07		-0,57	48
Cellular Growth and Proliferation	generation of lymphocytes	1,06E-06		-1,74	28
Cellular Growth and Proliferation	growth of prostate cancer cell lines	2,37E-06		-1,10	31
Cellular Growth and Proliferation	expansion of mononuclear leukocytes	2,65E-06		-1,85	33
Cellular Growth and Proliferation	stimulation of blood cells	2,65E-06		-0,67	36
Cellular Growth and Proliferation	growth of lymphocytes	2,89E-06		-1,67	37
Cellular Growth and Proliferation	growth of mononuclear leukocytes	3,12E-06		-1,53	38
Cellular Growth and Proliferation	stimulation of leukocytes	3,30E-06		-0,83	35
Cellular Growth and Proliferation	expansion of lymphocytes	3,87E-06	Decreased	-2,09	32
Cellular Growth and Proliferation	stimulation of T lymphocytes	3,87E-06		-1,30	24
Cellular Growth and Proliferation	stimulation of cells	4,85E-06		-0,95	52
Cellular Growth and Proliferation	proliferation of hematopoietic cells	6,10E-06		-1,12	40
Cellular Growth and Proliferation	proliferation of hematopoietic progenitor cells	6,70E-06		-1,29	39
Cellular Growth and Proliferation	generation of helper T lymphocytes	9,73E-06	Decreased	-2,37	13
Cellular Growth and Proliferation	generation of leukocytes	1,02E-05	Decreased	-2,09	29
Cellular Growth and Proliferation	proliferation of tumor cell lines	1,28E-05	Increased	3,29	131
Cellular Growth and Proliferation	expansion of T lymphocytes	1,56E-05	Decreased	-2,68	27
Cellular Growth and Proliferation	stimulation of lymphocytes	1,60E-05		-1,20	26
Cellular Growth and Proliferation	growth of T lymphocytes	2,32E-05	Decreased	-2,41	29
Cellular Growth and Proliferation	proliferation of splenocytes	5,12E-05		-1,00	20
Cellular Growth and Proliferation	growth of tumor cell lines	9,96E-05	Decreased	-2,17	118
Cellular Growth and Proliferation	proliferation of lymphatic system cells	1,10E-04		-0,78	32
Cellular Growth and Proliferation	proliferation of tumor cells	1,19E-04		0,88	40
Cellular Growth and Proliferation	generation of Th1 cells	1,62E-04		-1,93	6
Cellular Movement	cell movement of leukocytes	1,12E-13		-0,49	141
Cellular Movement	cell movement of blood cells	1,61E-13		-0,24	157
Cellular Movement	leukocyte migration	2,77E-13		-0,23	156
Cellular Movement	cell movement of mononuclear leukocytes	1,04E-11		-0,75	93
Cellular Movement	migration of mononuclear leukocytes	3,47E-11		-0,91	74
Cellular Movement	cell movement of granulocytes	1,53E-10		-0,06	77
Cellular Movement	cell movement of myeloid cells	1,79E-10		-0,15	98
Cellular Movement	Lymphocyte migration	3,10E-10		-1,23	67
Cellular Movement	cell movement of phagocytes	3,51E-10		0,16	98
Cellular Movement	cell movement of lymphocytes	3,84E-10		-1,53	76
Cellular Movement	infiltration of cells	1,81E-09		-0,72	75
Cellular Movement	infiltration of blood cells	3,57E-09		-0,58	71
Cellular Movement	infiltration of leukocytes	7,55E-09		-0,49	70
Cellular Movement	chemotaxis of phagocytes	2,71E-08		1,23	57
Cellular Movement	migration of cells	4,20E-08		0,52	244
Cellular Movement	homing of leukocytes	4,68E-08		0,47	69
Cellular Movement	recruitment of granulocytes	4,96E-08		0,87	43
Cellular Movement	cell movement of neutrophils	7,36E-08		0,84	58
Cellular Movement	chemotaxis of neutrophils	2,40E-07		1,76	37
Cellular Movement	recruitment of neutrophils	2,44E-07		1,54	36
Cellular Movement	T cell migration	2,46E-07		-0,74	46

Cellular Movement	chemotaxis of granulocytes	3,23E-07		0,69	42
Cellular Movement	cell movement	3,61E-07		1,08	263
Cellular Movement	recruitment of cells	4,29E-07		-0,19	60
Cellular Movement	recruitment of blood cells	5,34E-07		-0,13	56
Cellular Movement	recruitment of leukocytes	5,96E-07		-0,03	55
Cellular Movement	chemotaxis of leukocytes	6,58E-07		0,82	63
Cellular Movement	recruitment of phagocytes	1,26E-06		1,24	42
Cellular Movement	infiltration of myeloid cells	1,68E-06		-0,37	42
Cellular Movement	cell movement of T lymphocytes	2,88E-06		-0,16	39
Cellular Movement	infiltration of granulocytes	3,12E-06		-0,15	38
Cellular Movement	migration of myeloid cells	4,35E-06		-1,19	33
Cellular Movement	chemotaxis of myeloid cells	4,37E-06		0,97	50
Cellular Movement	migration of phagocytes	6,18E-06		-0,70	49
Cellular Movement	homing of cells	6,21E-06		1,06	85
Cellular Movement	migration of granulocytes	9,28E-06		-1,23	31
Cellular Movement	chemotaxis of cells	1,05E-05		1,51	80
Cellular Movement	cell movement of antigen presenting cells	1,15E-05		-1,67	52
Cellular Movement	adhesion of T lymphocytes	1,27E-05		0,68	22
Cellular Movement	migration of neutrophils	1,35E-05		-0,16	25
Cellular Movement	homing	1,65E-05		1,07	86
Cellular Movement	homing of lymphocytes	2,94E-05		-1,80	30
Cellular Movement	chemotaxis	3,45E-05		1,52	81
Cellular Movement	cell movement of eosinophils	7,21E-05		-1,26	23
Cellular Movement	homing of mononuclear leukocytes	8,90E-05		-0,65	37
Cellular Movement	influx of leukocytes	2,28E-04		0,18	15
Cellular Movement	transmigration of granulocytes	2,35E-04		-1,09	13
Cellular Movement	transmigration of leukocytes	2,61E-04		-1,36	23
Cellular Movement	transmigration of cells	2,91E-04		-1,65	25
Cellular Response to Therapeuti	radiosensitivity of cancer cells	1,07E-04		-1,79	4
Connective Tissue Development	G2 phase of fibroblasts	1,08E-04			9
Connective Tissue Disorders	rheumatic disease	6,77E-22		0,03	224
Connective Tissue Disorders	arthritis	7,79E-18		0,25	199
Connective Tissue Disorders	rheumatoid arthritis	6,20E-14		-1,89	150
Connective Tissue Disorders	systemic lupus erythematosus	4,33E-09			38
Connective Tissue Disorders	juvenile rheumatoid arthritis	2,93E-06			29
Connective Tissue Disorders	psoriatic arthritis	4,63E-06			21
Connective Tissue Disorders	polyarticular juvenile rheumatoid arthritis	1,08E-05			23
Connective Tissue Disorders	collagen-induced arthritis	8,90E-05		-0,87	22
Connective Tissue Disorders	experimentally-induced arthritis	1,45E-04		-0,41	28
Connective Tissue Disorders	damage of connective tissue	2,24E-04		0,27	23
Dermatological Diseases and Co	dermatitis	4,45E-08		-0,25	76
Dermatological Diseases and Co	atopic dermatitis	8,63E-08			59
Dermatological Diseases and Co	tuberculoid leprosy	1,93E-07			13
Dermatological Diseases and Co	leprosy	2,38E-07			25
Dermatological Diseases and Co	psoriasis	4,41E-06			100
Dermatological Diseases and Co	psoriatic arthritis	4,63E-06			21
Dermatological Diseases and Co	acne	1,06E-04			22
Developmental Disorder	hypoplasia of lymphoid organ	3,64E-08			39
Developmental Disorder	hypoplasia of spleen	1,74E-05			20
Developmental Disorder	hypoplasia of thymus gland	2,40E-05			24
Developmental Disorder	hypoplasia of organ	1,35E-04		-1,00	50
Drug Metabolism	quantity of glutathione	2,39E-04		-0,16	16
Embryonic Development	development of lymphoid organ	2,71E-07	Decreased	-2,02	50
Embryonic Development	development of thymocytes	2,71E-07		-1,23	23
Embryonic Development	development of thymus gland	5,77E-05		-1,22	27
Embryonic Development	morphology of germinal center	1,32E-04			17
Embryonic Development	abnormal morphology of germinal center	1,63E-04			14
Endocrine System Disorders	thyroid carcinoma	2,92E-05			23
Endocrine System Disorders	differentiated thyroid cancer	9,26E-05			20
Endocrine System Disorders	thyroid cancer	1,22E-04			31
Endocrine System Disorders	papillary thyroid cancer	2,44E-04			17
Gastrointestinal Disease	colitis	1,13E-08		-0,90	45
Gastrointestinal Disease	colorectal cancer	3,23E-08		-0,59	141
Gastrointestinal Disease	colorectal tumor	3,24E-08		-0,59	142
Gastrointestinal Disease	gastrointestinal tract cancer	4,66E-08		-0,54	159
Gastrointestinal Disease	digestive organ tumor	9,77E-08		0,27	218
Gastrointestinal Disease	ulcerative colitis	9,66E-07			20

Gastrointestinal Disease	inflammatory bowel disease	3,40E-06		-0,70	60
Gastrointestinal Disease	liver cancer	2,72E-04		0,49	69
Gene Expression	binding of protein binding site	2,41E-05		0,50	54
Gene Expression	binding of DNA	1,28E-04		0,93	86
Genetic Disorder	immediate hypersensitivity	8,71E-09		0,91	71
Genetic Disorder	colitis	1,13E-08		-0,90	45
Genetic Disorder	atopic dermatitis	8,63E-08			59
Genetic Disorder	tuberculoid leprosy	1,93E-07			13
Genetic Disorder	leprosy	2,38E-07			25
Genetic Disorder	ulcerative colitis	9,66E-07			20
Genetic Disorder	lung adenocarcinoma	1,62E-06		-0,14	32
Genetic Disorder	ischemic stroke	1,95E-06			15
Genetic Disorder	inflammatory bowel disease	3,40E-06		-0,70	60
Genetic Disorder	prostate cancer	3,81E-06		-0,19	84
Genetic Disorder	psoriasis	4,41E-06			100
Genetic Disorder	psoriatic arthritis	4,63E-06			21
Hematological Disease	hematological neoplasia	1,14E-10		-0,11	142
Hematological Disease	eosinophilia	4,57E-08	Decreased	-2,40	31
Hematological Disease	lymphomagenesis	5,44E-08		-0,28	77
Hematological Disease	non-Hodgkin's disease	8,83E-08		0,15	60
Hematological Disease	B-cell non-Hodgkin's disease	4,68E-07		0,28	38
Hematological Disease	lymphocytic leukemia	1,09E-06			42
Hematological Disease	ischemic stroke	1,95E-06			15
Hematological Disease	brain ischemia	3,21E-06			16
Hematological Disease	B-cell leukemia	4,24E-06			29
Hematological Disease	leukemia	7,03E-06		0,06	62
Hematological Disease	tumorigenesis of B-cell non-Hodgkin lymphon	1,17E-05		0,32	16
Hematological Disease	diffuse large B-cell lymphoma	1,18E-05			12
Hematological Disease	hematologic cancer	1,78E-05		0,27	87
Hematological Disease	tumorigenesis of non-Hodgkin lymphoma	4,20E-05		-0,23	17
Hematological Disease	tumorigenesis of lymphoma	7,45E-05		-0,40	22
Hematological Disease	chronic B-cell leukemia	8,90E-05			22
Hematological Disease	chronic leukemia	2,21E-04			29
Hematological Disease	diffuse B-cell lymphoma	3,03E-04			13
Hematological System Developn	quantity of leukocytes	8,73E-29	Decreased	-4,69	208
Hematological System Developn	quantity of blood cells	1,04E-27	Decreased	-4,76	225
Hematological System Developn	quantity of mononuclear leukocytes	6,47E-26	Decreased	-5,18	172
Hematological System Developn	activation of blood cells	1,74E-25		-1,22	168
Hematological System Developn	activation of leukocytes	2,94E-25		-1,92	154
Hematological System Developn	hematopoiesis	7,43E-25		-1,61	200
Hematological System Developn	quantity of lymphocytes	2,35E-24	Decreased	-4,80	164
Hematological System Developn	development of mononuclear leukocytes	3,39E-24		-1,85	137
Hematological System Developn	development of leukocytes	3,97E-24		-1,82	142
Hematological System Developn	development of lymphocytes	1,33E-23		-1,82	135
Hematological System Developn	proliferation of immune cells	1,50E-23	Decreased	-2,09	169
Hematological System Developn	proliferation of mononuclear leukocytes	2,71E-23		-1,98	163
Hematological System Developn	development of blood cells	6,16E-23		-1,97	152
Hematological System Developn	proliferation of lymphocytes	8,24E-23		-1,81	161
Hematological System Developn	T cell development	3,16E-22		-1,67	123
Hematological System Developn	differentiation of mononuclear leukocytes	5,84E-22		-1,93	131
Hematological System Developn	differentiation of blood cells	6,55E-22		-1,78	164
Hematological System Developn	differentiation of lymphocytes	9,70E-22		-1,87	123
Hematological System Developn	T cell homeostasis	1,13E-21		-1,72	124
Hematological System Developn	differentiation of leukocytes	1,50E-21		-1,69	148
Hematological System Developn	quantity of T lymphocytes	2,45E-21	Decreased	-4,33	127
Hematological System Developn	proliferation of T lymphocytes	3,43E-19		-1,51	132
Hematological System Developn	function of lymphocytes	3,44E-19		-0,51	90
Hematological System Developn	activation of mononuclear leukocytes	4,29E-18	Decreased	-2,82	110
Hematological System Developn	function of T lymphocytes	2,46E-17		-0,66	74
Hematological System Developn	activation of lymphocytes	5,42E-17	Decreased	-2,56	104
Hematological System Developn	activation of T lymphocytes	8,77E-17	Decreased	-2,37	87
Hematological System Developn	differentiation of T lymphocytes	1,56E-16		-1,07	89
Hematological System Developn	quantity of helper T lymphocytes	3,75E-16	Decreased	-2,95	54
Hematological System Developn	morphology of T lymphocytes	1,43E-14			41
Hematological System Developn	quantity of cytotoxic T cells	9,67E-14		-0,56	43
Hematological System Developn	cell movement of leukocytes	1,12E-13		-0,49	141
Hematological System Developn	abnormal morphology of T lymphocytes	2,92E-13			36

Hematological System Developn	quantity of hematopoietic cells	7,26E-13	Decreased	-2,94	98
Hematological System Developn	quantity of hematopoietic progenitor cells	1,06E-12	Decreased	-2,88	97
Hematological System Developn	selection of T lymphocytes	1,10E-12		-1,16	24
Hematological System Developn	selection of thymocytes	2,36E-12		-1,00	22
Hematological System Developn	cell movement of mononuclear leukocytes	1,04E-11		-0,75	93
Hematological System Developn	quantity of thymocytes	3,07E-11	Decreased	-2,10	59
Hematological System Developn	migration of mononuclear leukocytes	3,47E-11		-0,91	74
Hematological System Developn	quantity of B lymphocytes	6,32E-11	Decreased	-2,93	74
Hematological System Developn	function of CD4+ T-lymphocytes	7,99E-11			25
Hematological System Developn	cell movement of granulocytes	1,53E-10		-0,06	77
Hematological System Developn	proliferation of B lymphocytes	1,55E-10		-1,05	63
Hematological System Developn	cell movement of myeloid cells	1,79E-10		-0,15	98
Hematological System Developn	binding of lymphocytes	2,20E-10		-1,19	31
Hematological System Developn	binding of mononuclear leukocytes	2,89E-10		-0,76	35
Hematological System Developn	Lymphocyte migration	3,10E-10		-1,23	67
Hematological System Developn	cell movement of phagocytes	3,51E-10		0,16	98
Hematological System Developn	cell movement of lymphocytes	3,84E-10		-1,53	76
Hematological System Developn	quantity of myeloid cells	6,76E-10		-1,50	70
Hematological System Developn	function of myeloid cells	1,69E-09		-1,79	55
Hematological System Developn	binding of leukocytes	1,72E-09		-0,67	47
Hematological System Developn	lack of T lymphocytes	3,18E-09			15
Hematological System Developn	infiltration of leukocytes	7,55E-09		-0,49	70
Hematological System Developn	quantity of granulocytes	8,61E-09		-1,59	62
Hematological System Developn	function of helper T lymphocytes	2,35E-08			25
Hematological System Developn	chemotaxis of phagocytes	2,71E-08		1,23	57
Hematological System Developn	positive selection of T lymphocytes	3,03E-08		-0,89	12
Hematological System Developn	activation of phagocytes	3,55E-08		0,31	60
Hematological System Developn	activation of myeloid cells	4,09E-08		0,46	52
Hematological System Developn	homing of leukocytes	4,68E-08		0,47	69
Hematological System Developn	recruitment of granulocytes	4,96E-08		0,87	43
Hematological System Developn	cell movement of neutrophils	7,36E-08		0,84	58
Hematological System Developn	positive selection of thymocytes	8,15E-08		-0,96	11
Hematological System Developn	chemotaxis of neutrophils	2,40E-07		1,76	37
Hematological System Developn	recruitment of neutrophils	2,44E-07		1,54	36
Hematological System Developn	T cell migration	2,46E-07		-0,74	46
Hematological System Developn	development of thymocytes	2,71E-07		-1,23	23
Hematological System Developn	binding of T lymphocytes	2,79E-07		-1,58	22
Hematological System Developn	chemotaxis of granulocytes	3,23E-07		0,69	42
Hematological System Developn	function of granulocytes	5,11E-07			27
Hematological System Developn	generation of T lymphocytes	5,16E-07		-1,83	25
Hematological System Developn	recruitment of leukocytes	5,96E-07		-0,03	55
Hematological System Developn	quantity of phagocytes	6,14E-07	Decreased	-2,45	71
Hematological System Developn	chemotaxis of leukocytes	6,58E-07		0,82	63
Hematological System Developn	T cell response	8,57E-07		-0,23	28
Hematological System Developn	generation of lymphocytes	1,06E-06		-1,74	28
Hematological System Developn	interaction of T lymphocytes	1,07E-06	Decreased	-2,00	25
Hematological System Developn	interaction of leukocytes	1,17E-06		-1,77	26
Hematological System Developn	differentiation of helper T lymphocytes	1,18E-06		-0,13	36
Hematological System Developn	recruitment of phagocytes	1,26E-06		1,24	42
Hematological System Developn	infiltration of myeloid cells	1,68E-06		-0,37	42
Hematological System Developn	adhesion of immune cells	2,10E-06		0,14	63
Hematological System Developn	function of Th1 cells	2,25E-06			16
Hematological System Developn	development of hematopoietic cells	2,48E-06		-1,92	37
Hematological System Developn	activation of antigen presenting cells	2,51E-06		-0,63	45
Hematological System Developn	response of lymphocytes	2,75E-06		-0,24	32
Hematological System Developn	accumulation of leukocytes	2,86E-06		0,11	45
Hematological System Developn	cell movement of T lymphocytes	2,88E-06		-0,16	39
Hematological System Developn	growth of lymphocytes	2,89E-06		-1,67	37
Hematological System Developn	infiltration of granulocytes	3,12E-06		-0,15	38
Hematological System Developn	quantity of natural killer cells	3,22E-06	Decreased	-2,68	22
Hematological System Developn	negative selection of T lymphocytes	3,25E-06	Decreased	-2,06	9
Hematological System Developn	stimulation of leukocytes	3,30E-06		-0,83	35
Hematological System Developn	differentiation of hematopoietic cells	3,86E-06	Decreased	-2,01	48
Hematological System Developn	expansion of lymphocytes	3,87E-06	Decreased	-2,09	32
Hematological System Developn	stimulation of T lymphocytes	3,87E-06		-1,30	24
Hematological System Developn	accumulation of blood cells	4,26E-06		0,23	46
Hematological System Developn	migration of myeloid cells	4,35E-06		-1,19	33

Hematological System Developn	chemotaxis of myeloid cells	4,37E-06		0,97	50
Hematological System Developn	function of Th2 cells	5,96E-06			15
Hematological System Developn	proliferation of hematopoietic cells	6,10E-06		-1,12	40
Hematological System Developn	migration of phagocytes	6,18E-06		-0,70	49
Hematological System Developn	proliferation of hematopoietic progenitor cells	6,70E-06		-1,29	39
Hematological System Developn	migration of granulocytes	9,28E-06		-1,23	31
Hematological System Developn	generation of helper T lymphocytes	9,73E-06	Decreased	-2,37	13
Hematological System Developn	cell movement of antigen presenting cells	1,15E-05		-1,67	52
Hematological System Developn	arrest in differentiation of lymphocytes	1,23E-05			19
Hematological System Developn	adhesion of T lymphocytes	1,27E-05		0,68	22
Hematological System Developn	accumulation of myeloid cells	1,28E-05		0,02	31
Hematological System Developn	migration of neutrophils	1,35E-05		-0,16	25
Hematological System Developn	function of macrophages	1,53E-05			36
Hematological System Developn	expansion of T lymphocytes	1,56E-05	Decreased	-2,68	27
Hematological System Developn	differentiation of hematopoietic progenitor cell	1,56E-05	Decreased	-2,15	45
Hematological System Developn	development of hematopoietic progenitor cells	1,59E-05		-1,93	34
Hematological System Developn	stimulation of lymphocytes	1,60E-05		-1,20	26
Hematological System Developn	quantity of pre-T lymphocytes	1,77E-05	Increased	2,56	18
Hematological System Developn	function of neutrophils	1,86E-05			21
Hematological System Developn	development of helper T lymphocytes	2,10E-05		-0,99	16
Hematological System Developn	quantity of neutrophils	2,16E-05		-0,82	39
Hematological System Developn	growth of T lymphocytes	2,32E-05	Decreased	-2,41	29
Hematological System Developn	negative selection of thymocytes	2,55E-05		-1,79	7
Hematological System Developn	homing of lymphocytes	2,94E-05		-1,80	30
Hematological System Developn	abnormal morphology of cytotoxic T cells	3,84E-05			12
Hematological System Developn	differentiation of B lymphocytes	3,95E-05		-0,67	41
Hematological System Developn	differentiation of Th2 cells	3,98E-05		-0,22	19
Hematological System Developn	quantity of double-negative T lymphocyte	4,20E-05	Increased	2,52	17
Hematological System Developn	myelopoiesis	4,45E-05		-0,36	55
Hematological System Developn	quantity of eosinophils	6,06E-05		-1,32	23
Hematological System Developn	lack of CD4+ T-lymphocytes	7,07E-05			6
Hematological System Developn	cell movement of eosinophils	7,21E-05		-1,26	23
Hematological System Developn	adhesion of lymphocytes	8,15E-05		0,56	24
Hematological System Developn	quantity of memory T lymphocytes	8,76E-05		-1,11	15
Hematological System Developn	homing of mononuclear leukocytes	8,90E-05		-0,65	37
Hematological System Developn	arrest in differentiation of T lymphocytes	1,03E-04			11
Hematological System Developn	accumulation of phagocytes	1,05E-04		0,12	25
Hematological System Developn	lack of Single positive thymocytes	1,07E-04			4
Hematological System Developn	adhesion of mononuclear leukocytes	1,10E-04		0,32	28
Hematological System Developn	binding of B lymphocytes	1,19E-04		-0,65	8
Hematological System Developn	survival of lymphocytes	1,39E-04		-0,61	31
Hematological System Developn	generation of Th1 cells	1,62E-04		-1,93	6
Hematological System Developn	function of natural killer cells	1,62E-04			6
Hematological System Developn	abnormal morphology of double-negative T ly	1,95E-04			8
Hematological System Developn	accumulation of T lymphocytes	1,99E-04		-0,21	17
Hematological System Developn	influx of leukocytes	2,28E-04		0,18	15
Hematological System Developn	transmigration of granulocytes	2,35E-04		-1,09	13
Hematological System Developn	activation of macrophages	2,50E-04		0,00	29
Hematological System Developn	transmigration of leukocytes	2,61E-04		-1,36	23
Hematological System Developn	quantity of blood platelets	2,61E-04		-0,04	23
Hematological System Developn	differentiation of pre-B lymphocytes	2,73E-04		-1,32	10
Hematological System Developn	binding of granulocytes	2,84E-04		-0,28	15
Hematological System Developn	lymphopoiesis	2,84E-04		-0,06	15
Hematopoiesis	hematopoiesis	7,43E-25		-1,61	200
Hematopoiesis	development of mononuclear leukocytes	3,39E-24		-1,85	137
Hematopoiesis	development of leukocytes	3,97E-24		-1,82	142
Hematopoiesis	development of lymphocytes	1,33E-23		-1,82	135
Hematopoiesis	development of blood cells	6,16E-23		-1,97	152
Hematopoiesis	T cell development	3,16E-22		-1,67	123
Hematopoiesis	differentiation of mononuclear leukocytes	5,84E-22		-1,93	131
Hematopoiesis	differentiation of lymphocytes	9,70E-22		-1,87	123
Hematopoiesis	differentiation of leukocytes	1,50E-21		-1,69	148
Hematopoiesis	differentiation of T lymphocytes	1,56E-16		-1,07	89
Hematopoiesis	quantity of hematopoietic cells	7,26E-13	Decreased	-2,94	98
Hematopoiesis	quantity of hematopoietic progenitor cells	1,06E-12	Decreased	-2,88	97
Hematopoiesis	selection of T lymphocytes	1,10E-12		-1,16	24
Hematopoiesis	selection of thymocytes	2,36E-12		-1,00	22

Hematopoiesis	quantity of thymocytes	3,07E-11	Decreased	-2,10	59
Hematopoiesis	developmental process of thymocytes	4,02E-11		-1,91	45
Hematopoiesis	positive selection of lymphocytes	1,12E-08		-0,39	13
Hematopoiesis	positive selection of T lymphocytes	3,03E-08		-0,89	12
Hematopoiesis	positive selection of thymocytes	8,15E-08		-0,96	11
Hematopoiesis	development of thymocytes	2,71E-07		-1,23	23
Hematopoiesis	differentiation of helper T lymphocytes	1,18E-06		-0,13	36
Hematopoiesis	development of hematopoietic cells	2,48E-06		-1,92	37
Hematopoiesis	negative selection of T lymphocytes	3,25E-06	Decreased	-2,06	9
Hematopoiesis	differentiation of hematopoietic cells	3,86E-06	Decreased	-2,01	48
Hematopoiesis	proliferation of hematopoietic cells	6,10E-06		-1,12	40
Hematopoiesis	proliferation of hematopoietic progenitor cells	6,70E-06		-1,29	39
Hematopoiesis	arrest in differentiation of lymphocytes	1,23E-05			19
Hematopoiesis	differentiation of hematopoietic progenitor cell	1,56E-05	Decreased	-2,15	45
Hematopoiesis	development of hematopoietic progenitor cells	1,59E-05		-1,93	34
Hematopoiesis	quantity of pre-T lymphocytes	1,77E-05	Increased	2,56	18
Hematopoiesis	development of helper T lymphocytes	2,10E-05		-0,99	16
Hematopoiesis	negative selection of thymocytes	2,55E-05		-1,79	7
Hematopoiesis	differentiation of B lymphocytes	3,95E-05		-0,67	41
Hematopoiesis	differentiation of Th2 cells	3,98E-05		-0,22	19
Hematopoiesis	quantity of double-negative T lymphocyte	4,20E-05	Increased	2,52	17
Hematopoiesis	myelopoiesis	4,45E-05		-0,36	55
Hematopoiesis	arrest in differentiation of T lymphocytes	1,03E-04			11
Hematopoiesis	lack of Single positive thymocytes	1,07E-04			4
Hematopoiesis	developmental process of pre-B lymphocytes	1,63E-04		-1,50	17
Hematopoiesis	maturation of leukocytes	1,69E-04	Decreased	-2,25	38
Hematopoiesis	abnormal morphology of double-negative T ly	1,95E-04			8
Hematopoiesis	developmental process of pro-B lymphocytes	2,35E-04		-1,60	13
Hematopoiesis	morphology of hematopoietic progenitor cells	2,50E-04			29
Hematopoiesis	differentiation of pre-B lymphocytes	2,73E-04		-1,32	10
Hematopoiesis	lymphopoiesis	2,84E-04		-0,06	15
Hepatic System Disease	liver cancer	2,72E-04		0,49	69
Humoral Immune Response	quantity of immunoglobulin	2,48E-14		-1,75	74
Humoral Immune Response	production of antibody	7,58E-14		-1,85	76
Humoral Immune Response	quantity of IgG	1,28E-12		-1,47	59
Humoral Immune Response	quantity of B lymphocytes	6,32E-11	Decreased	-2,93	74
Humoral Immune Response	proliferation of B lymphocytes	1,55E-10		-1,05	63
Humoral Immune Response	quantity of IgG1	8,31E-09		-1,04	35
Humoral Immune Response	morphology of B lymphocytes	4,36E-08			24
Humoral Immune Response	quantity of IgG2a	6,43E-07		-0,66	27
Humoral Immune Response	quantity of IgE	7,39E-07		0,49	24
Humoral Immune Response	quantity of IgG3	5,19E-06		-0,71	22
Humoral Immune Response	abnormal morphology of B lymphocytes	6,26E-06			16
Humoral Immune Response	lack of B lymphocytes	2,93E-05			13
Humoral Immune Response	differentiation of B lymphocytes	3,95E-05		-0,67	41
Humoral Immune Response	quantity of IgM	3,98E-05	Decreased	-2,54	19
Humoral Immune Response	quantity of IgG2b	5,81E-05		-1,84	18
Humoral Immune Response	binding of B lymphocytes	1,19E-04		-0,65	8
Humoral Immune Response	morphology of germinal center	1,32E-04			17
Humoral Immune Response	abnormal morphology of germinal center	1,63E-04			14
Humoral Immune Response	differentiation of pre-B lymphocytes	2,73E-04		-1,32	10
Hypersensitivity Response	quantity of eosinophils	6,06E-05		-1,32	23
Hypersensitivity Response	cell movement of eosinophils	7,21E-05		-1,26	23
Immune Cell Trafficking	activation of leukocytes	2,94E-25		-1,92	154
Immune Cell Trafficking	activation of mononuclear leukocytes	4,29E-18	Decreased	-2,82	110
Immune Cell Trafficking	activation of lymphocytes	5,42E-17	Decreased	-2,56	104
Immune Cell Trafficking	activation of T lymphocytes	8,77E-17	Decreased	-2,37	87
Immune Cell Trafficking	cell movement of leukocytes	1,12E-13		-0,49	141
Immune Cell Trafficking	leukocyte migration	2,77E-13		-0,23	156
Immune Cell Trafficking	cell movement of mononuclear leukocytes	1,04E-11		-0,75	93
Immune Cell Trafficking	migration of mononuclear leukocytes	3,47E-11		-0,91	74
Immune Cell Trafficking	cell movement of granulocytes	1,53E-10		-0,06	77
Immune Cell Trafficking	cell movement of myeloid cells	1,79E-10		-0,15	98
Immune Cell Trafficking	Lymphocyte migration	3,10E-10		-1,23	67
Immune Cell Trafficking	cell movement of phagocytes	3,51E-10		0,16	98
Immune Cell Trafficking	cell movement of lymphocytes	3,84E-10		-1,53	76
Immune Cell Trafficking	infiltration of leukocytes	7,55E-09		-0,49	70

Immune Cell Trafficking	chemotaxis of phagocytes	2,71E-08		1,23	57
Immune Cell Trafficking	activation of phagocytes	3,55E-08		0,31	60
Immune Cell Trafficking	activation of myeloid cells	4,09E-08		0,46	52
Immune Cell Trafficking	homing of leukocytes	4,68E-08		0,47	69
Immune Cell Trafficking	recruitment of granulocytes	4,96E-08		0,87	43
Immune Cell Trafficking	cell movement of neutrophils	7,36E-08		0,84	58
Immune Cell Trafficking	chemotaxis of neutrophils	2,40E-07		1,76	37
Immune Cell Trafficking	recruitment of neutrophils	2,44E-07		1,54	36
Immune Cell Trafficking	T cell migration	2,46E-07		-0,74	46
Immune Cell Trafficking	chemotaxis of granulocytes	3,23E-07		0,69	42
Immune Cell Trafficking	recruitment of leukocytes	5,96E-07		-0,03	55
Immune Cell Trafficking	chemotaxis of leukocytes	6,58E-07		0,82	63
Immune Cell Trafficking	recruitment of phagocytes	1,26E-06		1,24	42
Immune Cell Trafficking	infiltration of myeloid cells	1,68E-06		-0,37	42
Immune Cell Trafficking	adhesion of immune cells	2,10E-06		0,14	63
Immune Cell Trafficking	activation of antigen presenting cells	2,51E-06		-0,63	45
Immune Cell Trafficking	accumulation of leukocytes	2,86E-06		0,11	45
Immune Cell Trafficking	cell movement of T lymphocytes	2,88E-06		-0,16	39
Immune Cell Trafficking	infiltration of granulocytes	3,12E-06		-0,15	38
Immune Cell Trafficking	migration of myeloid cells	4,35E-06		-1,19	33
Immune Cell Trafficking	chemotaxis of myeloid cells	4,37E-06		0,97	50
Immune Cell Trafficking	migration of phagocytes	6,18E-06		-0,70	49
Immune Cell Trafficking	migration of granulocytes	9,28E-06		-1,23	31
Immune Cell Trafficking	cell movement of antigen presenting cells	1,15E-05		-1,67	52
Immune Cell Trafficking	adhesion of T lymphocytes	1,27E-05		0,68	22
Immune Cell Trafficking	accumulation of myeloid cells	1,28E-05		0,02	31
Immune Cell Trafficking	migration of neutrophils	1,35E-05		-0,16	25
Immune Cell Trafficking	homing of lymphocytes	2,94E-05		-1,80	30
Immune Cell Trafficking	cell movement of eosinophils	7,21E-05		-1,26	23
Immune Cell Trafficking	adhesion of lymphocytes	8,15E-05		0,56	24
Immune Cell Trafficking	homing of mononuclear leukocytes	8,90E-05		-0,65	37
Immune Cell Trafficking	accumulation of phagocytes	1,05E-04		0,12	25
Immune Cell Trafficking	adhesion of mononuclear leukocytes	1,10E-04		0,32	28
Immune Cell Trafficking	accumulation of T lymphocytes	1,99E-04		-0,21	17
Immune Cell Trafficking	influx of leukocytes	2,28E-04		0,18	15
Immune Cell Trafficking	transmigration of granulocytes	2,35E-04		-1,09	13
Immune Cell Trafficking	activation of macrophages	2,50E-04		0,00	29
Immune Cell Trafficking	transmigration of leukocytes	2,61E-04		-1,36	23
Immune Cell Trafficking	binding of granulocytes	2,84E-04		-0,28	15
Immunological Disease	autoimmune disease	3,86E-21		-0,90	242
Immunological Disease	rheumatoid arthritis	6,20E-14		-1,89	150
Immunological Disease	hypersensitive reaction	2,65E-11		0,30	95
Immunological Disease	systemic lupus erythematosus	4,33E-09			38
Immunological Disease	immediate hypersensitivity	8,71E-09		0,91	71
Immunological Disease	hypoplasia of lymphoid organ	3,64E-08			39
Immunological Disease	eosinophilia	4,57E-08	Decreased	-2,40	31
Immunological Disease	atopic dermatitis	8,63E-08			59
Immunological Disease	lymphoproliferative disorder	1,07E-07		-0,65	47
Immunological Disease	delayed hypersensitive reaction	2,87E-07		-0,53	32
Immunological Disease	experimental autoimmune encephalomyelitis	5,88E-07		-0,25	46
Immunological Disease	lymphocytic leukemia	1,09E-06			42
Immunological Disease	juvenile rheumatoid arthritis	2,93E-06			29
Immunological Disease	B-cell leukemia	4,24E-06			29
Immunological Disease	polyarticular juvenile rheumatoid arthritis	1,08E-05			23
Immunological Disease	tumorigenesis of B-cell non-Hodgkin lymphon	1,17E-05		0,32	16
Immunological Disease	hypoplasia of spleen	1,74E-05			20
Immunological Disease	hypoplasia of thymus gland	2,40E-05			24
Immunological Disease	tumorigenesis of non-Hodgkin lymphoma	4,20E-05		-0,23	17
Immunological Disease	tumorigenesis of lymphoma	7,45E-05		-0,40	22
Immunological Disease	chronic B-cell leukemia	8,90E-05			22
Immunological Disease	tumorigenesis of thymus gland	1,07E-04		-1,00	4
Immunological Disease	autoimmune glomerulonephritis	3,06E-04		-1,15	8
Immunological Disease	membranous glomerulonephritis	3,25E-04			6
Infectious Disease	severe acute respiratory syndrome	9,83E-28			59
Infectious Disease	infection of respiratory tract	1,35E-24			61
Infectious Disease	infection of mammalia	3,17E-13		0,09	80
Infectious Disease	infection by bacteria	3,52E-09		-0,85	78

Infectious Disease	infection by virus	1,12E-08		1,08	203
Infectious Disease	parasitosis	7,49E-08	Increased	2,85	35
Infectious Disease	tuberculoid leprosy	1,93E-07			13
Infectious Disease	leprosy	2,38E-07			25
Inflammatory Disease	rheumatic disease	6,77E-22		0,03	224
Inflammatory Disease	arthritis	7,79E-18		0,25	199
Inflammatory Disease	rheumatoid arthritis	6,20E-14		-1,89	150
Inflammatory Disease	systemic lupus erythematosus	4,33E-09			38
Inflammatory Disease	colitis	1,13E-08		-0,90	45
Inflammatory Disease	dermatitis	4,45E-08		-0,25	76
Inflammatory Disease	atopic dermatitis	8,63E-08			59
Inflammatory Disease	encephalomyelitis	2,64E-07		-0,24	47
Inflammatory Disease	experimental autoimmune encephalomyelitis	5,88E-07		-0,25	46
Inflammatory Disease	encephalitis	7,40E-07		-0,36	48
Inflammatory Disease	ulcerative colitis	9,66E-07			20
Inflammatory Disease	juvenile rheumatoid arthritis	2,93E-06			29
Inflammatory Disease	inflammatory bowel disease	3,40E-06		-0,70	60
Inflammatory Disease	psoriatic arthritis	4,63E-06			21
Inflammatory Disease	airway hyperresponsiveness	9,04E-06		0,86	24
Inflammatory Disease	polyarticular juvenile rheumatoid arthritis	1,08E-05			23
Inflammatory Disease	myocarditis	1,79E-05		0,64	12
Inflammatory Disease	collagen-induced arthritis	8,90E-05		-0,87	22
Inflammatory Disease	acne	1,06E-04			22
Inflammatory Disease	experimentally-induced arthritis	1,45E-04		-0,41	28
Inflammatory Disease	glomerulonephritis	2,46E-04		-0,72	28
Inflammatory Disease	carditis	2,65E-04		0,20	14
Inflammatory Disease	autoimmune glomerulonephritis	3,06E-04		-1,15	8
Inflammatory Disease	membranous glomerulonephritis	3,25E-04			6
Lipid Metabolism	synthesis of eicosanoid	2,16E-05		-0,06	39
Lipid Metabolism	metabolism of eicosanoid	4,94E-05		0,10	41
Lipid Metabolism	synthesis of fatty acid	2,19E-04		-0,57	47
Lymphoid Tissue Structure and I	development of mononuclear leukocytes	3,39E-24		-1,85	137
Lymphoid Tissue Structure and I	development of leukocytes	3,97E-24		-1,82	142
Lymphoid Tissue Structure and I	development of lymphocytes	1,33E-23		-1,82	135
Lymphoid Tissue Structure and I	T cell development	3,16E-22		-1,67	123
Lymphoid Tissue Structure and I	differentiation of T lymphocytes	1,56E-16		-1,07	89
Lymphoid Tissue Structure and I	morphology of lymphatic system component	2,97E-13			94
Lymphoid Tissue Structure and I	selection of T lymphocytes	1,10E-12		-1,16	24
Lymphoid Tissue Structure and I	selection of thymocytes	2,36E-12		-1,00	22
Lymphoid Tissue Structure and I	morphology of lymphoid organ	3,28E-11			80
Lymphoid Tissue Structure and I	abnormal morphology of lymphoid organ	4,93E-11			77
Lymphoid Tissue Structure and I	morphology of thymus gland	7,77E-09			31
Lymphoid Tissue Structure and I	abnormal morphology of spleen	2,79E-08			55
Lymphoid Tissue Structure and I	positive selection of T lymphocytes	3,03E-08		-0,89	12
Lymphoid Tissue Structure and I	positive selection of thymocytes	8,15E-08		-0,96	11
Lymphoid Tissue Structure and I	development of lymphoid organ	2,71E-07	Decreased	-2,02	50
Lymphoid Tissue Structure and I	development of thymocytes	2,71E-07		-1,23	23
Lymphoid Tissue Structure and I	abnormal morphology of thymus gland	4,04E-07			27
Lymphoid Tissue Structure and I	differentiation of helper T lymphocytes	1,18E-06		-0,13	36
Lymphoid Tissue Structure and I	development of lymphatic system component	1,36E-06	Decreased	-2,28	64
Lymphoid Tissue Structure and I	negative selection of T lymphocytes	3,25E-06	Decreased	-2,06	9
Lymphoid Tissue Structure and I	quantity of lymphatic system cells	8,74E-06		-1,98	48
Lymphoid Tissue Structure and I	morphology of lymph node	1,62E-05			32
Lymphoid Tissue Structure and I	development of helper T lymphocytes	2,10E-05		-0,99	16
Lymphoid Tissue Structure and I	abnormal morphology of thymus cortex	2,38E-05			9
Lymphoid Tissue Structure and I	negative selection of thymocytes	2,55E-05		-1,79	7
Lymphoid Tissue Structure and I	differentiation of Th2 cells	3,98E-05		-0,22	19
Lymphoid Tissue Structure and I	proliferation of splenocytes	5,12E-05		-1,00	20
Lymphoid Tissue Structure and I	abnormal morphology of lymph node	5,74E-05			29
Lymphoid Tissue Structure and I	development of thymus gland	5,77E-05		-1,22	27
Lymphoid Tissue Structure and I	arrest in differentiation of T lymphocytes	1,03E-04			11
Lymphoid Tissue Structure and I	lack of Single positive thymocytes	1,07E-04			4
Lymphoid Tissue Structure and I	abnormal morphology of thymus medulla	1,08E-04			9
Lymphoid Tissue Structure and I	proliferation of lymphatic system cells	1,10E-04		-0,78	32
Lymphoid Tissue Structure and I	morphology of germinal center	1,32E-04			17
Lymphoid Tissue Structure and I	abnormal morphology of germinal center	1,63E-04			14
Molecular Transport	mobilization of Ca2+	6,22E-05		-0,85	59

Molecular Transport	quantity of glutathione	2,39E-04		-0,16	16
Neurological Disease	encephalomyelitis	2,64E-07		-0,24	47
Neurological Disease	experimental autoimmune encephalomyelitis	5,88E-07		-0,25	46
Neurological Disease	encephalitis	7,40E-07		-0,36	48
Neurological Disease	neuromuscular disease	1,58E-06		0,70	158
Neurological Disease	ischemic stroke	1,95E-06			15
Neurological Disease	brain ischemia	3,21E-06			16
Neurological Disease	encephalopathy	7,29E-06		0,19	192
Neurological Disease	cerebrovascular dysfunction	1,23E-05			19
Organ Development	development of lymphoid organ	2,71E-07	Decreased	-2,02	50
Organ Development	development of thymocytes	2,71E-07		-1,23	23
Organ Development	development of thymus gland	5,77E-05		-1,22	27
Organ Development	morphology of germinal center	1,32E-04			17
Organ Development	abnormal morphology of germinal center	1,63E-04			14
Organ Morphology	morphology of lymphoid organ	3,28E-11			80
Organ Morphology	abnormal morphology of lymphoid organ	4,93E-11			77
Organ Morphology	morphology of thymus gland	7,77E-09			31
Organ Morphology	abnormal morphology of spleen	2,79E-08			55
Organ Morphology	abnormal morphology of thymus gland	4,04E-07			27
Organ Morphology	morphology of lymph node	1,62E-05			32
Organ Morphology	abnormal morphology of lymph node	5,74E-05			29
Organ Morphology	lack of Single positive thymocytes	1,07E-04			4
Organ Morphology	involution of organ	1,19E-04			8
Organismal Development	development of lymphoid organ	2,71E-07	Decreased	-2,02	50
Organismal Development	development of thymocytes	2,71E-07		-1,23	23
Organismal Development	development of thymus gland	5,77E-05		-1,22	27
Organismal Development	morphology of germinal center	1,32E-04			17
Organismal Development	abnormal morphology of germinal center	1,63E-04			14
Organismal Injury and Abnormal	damage of connective tissue	2,24E-04		0,27	23
Organismal Survival	survival of organism	4,16E-07		-1,14	102
Organismal Survival	organismal death	1,11E-04		-1,49	219
Post-Translational Modification	phosphorylation of protein	5,46E-09		-0,51	122
Post-Translational Modification	tyrosine phosphorylation of protein	7,74E-09		-0,15	46
Post-Translational Modification	phosphorylation of L-amino acid	1,06E-05		-0,48	37
Post-Translational Modification	phosphorylation of L-tyrosine	8,33E-05		-0,38	28
Protein Synthesis	quantity of immunoglobulin	2,48E-14		-1,75	74
Protein Synthesis	production of antibody	7,58E-14		-1,85	76
Protein Synthesis	quantity of IgG	1,28E-12		-1,47	59
Protein Synthesis	quantity of IgG1	8,31E-09		-1,04	35
Protein Synthesis	quantity of IgG2a	6,43E-07		-0,66	27
Protein Synthesis	quantity of IgE	7,39E-07		0,49	24
Protein Synthesis	quantity of IgG3	5,19E-06		-0,71	22
Protein Synthesis	quantity of IgM	3,98E-05	Decreased	-2,54	19
Protein Synthesis	quantity of IgG2b	5,81E-05		-1,84	18
Protein Synthesis	quantity of interleukin	3,20E-04		-0,65	24
Renal and Urological Disease	renal cancer	1,31E-07			61
Renal and Urological Disease	urological disorder	8,51E-05		0,48	140
Renal and Urological Disease	glomerulonephritis	2,46E-04		-0,72	28
Renal and Urological Disease	autoimmune glomerulonephritis	3,06E-04		-1,15	8
Renal and Urological Disease	membranous glomerulonephritis	3,25E-04			6
Reproductive System Disease	genital tumor	1,94E-09		0,66	152
Reproductive System Disease	prostate cancer	3,81E-06		-0,19	84
Reproductive System Disease	prostatic tumor	4,56E-06		-0,32	85
Reproductive System Disease	gonadal tumor	9,15E-06		1,08	81
Reproductive System Disease	uterine serous papillary cancer	1,63E-05			50
Reproductive System Disease	endometrial cancer	3,46E-05			64
Reproductive System Disease	endometrial carcinoma	3,56E-05			68
Reproductive System Disease	endometriosis	8,76E-05			67
Reproductive System Disease	uterine cancer	8,84E-05			107
Reproductive System Disease	breast cancer	9,17E-05		0,57	133
Respiratory Disease	severe acute respiratory syndrome	9,83E-28			59
Respiratory Disease	infection of respiratory tract	1,35E-24			61
Respiratory Disease	lung adenocarcinoma	1,62E-06		-0,14	32
Respiratory Disease	airway hyperresponsiveness	9,04E-06		0,86	24
Respiratory Disease	pneumonitis	4,19E-05		-0,02	31
Skeletal and Muscular Disorders	rheumatic disease	6,77E-22		0,03	224
Skeletal and Muscular Disorders	arthritis	7,79E-18		0,25	199

Skeletal and Muscular Disorders	rheumatoid arthritis	6,20E-14		-1,89	150
Skeletal and Muscular Disorders	systemic lupus erythematosus	4,33E-09			38
Skeletal and Muscular Disorders	neuromuscular disease	1,58E-06		0,70	158
Skeletal and Muscular Disorders	ischemic stroke	1,95E-06			15
Skeletal and Muscular Disorders	juvenile rheumatoid arthritis	2,93E-06			29
Skeletal and Muscular Disorders	psoriatic arthritis	4,63E-06			21
Skeletal and Muscular Disorders	polyarticular juvenile rheumatoid arthritis	1,08E-05			23
Skeletal and Muscular Disorders	collagen-induced arthritis	8,90E-05		-0,87	22
Skeletal and Muscular Disorders	experimentally-induced arthritis	1,45E-04		-0,41	28
Skeletal and Muscular System D	mitosis of cardiomyocytes	1,09E-05		0,36	5
Small Molecule Biochemistry	phosphorylation of L-amino acid	1,06E-05		-0,48	37
Small Molecule Biochemistry	synthesis of eicosanoid	2,16E-05		-0,06	39
Small Molecule Biochemistry	metabolism of eicosanoid	4,94E-05		0,10	41
Small Molecule Biochemistry	phosphorylation of L-tyrosine	8,33E-05		-0,38	28
Small Molecule Biochemistry	synthesis of fatty acid	2,19E-04		-0,57	47
Small Molecule Biochemistry	quantity of glutathione	2,39E-04		-0,16	16
Tissue Development	adhesion of blood cells	5,16E-08		0,07	73
Tissue Development	accumulation of cells	2,68E-07		0,30	60
Tissue Development	development of lymphoid organ	2,71E-07	Decreased	-2,02	50
Tissue Development	development of thymocytes	2,71E-07		-1,23	23
Tissue Development	binding of T lymphocytes	2,79E-07		-1,58	22
Tissue Development	generation of T lymphocytes	5,16E-07		-1,83	25
Tissue Development	generation of lymphocytes	1,06E-06		-1,74	28
Tissue Development	interaction of T lymphocytes	1,07E-06	Decreased	-2,00	25
Tissue Development	interaction of leukocytes	1,17E-06		-1,77	26
Tissue Development	adhesion of immune cells	2,10E-06		0,14	63
Tissue Development	accumulation of leukocytes	2,86E-06		0,11	45
Tissue Development	accumulation of blood cells	4,26E-06		0,23	46
Tissue Development	generation of helper T lymphocytes	9,73E-06	Decreased	-2,37	13
Tissue Development	generation of leukocytes	1,02E-05	Decreased	-2,09	29
Tissue Development	adhesion of T lymphocytes	1,27E-05		0,68	22
Tissue Development	accumulation of myeloid cells	1,28E-05		0,02	31
Tissue Development	development of thymus gland	5,77E-05		-1,22	27
Tissue Development	adhesion of lymphocytes	8,15E-05		0,56	24
Tissue Development	accumulation of phagocytes	1,05E-04		0,12	25
Tissue Development	adhesion of mononuclear leukocytes	1,10E-04		0,32	28
Tissue Development	involution of organ	1,19E-04			8
Tissue Development	morphology of germinal center	1,32E-04			17
Tissue Development	generation of Th1 cells	1,62E-04		-1,93	6
Tissue Development	abnormal morphology of germinal center	1,63E-04			14
Tissue Development	accumulation of T lymphocytes	1,99E-04		-0,21	17
Tissue Development	binding of granulocytes	2,84E-04		-0,28	15
Tissue Morphology	quantity of leukocytes	8,73E-29	Decreased	-4,69	208
Tissue Morphology	quantity of blood cells	1,04E-27	Decreased	-4,76	225
Tissue Morphology	quantity of mononuclear leukocytes	6,47E-26	Decreased	-5,18	172
Tissue Morphology	quantity of lymphocytes	2,35E-24	Decreased	-4,80	164
Tissue Morphology	quantity of T lymphocytes	2,45E-21	Decreased	-4,33	127
Tissue Morphology	quantity of cells	1,10E-16	Decreased	-3,57	286
Tissue Morphology	quantity of helper T lymphocytes	3,75E-16	Decreased	-2,95	54
Tissue Morphology	quantity of cytotoxic T cells	9,67E-14		-0,56	43
Tissue Morphology	quantity of hematopoietic cells	7,26E-13	Decreased	-2,94	98
Tissue Morphology	quantity of hematopoietic progenitor cells	1,06E-12	Decreased	-2,88	97
Tissue Morphology	quantity of thymocytes	3,07E-11	Decreased	-2,10	59
Tissue Morphology	quantity of B lymphocytes	6,32E-11	Decreased	-2,93	74
Tissue Morphology	quantity of myeloid cells	6,76E-10		-1,50	70
Tissue Morphology	quantity of granulocytes	8,61E-09		-1,59	62
Tissue Morphology	quantity of phagocytes	6,14E-07	Decreased	-2,45	71
Tissue Morphology	quantity of natural killer cells	3,22E-06	Decreased	-2,68	22
Tissue Morphology	quantity of lymphatic system cells	8,74E-06		-1,98	48
Tissue Morphology	morphology of lymph node	1,62E-05			32
Tissue Morphology	quantity of pre-T lymphocytes	1,77E-05	Increased	2,56	18
Tissue Morphology	quantity of neutrophils	2,16E-05		-0,82	39
Tissue Morphology	quantity of double-negative T lymphocyte	4,20E-05	Increased	2,52	17
Tissue Morphology	abnormal morphology of lymph node	5,74E-05			29
Tissue Morphology	quantity of eosinophils	6,06E-05		-1,32	23
Tissue Morphology	quantity of memory T lymphocytes	8,76E-05		-1,11	15
Tissue Morphology	size of lesion	1,37E-04		-0,28	38

Tissue Morphology	quantity of blood platelets	2,61E-04	-0,04	23
Tumor Morphology	proliferation of tumor cells	1,19E-04	0,88	40
Vitamin and Mineral Metabolism	mobilization of Ca2+	6,22E-05	-0,85	59
