# Supplementary material for: Early and dynamic changes in gene expression in septic shock patients: a genome-wide approach
Source: Intensive Care Med Exp. 2014 Aug 20;2:20. doi: 10.1186/s40635-014-0020-3 (PMC4512996; doi:10.1186/s40635-014-0020-3)
Supplement: Additional file 5: Table S4. — Significant categories enriched by the 142 probe sets differentially expressed between. [file 40635_2014_20_MOESM5_ESM.pdf]

Table S3. Significant "cell death" functions in septic shock patients over time compared to Healthy volu

	H0					
	Low			High		
**Functions Annotation**	**p-Value**	**Predicted Activation State**	**Bias-corrected z-score**	**p-Value**	**Predicted Activation State**	**Bias-corrected z-score**
activation-induced cell death	7.71E-05		0.619	1.12E-04		-0.174
activation-induced cell death of lymphocytes						
activation-induced cell death of T lymphocytes						
apoptosis	3.90E-16		0.867	1.47E-16		-0.258
apoptosis of airway epithelial cells						
apoptosis of B lymphocytes	1.57E-05		1.298			
apoptosis of bladder cancer cell lines	3.42E-04		1.154			
apoptosis of blood cells	8.75E-17		1.289	1.60E-14		0.377
apoptosis of breast cancer cell lines						
apoptosis of epithelial cells						
apoptosis of colon cancer cell lines				1.75E-04		-0.702
apoptosis of epithelial cells	1.54E-04		-0.230			
apoptosis of granulocytes	2.80E-08		-0.072			
apoptosis of hematopoietic cells	1.05E-06		0.376			
apoptosis of hematopoietic progenitor cells	4.79E-07		0.262	6.32E-07		0.182
apoptosis of leukemia cell lines	2.65E-11		1.052	4.19E-07		1.149
apoptosis of leukocytes	2.64E-17		1.451	2.17E-14		0.564
apoptosis of lung cancer cell lines						
apoptosis of lung cell lines						
apoptosis of lymphoblasts	6.82E-05		0.825			
apoptosis of lymphocytes	9.41E-12		1.500	6.00E-09		0.541
apoptosis of lymphoid organ	2.37E-06		0.727	3.28E-05		0.661
apoptosis of lymphoma cell lines	6.20E-07	Increased	2.173	2.13E-05		1.952
apoptosis of macrophages	1.66E-04		1.836			
apoptosis of mononuclear leukocytes	3.48E-12		1.851	8.37E-10		0.778
apoptosis of muscle cells						
apoptosis of myeloid cells	3.08E-10		1.295	1.18E-06		0.459
apoptosis of neutrophils	3.53E-07		-1.252			
apoptosis of organ	2.53E-06		-0.027	2.66E-05		-0.497
apoptosis of peripheral blood leukocytes						
apoptosis of ovarian cancer cell lines						
apoptosis of phagocytes	5.02E-08		0.656	2.54E-05		-0.002
apoptosis of pre-B lymphocytes						
apoptosis of prostate cancer cell lines				1.47E-04		-0.124
apoptosis of T lymphocytes	7.56E-11		0.710	1.69E-08		0.062
apoptosis of thymocytes	7.04E-08		0.708	1.95E-06		0.684
apoptosis of tumor cell lines	7.24E-10		1.499	9.74E-10		0.088
cell death	3.68E-15		0.591	1.06E-20		-0.167
cell death of B lymphocytes	1.42E-05		1.159			
cell death of bladder cancer cell lines	7.69E-05		0.849			
cell death of blood cells	3.40E-20	Increased	2.336	5.54E-20		0.945
cell death of breast cancer cell lines						
cell death of bronchial epithelial cells						
cell death of cancer cells				8.04E-05		-1.944

cell death of colon cancer cell lines			0.741	1.96E-04		-0.718
cell death of epithelial cells						
cell death of granulocytes	1.61E-08		0.397			
cell death of connective tissue cells						
cell death of fibroblast cell lines						
cell death of hematopoietic cells	2.30E-05		0.284			
cell death of hematopoietic progenitor cells	1.32E-05		2.580	6.17E-06		-0.012
cell death of immune cells	3.56E-21	Increased	1.116	5.47E-20		1.134
cell death of leukemia cell lines	9.35E-13		1.809	4.93E-09		0.896
cell death of leukocyte cell lines	2.70E-04		2.155			
cell death of lung cancer cell lines						
cell death of lung cells						
cell death of lymphocytes	3.38E-13	Increased	0.553	6.15E-11		0.793
cell death of lymphoid organ	1.18E-05		1.169	4.50E-05		0.259
cell death of lymphoma cell lines	1.23E-08		2.639	3.91E-07		0.803
cell death of macrophages	2.43E-04	Increased	2.524			
cell death of lymphoma cells						
cell death of mononuclear leukocytes	1.24E-13	Increased	2.517	8.08E-12		1.052
cell death of muscle cells						
cell death of myeloid cells	5.48E-10	Increased	1.618	1.17E-06		1.267
cell death of organ	1.10E-04		1.720	7.29E-05		0.947
cell death of peripheral blood leukocytes						
cell death of phagocytes	9.13E-08		1.349	3.10E-05		0.662
cell death of prostate cancer cell lines				1.23E-04		-0.369
cell death of T lymphocytes	4.59E-12		0.686	7.46E-10		0.373
cell death of thymocytes	1.15E-06		1.044	6.55E-06		0.429
cell death of tumor cell lines	2.34E-09		0.063	5.46E-11		-0.362
cell death of tumor cells				5.27E-05		-1.820
cell survival	5.67E-06		1.060	1.71E-08		1.109
cell viability						
cell viability of B-lymphocyte derived cell lines						
cell viability of cervical cancer cell lines						
cell viability of tumor cell lines						
clearance of cells	5.72E-06		-0.385	4.48E-05		0.502
cytolysis	7.06E-09		-1.112	6.22E-08		-1.106
cytolysis of blood cells	3.30E-06			2.52E-05		-1.768
cytolysis of leukocyte cell lines				2.08E-04		0.982
cytolysis of leukocytes				1.16E-05		-1.731
cytolysis of lymphocytes	2.30E-06		-0.106	3.10E-05		
cytolysis of natural killer cells	1.84E-05		-0.076			
cytolysis of T lymphocytes				1.64E-04		
cytolysis of tumor cell lines	1.61E-06		-0.128	1.69E-04		-0.378
cytotoxicity	9.65E-13		-0.386	2.06E-13		-1.150
cytotoxicity of cells	4.23E-11		-0.902	1.15E-11		-1.246
cytotoxicity of leukocytes	2.95E-11		-0.541	1.37E-11		-1.552
cytotoxicity of lymphocytes	4.52E-10		-0.985	6.36E-11	Decreased	-2.111
cytotoxicity of natural killer cells	3.54E-09		0.175	3.93E-09		-1.959
cytotoxicity of T lymphocytes	3.56E-05		-0.816	1.68E-04		-1.189
inhibition of apoptosis						
killing of cells	2.21E-11		-1.130	1.88E-09		0.371
killing of hematopoietic cells	1.85E-04		-0.694	1.23E-04		-0.931
killing of leukocytes	1.96E-08		-0.583	2.26E-07		-1.610

killing of lymphocytes	8.94E-08	-1.103	1.62E-06	-1.092
killing of lymphoma cell lines				
killing of natural killer cells	2.02E-04		1.24E-04	-0.899
killing of splenocytes				
killing of T lymphocytes	3.55E-07	1.210	1.24E-04	-1.231
killing of tumor cell lines				
killing of tumor cells	2.54E-04	1.296		
pyroptosis of bone marrow-derived macrophages	1.48E-04	-0.034		
removal of cells	1.87E-06	0.003	1.33E-05	0.783
survival of B lymphocytes				
survival of blood cells	2.45E-05	-0.108	9.46E-05	0.251
survival of epithelial cells				
survival of leukocytes	3.68E-05	-0.250	3.42E-04	-0.193
survival of lymphocytes	2.60E-04	1.193		
survival of mononuclear leukocytes	2.76E-04			
toxicity of cells	1.48E-04			

nteers. The table shows the predicted activation or inhibition status of the enriched functions in SAPSII-Low

H24						H48				
Low			High			Low			High	
p-Value	Predicted Activation State	Bias-corrected z-score	p-Value	Predicted Activation State	Bias-corrected z-score	p-Value	Predicted Activation State	Bias-corrected z-score	p-Value	Predicted Activation State
4.89E-05		0.628	6.08E-05		-0.238	4.69E-06		0.117	1.71E-05	
2.37E-04		0.399	2.80E-04		0.325	7.76E-06		0.038	1.10E-04	
						6.09E-05		0.008		
4.79E-16		1.367	9.92E-15		0.857	1.98E-14		1.931	6.16E-16	
7.90E-05		-0.821				2.47E-05		-0.849		
2.60E-06		-0.735	5.47E-05		0.398	5.24E-06		-0.871	2.53E-04	
8.10E-16		0.740	3.62E-16		1.533	1.15E-19		0.497	1.23E-15	
2.37E-05		1.211	3.38E-04		-0.703				3.26E-04	
						1.72E-05		-0.953	1.07E-04	
8.72E-07		-0.162								
9.13E-06		-0.050	2.06E-04		0.455	5.75E-07		-0.067	1.82E-04	
1.78E-05		0.314	2.81E-06		0.604	1.00E-07		-0.081	1.67E-06	
1.18E-05		0.175	7.59E-06		0.567	6.61E-08		-0.251	2.08E-06	
1.66E-13	Increased	2.062	9.56E-09		1.155	3.13E-12		1.739	4.03E-12	
2.83E-17		0.721	2.27E-16		1.596	4.25E-21		0.429	1.25E-15	
3.53E-04		1.002								
1.13E-04		-0.197				1.58E-04		-0.927		
1.37E-04		0.547	1.13E-04		0.988				6.00E-05	
1.52E-12		0.148	6.88E-12		0.830	2.44E-15		-0.338	1.90E-10	
7.66E-05		0.801	2.89E-05		0.691	2.78E-07		0.530	7.86E-05	
1.03E-04		1.255	2.24E-05		1.821	1.17E-05		1.695	2.16E-05	
3.60E-13		0.481	2.91E-12		1.191	3.84E-16		0.072	2.65E-11	
1.79E-04		-0.375				6.10E-05		-0.811		
5.57E-08		1.497	8.49E-08		1.962	2.21E-08		1.489	1.73E-07	
1.40E-04		-0.907				1.96E-05		-0.886		
3.15E-07		0.149	1.45E-05		-0.502	1.40E-08		0.163	5.48E-06	
						1.15E-04		1.002		
4.23E-04		0.326								
4.76E-06		1.197	6.38E-06		1.707	2.31E-06		1.164	2.86E-05	
						2.61E-04		-1.904		
1.05E-11		0.060	7.56E-11		0.083	1.75E-14		-0.364	1.00E-10	
7.79E-06		0.865	1.18E-06		0.713	1.71E-08		0.418	4.99E-06	
1.46E-10		1.839	2.51E-10		1.827	3.35E-09		1.668	7.04E-13	
1.59E-14		0.713	1.17E-16		0.172	8.45E-14		0.886	9.64E-18	
1.71E-06		-0.676	5.25E-05		0.279	2.55E-06		-0.776	2.02E-04	
3.89E-20		1.188	3.30E-21		1.661	1.94E-23		0.859	1.05E-19	
2.40E-04		1.101								
2.33E-04		-0.007				9.88E-05		-0.049		

2.46E-04		0.747								
			1.28E-04		0.900					
									3.87E-04	
									3.92E-04	
1.63E-04		0.527	5.88E-06		0.696	1.33E-06		0.110	2.79E-06	
1.24E-04		0.392	1.54E-05		0.660	1.02E-06		-0.053	3.58E-06	
1.07E-21		1.191	6.49E-22		1.787	5.99E-25		0.805	8.65E-20	
1.33E-15		1.786	9.57E-12		0.958	1.16E-13		1.595	4.00E-14	
2.60E-04		0.847								
2.84E-04		0.401								
3.14E-14		1.039	1.54E-13		1.355	4.72E-17		0.650	8.94E-13	
3.94E-04		0.724	2.34E-05		0.509	2.13E-06		0.441	4.81E-05	
8.06E-06		0.107	1.03E-07		0.352	1.31E-06		0.538	1.31E-06	
						1.76E-04		0.452		
2.42E-15		1.339	6.46E-14		1.780	2.06E-18		1.012	1.18E-13	
1.31E-04		-0.076				8.88E-05		-0.466		
4.21E-08	Increased	2.520	4.90E-08	Increased	2.700	1.10E-08	Increased	2.494	7.57E-08	Increased
3.34E-05		1.258	3.68E-04		0.879	5.89E-05		0.967	4.28E-04	
						7.12E-05		1.186		
4.82E-06	Increased	2.035	4.32E-06	Increased	2.348	1.81E-06		1.979	1.61E-05	
5.03E-13		0.904	3.14E-13		0.677	1.09E-15		0.570	2.24E-13	
			2.06E-06		0.681				6.24E-06	
5.18E-10		0.812	1.74E-10		0.993	4.34E-09		0.582	5.87E-12	
						1.39E-04		-1.260		
6.90E-07		-0.373	1.94E-07		0.330	4.47E-08		-0.497	7.55E-08	
1.96E-04		1.260				4.63E-05		0.999		
						2.09E-04		-0.661		
						1.76E-04		-1.303		
1.23E-04		1.024								
2.65E-05		1.788	3.54E-06		0.899	1.55E-05		0.540		
2.98E-11		-0.014	2.54E-08		-1.606	1.82E-13		-0.004	1.15E-08	
9.27E-08		-0.915	1.55E-06	Decreased	-2.089	7.50E-09		-0.746	7.25E-07	
			8.68E-07		-1.776				2.13E-06	
1.34E-07			2.39E-06			2.17E-08		-0.406	5.91E-06	
2.05E-05			4.56E-05			1.21E-05				
3.00E-05						2.50E-05		-0.333	2.60E-04	
3.78E-05		-0.421	1.18E-04		-1.104	6.91E-06		-0.582	4.98E-05	
3.31E-14		-0.666	1.71E-13		-1.173	5.78E-17		-1.437	4.46E-13	
8.40E-13		-0.567	6.66E-12		-1.251	1.29E-16		-1.376	6.40E-12	
4.58E-12		-0.775	5.84E-12		-1.397	6.82E-17		-1.241	4.07E-12	Decreased
3.12E-11		-1.212	1.97E-11		-1.885	7.34E-16		-1.626	4.08E-12	Decreased
1.33E-08		-0.711	8.45E-10		-1.617	8.52E-12		-1.103	2.36E-09	
1.15E-06		-1.269	2.91E-05		-1.109	1.14E-08		-1.537	7.57E-06	
						6.69E-05				
5.02E-11		-0.395	2.38E-10		-0.343	3.40E-14		-0.684	2.16E-09	
			2.81E-05		-1.226				1.65E-04	
7.02E-05		-1.339	3.60E-07		-1.741	1.74E-06		-1.835	4.64E-07	

2.33E-04		-1.053	2.49E-07		-0.982	7.72E-06		-1.544	4.72E-07
									5.10E-04
			7.01E-06		-0.756				2.50E-05
2.77E-04	Decreased	-2.001				1.37E-04	Decreased	-2.289	
			3.46E-04		-1.300				1.77E-04
3.01E-04		-0.756				5.44E-05		-0.882	
			5.54E-06		1.160				
			2.25E-04		0.730				
1.94E-05		-1.154	3.59E-05		-0.463	1.95E-07		-1.588	5.20E-04
9.97E-05		0.887							
9.10E-06		-1.192	9.98E-06		-0.595	7.71E-07		-1.618	2.22E-04
3.53E-04		-1.663				1.27E-04		-1.863	
3.20E-04		-1.814	2.13E-04		-0.957	3.89E-05	Decreased	-2.197	

and High p

**Bias-corrected z-score**
-0.276
0.279
1.161
0.150
0.888
-0.502
-0.695
0.066
0.587
0.658
1.361
0.909
0.930
0.558
0.948
1.441
0.837
1.079
-0.179
0.860
-0.273
0.788
1.911
0.429
0.145
1.510

-1.165
-1.361
0.667
0.735
1.565
1.140
1.553
0.730
0.193
1.853
2.141
0.795
1.823
0.715
0.733
1.189
-0.259
-1.506
-1.172
-1.153
-1.176
-1.783
-1.747
-2.212
-2.515
-1.962
-1.877
0.340
-1.138
-1.386

-0.945
-0.878
-0.653

-1.431

-0.643

-0.683
