# Supplementary material for: Early and dynamic changes in gene expression in septic shock patients: a genome-wide approach
Source: Intensive Care Med Exp. 2014 Aug 20;2:20. doi: 10.1186/s40635-014-0020-3 (PMC4512996; doi:10.1186/s40635-014-0020-3)
Supplement: Additional file 6: Figure S2. — Comparison of gene expression patterns of the 142 probe sets differentially expressed (FDR <0.05) between both groups of septic shock patients. Expressions at 0, 24, and 48 h after septic shock of genes in each of the 14 clusters from hierarchical clustering in Figure 6. [file 40635_2014_20_MOESM6_ESM.pdf]

Table S4. Significant categories enriched by the 142 probesets differentially expressed between

© 2000-2012 Ingenuity Systems, Inc. All rights reserved.

Category	Functions Annotation	p-Value
Cancer	Cancer	4.54E-14
Infectious Disease	Viral Infection	6.02E-13
Infectious Disease	infection of mammalia	1.84E-12
Cell Morphology	morphology of T lymphocytes	2.55E-11
Hematological System Development and Function	morphology of T lymphocytes	2.55E-11
Cell Morphology	morphology of lymphocytes	3.25E-11
Dermatological Diseases and Conditions	psoriasis	3.96E-11
Hematological System Development and Function	activation of blood cells	3.29E-10
Cell-To-Cell Signaling and Interaction	activation of blood cells	3.29E-10
Immunological Disease	systemic autoimmune syndrome	3.52E-10
Cell Death and Survival	cell death of immune cells	1.46E-09
Immunological Disease	experimental autoimmune encephalomyelitis	1.81E-09
Inflammatory Disease	experimental autoimmune encephalomyelitis	1.81E-09
Neurological Disease	experimental autoimmune encephalomyelitis	1.81E-09
Cancer	epithelial tumor	1.93E-09
Cellular Function and Maintenance	cellular homeostasis	1.94E-09
Cell Morphology	morphology of leukocytes	2.22E-09
Cancer	carcinoma	2.53E-09
Cell Morphology	abnormal morphology of T lymphocytes	2.67E-09
Hematological System Development and Function	abnormal morphology of T lymphocytes	2.67E-09
Hematological System Development and Function	activation of leukocytes	2.91E-09
Cell-To-Cell Signaling and Interaction	activation of leukocytes	2.91E-09
Immune Cell Trafficking	activation of leukocytes	2.91E-09
Inflammatory Response	activation of leukocytes	2.91E-09
Immunological Disease	rheumatoid arthritis	3.08E-09
Inflammatory Disease	rheumatoid arthritis	3.08E-09
Connective Tissue Disorders	rheumatoid arthritis	3.08E-09
Skeletal and Muscular Disorders	rheumatoid arthritis	3.08E-09
Cancer	solid tumor	3.31E-09
Cell Morphology	abnormal morphology of leukocytes	3.88E-09
Cell Morphology	morphology of blood cells	5.75E-09
Inflammatory Disease	arthritis	6.67E-09
Connective Tissue Disorders	arthritis	6.67E-09
Skeletal and Muscular Disorders	arthritis	6.67E-09
Cellular Function and Maintenance	function of leukocytes	7.83E-09
Cell-To-Cell Signaling and Interaction	activation of cells	1.01E-08
Inflammatory Disease	Inflammatory Bowel Disease	1.36E-08
Gastrointestinal Disease	Inflammatory Bowel Disease	1.36E-08
Neurological Disease	neuromuscular disease	1.59E-08
Skeletal and Muscular Disorders	neuromuscular disease	1.59E-08
Cell Morphology	abnormal morphology of lymphocytes	1.66E-08
Inflammatory Disease	Rheumatic Disease	1.72E-08
Connective Tissue Disorders	Rheumatic Disease	1.72E-08
Skeletal and Muscular Disorders	Rheumatic Disease	1.72E-08
Hematological System Development and Function	development of blood cells	3.29E-08
Cellular Development	development of blood cells	3.29E-08
Hematopoiesis	development of blood cells	3.29E-08
Dermatological Diseases and Conditions	lichen planus	6.25E-08

Immunological Disease	lichen planus	6.25E-08
Inflammatory Disease	lichen planus	6.25E-08
Hematological System Development and Function	development of lymphocytes	6.57E-08
Cellular Development	development of lymphocytes	6.57E-08
Hematopoiesis	development of lymphocytes	6.57E-08
Lymphoid Tissue Structure and Development	development of lymphocytes	6.57E-08
Hematological System Development and Function	T cell development	9.04E-08
Cellular Function and Maintenance	T cell development	9.04E-08
Cellular Development	T cell development	9.04E-08
Hematopoiesis	T cell development	9.04E-08
Lymphoid Tissue Structure and Development	T cell development	9.04E-08
Cell-mediated Immune Response	T cell development	9.04E-08
Immunological Disease	hypersensitive reaction	1.10E-07
Inflammatory Response	inflammation of organ	1.13E-07
Cellular Development	differentiation of cells	1.29E-07
Gastrointestinal Disease	diabetes mellitus	1.69E-07
Endocrine System Disorders	diabetes mellitus	1.69E-07
Metabolic Disease	diabetes mellitus	1.69E-07
Cell Death and Survival	apoptosis of leukocytes	2.07E-07
Hematological System Development and Function	quantity of blood cells	2.31E-07
Tissue Morphology	quantity of blood cells	2.31E-07
Cell Death and Survival	cell death	2.71E-07
Cancer	hematological neoplasia	2.96E-07
Hematological Disease	hematological neoplasia	2.96E-07
Cell Death and Survival	apoptosis	3.03E-07
Cellular Development	proliferation of blood cells	3.68E-07
Cellular Growth and Proliferation	proliferation of blood cells	3.68E-07
Hematological System Development and Function	quantity of leukocytes	4.10E-07
Tissue Morphology	quantity of leukocytes	4.10E-07
Hematological System Development and Function	quantity of cytotoxic T cells	4.20E-07
Tissue Morphology	quantity of cytotoxic T cells	4.20E-07
Hematological System Development and Function	proliferation of lymphocytes	4.23E-07
Cellular Development	proliferation of lymphocytes	4.23E-07
Cellular Growth and Proliferation	proliferation of lymphocytes	4.23E-07
Hematological System Development and Function	proliferation of immune cells	5.56E-07
Cellular Development	proliferation of immune cells	5.56E-07
Cellular Growth and Proliferation	proliferation of immune cells	5.56E-07
Cellular Growth and Proliferation	proliferation of cells	5.91E-07
Cancer	renal cancer	6.57E-07
Renal and Urological Disease	renal cancer	6.57E-07
Hematological System Development and Function	activation of antigen presenting cells	6.88E-07
Cell-To-Cell Signaling and Interaction	activation of antigen presenting cells	6.88E-07
Immune Cell Trafficking	activation of antigen presenting cells	6.88E-07
Inflammatory Response	activation of antigen presenting cells	6.88E-07
Inflammatory Disease	multiple sclerosis	6.93E-07
Neurological Disease	multiple sclerosis	6.93E-07
Skeletal and Muscular Disorders	multiple sclerosis	6.93E-07
Neurological Disease	progressive motor neuropathy	7.11E-07
Hematological System Development and Function	differentiation of blood cells	7.50E-07
Cellular Development	differentiation of blood cells	7.50E-07
Cellular Movement	cell movement	7.95E-07
Metabolic Disease	glucose metabolism disorder	8.63E-07

Cell Death and Survival	cell viability	1.25E-06
Cell Death and Survival	cell survival	1.65E-06
Cellular Function and Maintenance	function of phagocytes	1.81E-06
Cellular Movement	migration of cells	1.83E-06
Cancer	genital tumor	1.96E-06
Reproductive System Disease	genital tumor	1.96E-06
Immunological Disease	insulin-dependent diabetes mellitus	1.97E-06
Gastrointestinal Disease	insulin-dependent diabetes mellitus	1.97E-06
Endocrine System Disorders	insulin-dependent diabetes mellitus	1.97E-06
Metabolic Disease	insulin-dependent diabetes mellitus	1.97E-06
Cancer	lymphohematopoietic cancer	2.06E-06
Cell Death and Survival	necrosis	2.08E-06
Hematological System Development and Function	differentiation of mononuclear leukocytes	2.10E-06
Cellular Development	differentiation of mononuclear leukocytes	2.10E-06
Hematopoiesis	differentiation of mononuclear leukocytes	2.10E-06
Immunological Disease	immediate hypersensitivity	2.59E-06
Inflammatory Response	antiviral response	2.65E-06
Antimicrobial Response	antiviral response	2.65E-06
Inflammatory Disease	colitis	2.70E-06
Inflammatory Response	colitis	2.70E-06
Gastrointestinal Disease	colitis	2.70E-06
Dermatological Diseases and Conditions	Dermatitis	2.88E-06
Inflammatory Disease	Dermatitis	2.88E-06
Cell Death and Survival	apoptosis of mononuclear leukocytes	2.94E-06
Hematological System Development and Function	differentiation of lymphocytes	2.94E-06
Cellular Development	differentiation of lymphocytes	2.94E-06
Hematopoiesis	differentiation of lymphocytes	2.94E-06
Hematological System Development and Function	cell movement of leukocytes	3.08E-06
Immune Cell Trafficking	cell movement of leukocytes	3.08E-06
Cellular Movement	cell movement of leukocytes	3.08E-06
Cellular Movement	infiltration of cells	3.61E-06
Cell Morphology	morphology of cells	4.23E-06
Infectious Disease	Bacterial Infection	5.29E-06
Cancer	hematologic cancer	6.07E-06
Hematological Disease	hematologic cancer	6.07E-06
Cell Death and Survival	cytotoxicity of cells	6.56E-06
Cancer	uterine cancer	6.88E-06
Reproductive System Disease	uterine cancer	6.88E-06
Hematological System Development and Function	quantity of T lymphocytes	7.61E-06
Tissue Morphology	quantity of T lymphocytes	7.61E-06
Hematological System Development and Function	inhibition of leukocytes	7.80E-06
Cellular Growth and Proliferation	inhibition of leukocytes	7.80E-06
Cancer	gastrointestinal tract cancer	7.98E-06
Gastrointestinal Disease	gastrointestinal tract cancer	7.98E-06
Cancer	uterine serous papillary cancer	9.89E-06
Reproductive System Disease	uterine serous papillary cancer	9.89E-06
Inflammatory Response	immune response of cells	1.01E-05
Cell Death and Survival	apoptosis of lymphocytes	1.01E-05
Dermatological Diseases and Conditions	atopic dermatitis	1.05E-05
Immunological Disease	atopic dermatitis	1.05E-05
Inflammatory Disease	atopic dermatitis	1.05E-05
Cellular Function and Maintenance	function of antigen presenting cells	1.15E-05

Inflammatory Response	inflammatory response	1.42E-05
Tissue Morphology	quantity of cells	1.57E-05
Cell Death and Survival	cell death of T lymphocytes	1.69E-05
Hematological System Development and Function	quantity of lymphocytes	1.77E-05
Tissue Morphology	quantity of lymphocytes	1.77E-05
Cellular Growth and Proliferation	generation of leukocytes	1.86E-05
Tissue Development	generation of leukocytes	1.86E-05
Cellular Movement	transmigration of cells	2.18E-05
Hematological System Development and Function	cell movement of mononuclear leukocytes	2.39E-05
Immune Cell Trafficking	cell movement of mononuclear leukocytes	2.39E-05
Cellular Movement	cell movement of mononuclear leukocytes	2.39E-05
Hematological System Development and Function	inhibition of mononuclear leukocytes	2.58E-05
Cellular Growth and Proliferation	inhibition of mononuclear leukocytes	2.58E-05
Cell-To-Cell Signaling and Interaction	detachment of corneocytes	2.59E-05
Tissue Development	detachment of corneocytes	2.59E-05
Cell Cycle	polyploidy of bone marrow cell lines	2.59E-05
Cell Death and Survival	apoptosis of T lymphocytes	2.66E-05
Cellular Function and Maintenance	function of lymphatic system cells	2.67E-05
Hematological System Development and Function	cell movement of myeloid cells	2.80E-05
Immune Cell Trafficking	cell movement of myeloid cells	2.80E-05
Cellular Movement	cell movement of myeloid cells	2.80E-05
Hematological System Development and Function	proliferation of T lymphocytes	3.12E-05
Cellular Development	proliferation of T lymphocytes	3.12E-05
Cellular Growth and Proliferation	proliferation of T lymphocytes	3.12E-05
Cellular Development	maturation of leukocytes	3.37E-05
Hematopoiesis	maturation of leukocytes	3.37E-05
Infectious Disease	replication of RNA virus	3.51E-05
Infectious Disease	severe acute respiratory syndrome	3.56E-05
Respiratory Disease	severe acute respiratory syndrome	3.56E-05
Hematological System Development and Function	quantity of intraepithelial T lymphocytes	3.58E-05
Tissue Morphology	quantity of intraepithelial T lymphocytes	3.58E-05
Hematological System Development and Function	function of lymphocytes	3.93E-05
Cellular Function and Maintenance	function of lymphocytes	3.93E-05
Cell Death and Survival	cytolysis	4.10E-05
Cancer	prostate cancer	4.40E-05
Reproductive System Disease	prostate cancer	4.40E-05
Hematological System Development and Function	proliferation of pre-B lymphocytes	5.67E-05
Cellular Development	proliferation of pre-B lymphocytes	5.67E-05
Hematopoiesis	proliferation of pre-B lymphocytes	5.67E-05
Cellular Growth and Proliferation	proliferation of pre-B lymphocytes	5.67E-05
Humoral Immune Response	proliferation of pre-B lymphocytes	5.67E-05
Hematological System Development and Function	activation of lymphocytes	7.01E-05
Cell-To-Cell Signaling and Interaction	activation of lymphocytes	7.01E-05
Immune Cell Trafficking	activation of lymphocytes	7.01E-05
Inflammatory Response	activation of lymphocytes	7.01E-05
Dermatological Diseases and Conditions	plaque psoriasis	7.16E-05
Hematological System Development and Function	quantity of helper T lymphocytes	7.46E-05
Tissue Morphology	quantity of helper T lymphocytes	7.46E-05
Cell-To-Cell Signaling and Interaction	antiviral response of fibroblasts	7.74E-05
Inflammatory Response	antiviral response of fibroblasts	7.74E-05
Antimicrobial Response	antiviral response of fibroblasts	7.74E-05
Connective Tissue Development and Function	antiviral response of fibroblasts	7.74E-05

Infectious Disease	infection by Newcastle disease virus	7.74E-05
Cancer	digestive organ tumor	8.00E-05
Gastrointestinal Disease	digestive organ tumor	8.00E-05
Hematological System Development and Function	chemotaxis of neutrophils	8.98E-05
Immune Cell Trafficking	chemotaxis of neutrophils	8.98E-05
Inflammatory Response	chemotaxis of neutrophils	8.98E-05
Cellular Movement	chemotaxis of neutrophils	8.98E-05
Cellular Movement	chemotaxis of cells	9.29E-05
Hematological System Development and Function	differentiation of T lymphocytes	9.37E-05
Cellular Function and Maintenance	differentiation of T lymphocytes	9.37E-05
Cellular Development	differentiation of T lymphocytes	9.37E-05
Hematopoiesis	differentiation of T lymphocytes	9.37E-05
Lymphoid Tissue Structure and Development	differentiation of T lymphocytes	9.37E-05
Cell-mediated Immune Response	differentiation of T lymphocytes	9.37E-05
Immunological Disease	delayed hypersensitive reaction	9.83E-05
Hematological System Development and Function	activation of phagocytes	9.91E-05
Cell-To-Cell Signaling and Interaction	activation of phagocytes	9.91E-05
Immune Cell Trafficking	activation of phagocytes	9.91E-05
Inflammatory Response	activation of phagocytes	9.91E-05
Infectious Disease	infection of leukocytes	9.94E-05
Immunological Disease	infection of leukocytes	9.94E-05
Hematological Disease	infection of leukocytes	9.94E-05
Infectious Disease	replication of Influenza A virus	1.05E-04
Inflammatory Disease	relapsing-remitting multiple sclerosis	1.06E-04
Neurological Disease	relapsing-remitting multiple sclerosis	1.06E-04
Skeletal and Muscular Disorders	relapsing-remitting multiple sclerosis	1.06E-04
Inflammatory Response	function of immune system	1.10E-04
Neurological Disease	disorder of basal ganglia	1.11E-04
Cancer	colorectal cancer	1.11E-04
Gastrointestinal Disease	colorectal cancer	1.11E-04
Hematological System Development and Function	cell movement of phagocytes	1.13E-04
Immune Cell Trafficking	cell movement of phagocytes	1.13E-04
Inflammatory Response	cell movement of phagocytes	1.13E-04
Cellular Movement	cell movement of phagocytes	1.13E-04
Hematological System Development and Function	proliferation of B lymphocytes	1.14E-04
Cellular Development	proliferation of B lymphocytes	1.14E-04
Cellular Growth and Proliferation	proliferation of B lymphocytes	1.14E-04
Humoral Immune Response	proliferation of B lymphocytes	1.14E-04
Inflammatory Disease	primary biliary cirrhosis	1.22E-04
Gastrointestinal Disease	primary biliary cirrhosis	1.22E-04
Hepatic System Disease	primary biliary cirrhosis	1.22E-04
Liver Cirrhosis	primary biliary cirrhosis	1.22E-04
Organismal Injury and Abnormalities	primary biliary cirrhosis	1.22E-04
Hematological System Development and Function	activation of T lymphocytes	1.28E-04
Cell-To-Cell Signaling and Interaction	activation of T lymphocytes	1.28E-04
Immune Cell Trafficking	activation of T lymphocytes	1.28E-04
Inflammatory Response	activation of T lymphocytes	1.28E-04
Cell Death and Survival	cell viability of mononuclear leukocytes	1.28E-04
Inflammatory Disease	Pancreatitis	1.28E-04
Gastrointestinal Disease	Pancreatitis	1.28E-04
Endocrine System Disorders	Pancreatitis	1.28E-04
Inflammatory Response	inflammation of joint	1.34E-04

Cell Morphology	abnormal morphology of B-1 lymphocytes	1.54E-04
Humoral Immune Response	abnormal morphology of B-1 lymphocytes	1.54E-04
Tissue Development	angiogenesis of adductor muscle	1.54E-04
Cardiovascular System Development and Function	angiogenesis of adductor muscle	1.54E-04
Embryonic Development	angiogenesis of adductor muscle	1.54E-04
Organ Development	angiogenesis of adductor muscle	1.54E-04
Organismal Development	angiogenesis of adductor muscle	1.54E-04
Skeletal and Muscular System Development and Functi	angiogenesis of adductor muscle	1.54E-04
Gastrointestinal Disease	damage of crypt	1.54E-04
Inflammatory Response	degranulation of granulocytes	1.56E-04
Cellular Compromise	degranulation of granulocytes	1.56E-04
Hematological System Development and Function	migration of mononuclear leukocytes	1.60E-04
Immune Cell Trafficking	migration of mononuclear leukocytes	1.60E-04
Cellular Movement	migration of mononuclear leukocytes	1.60E-04
Cell Death and Survival	apoptosis of antigen presenting cells	1.60E-04
Cell Cycle	mitogenesis of lymphocytes	1.62E-04
Hematological System Development and Function	chemotaxis of myeloid cells	1.84E-04
Immune Cell Trafficking	chemotaxis of myeloid cells	1.84E-04
Inflammatory Response	chemotaxis of myeloid cells	1.84E-04
Cellular Movement	chemotaxis of myeloid cells	1.84E-04
Lymphoid Tissue Structure and Development	abnormal morphology of marginal zone of spleen	1.87E-04
Organ Morphology	abnormal morphology of marginal zone of spleen	1.87E-04
Hematological System Development and Function	chemotaxis of phagocytes	1.91E-04
Immune Cell Trafficking	chemotaxis of phagocytes	1.91E-04
Inflammatory Response	chemotaxis of phagocytes	1.91E-04
Cellular Movement	chemotaxis of phagocytes	1.91E-04
Hematological System Development and Function	inhibition of T lymphocytes	1.94E-04
Cellular Growth and Proliferation	inhibition of T lymphocytes	1.94E-04
Inflammatory Response	innate immune response	2.05E-04
Hematological System Development and Function	infiltration of leukocytes	2.14E-04
Immune Cell Trafficking	infiltration of leukocytes	2.14E-04
Cellular Movement	infiltration of leukocytes	2.14E-04
Hematological System Development and Function	quantity of antigen presenting cells	2.14E-04
Tissue Morphology	quantity of antigen presenting cells	2.14E-04
Lymphoid Tissue Structure and Development	development of lymphatic system component	2.21E-04
Embryonic Development	development of lymphatic system component	2.21E-04
Organ Development	development of lymphatic system component	2.21E-04
Organismal Development	development of lymphatic system component	2.21E-04
Renal and Urological Disease	diabetic nephropathy	2.28E-04
Cell-To-Cell Signaling and Interaction	adhesion of blood cells	2.30E-04
Tissue Development	adhesion of blood cells	2.30E-04
Hematological System Development and Function	chemotaxis of leukocytes	2.30E-04
Immune Cell Trafficking	chemotaxis of leukocytes	2.30E-04
Inflammatory Response	chemotaxis of leukocytes	2.30E-04
Cellular Movement	chemotaxis of leukocytes	2.30E-04
Cell Death and Survival	cell viability of leukocytes	2.33E-04
Cellular Function and Maintenance	function of dendritic cells	2.34E-04
Hematological System Development and Function	development of helper T lymphocytes	2.37E-04
Cellular Function and Maintenance	development of helper T lymphocytes	2.37E-04
Cellular Development	development of helper T lymphocytes	2.37E-04
Hematopoiesis	development of helper T lymphocytes	2.37E-04
Lymphoid Tissue Structure and Development	development of helper T lymphocytes	2.37E-04

Cell-mediated Immune Response	development of helper T lymphocytes	2.37E-04
Infectious Disease	infection of dendritic cells	2.43E-04
Immunological Disease	infection of dendritic cells	2.43E-04
Hematological Disease	infection of dendritic cells	2.43E-04
Cell-To-Cell Signaling and Interaction	adhesion of epithelial cells	2.45E-04
Tissue Development	adhesion of epithelial cells	2.45E-04
Cell-To-Cell Signaling and Interaction	adhesion of vascular endothelial cells	2.45E-04
Tissue Development	adhesion of vascular endothelial cells	2.45E-04
Cardiovascular System Development and Function	adhesion of vascular endothelial cells	2.45E-04
Infectious Disease	infection of antigen presenting cells	2.53E-04
Immunological Disease	infection of antigen presenting cells	2.53E-04
Hematological Disease	infection of antigen presenting cells	2.53E-04
Hematological System Development and Function	Lymphocyte migration	2.56E-04
Immune Cell Trafficking	Lymphocyte migration	2.56E-04
Cellular Movement	Lymphocyte migration	2.56E-04
Infectious Disease	chronic mucocutaneous candidiasis	2.56E-04
Dermatological Diseases and Conditions	chronic mucocutaneous candidiasis	2.56E-04
Hereditary Disorder	chronic mucocutaneous candidiasis	2.56E-04
Cell Morphology	lack of follicular dendritic cells	2.56E-04
Inflammatory Response	lack of follicular dendritic cells	2.56E-04
Lymphoid Tissue Structure and Development	lack of follicular dendritic cells	2.56E-04
Tissue Morphology	lack of follicular dendritic cells	2.56E-04
Organ Morphology	lack of follicular dendritic cells	2.56E-04
Cell Morphology	morphology of nervous tissue cell lines	2.56E-04
Protein Synthesis	quantity of IL-3 in blood	2.56E-04
Cell-To-Cell Signaling and Interaction	responsiveness of T lymphocytes	2.56E-04
Hematological System Development and Function	survival of erythroblasts	2.56E-04
Cell Death and Survival	survival of erythroblasts	2.56E-04
Hematological System Development and Function	survival of megakaryocytes	2.56E-04
Cell Death and Survival	survival of megakaryocytes	2.56E-04
Cell Death and Survival	apoptosis of phagocytes	2.64E-04
Hematological Disease	blood protein disorder	2.65E-04
Hematological System Development and Function	quantity of hematopoietic progenitor cells	3.11E-04
Hematopoiesis	quantity of hematopoietic progenitor cells	3.11E-04
Tissue Morphology	quantity of hematopoietic progenitor cells	3.11E-04
Cell Death and Survival	apoptosis of hematopoietic progenitor cells	3.19E-04
Cancer	multiple myeloma	3.50E-04
Immunological Disease	multiple myeloma	3.50E-04
Hematological Disease	multiple myeloma	3.50E-04
Hematological System Development and Function	function of myeloid cells	3.68E-04
Cellular Function and Maintenance	function of myeloid cells	3.68E-04
Hematological System Development and Function	adhesion of immune cells	3.73E-04
Cell-To-Cell Signaling and Interaction	adhesion of immune cells	3.73E-04
Immune Cell Trafficking	adhesion of immune cells	3.73E-04
Tissue Development	adhesion of immune cells	3.73E-04
Hematological System Development and Function	quantity of dendritic cells	3.82E-04
Inflammatory Response	quantity of dendritic cells	3.82E-04
Tissue Morphology	quantity of dendritic cells	3.82E-04
Hematological System Development and Function	function of macrophages	3.82E-04
Cellular Function and Maintenance	function of macrophages	3.82E-04
Skeletal and Muscular Disorders	arthrogryposis, renal dysfunction, cholestasis	3.83E-04
Hereditary Disorder	arthrogryposis, renal dysfunction, cholestasis	3.83E-04

Hematological System Development and Function	quantity of B-1 lymphocytes	3.87E-04
Tissue Morphology	quantity of B-1 lymphocytes	3.87E-04
Humoral Immune Response	quantity of B-1 lymphocytes	3.87E-04
Cell Death and Survival	apoptosis of microglia	3.88E-04
Lymphoid Tissue Structure and Development	development of lymphoid organ	3.94E-04
Tissue Development	development of lymphoid organ	3.94E-04
Embryonic Development	development of lymphoid organ	3.94E-04
Organ Development	development of lymphoid organ	3.94E-04
Organismal Development	development of lymphoid organ	3.94E-04
Lymphoid Tissue Structure and Development	morphology of lymphoid organ	4.08E-04
Organ Morphology	morphology of lymphoid organ	4.08E-04
Hematological System Development and Function	cell movement of neutrophils	4.15E-04
Immune Cell Trafficking	cell movement of neutrophils	4.15E-04
Inflammatory Response	cell movement of neutrophils	4.15E-04
Cellular Movement	cell movement of neutrophils	4.15E-04
Cellular Growth and Proliferation	formation of blood cells	4.27E-04
Cell-To-Cell Signaling and Interaction	responsiveness of cells	4.31E-04
Infectious Disease	infection of phagocytes	4.33E-04
Immunological Disease	infection of phagocytes	4.33E-04
Hematological Disease	infection of phagocytes	4.33E-04
Humoral Immune Response	production of antibody	4.34E-04
Protein Synthesis	production of antibody	4.34E-04
Lymphoid Tissue Structure and Development	morphology of lymphatic system component	4.51E-04
Cell Death and Survival	cytotoxicity of leukocytes	4.52E-04
Cell Death and Survival	apoptosis of skin cancer cell lines	4.77E-04
Cellular Function and Maintenance	engulfment of cells	4.81E-04
Infectious Disease	replication of vesicular stomatitis virus	4.83E-04
Cancer	melanoma	4.95E-04
Cell-To-Cell Signaling and Interaction	response of mononuclear leukocytes	5.04E-04
Cell Morphology	lack of T lymphocytes	5.26E-04
Hematological System Development and Function	lack of T lymphocytes	5.26E-04
Neurological Disease	autosomal dominant Parkinson disease	5.35E-04
Skeletal and Muscular Disorders	autosomal dominant Parkinson disease	5.35E-04
Hereditary Disorder	autosomal dominant Parkinson disease	5.35E-04
Cell-To-Cell Signaling and Interaction	chemoattraction of fibroblasts	5.35E-04
Cellular Movement	chemoattraction of fibroblasts	5.35E-04
Connective Tissue Development and Function	chemoattraction of fibroblasts	5.35E-04
Hematological System Development and Function	proliferation of hematopoietic progenitor cells	5.38E-04
Cellular Development	proliferation of hematopoietic progenitor cells	5.38E-04
Hematopoiesis	proliferation of hematopoietic progenitor cells	5.38E-04
Cellular Growth and Proliferation	proliferation of hematopoietic progenitor cells	5.38E-04
Hematological System Development and Function	activation of myeloid cells	5.52E-04
Cell-To-Cell Signaling and Interaction	activation of myeloid cells	5.52E-04
Immune Cell Trafficking	activation of myeloid cells	5.52E-04
Inflammatory Response	activation of myeloid cells	5.52E-04
Cancer	plasma cell dyscrasia	5.93E-04
Immunological Disease	plasma cell dyscrasia	5.93E-04
Hematological Disease	plasma cell dyscrasia	5.93E-04
Cell Death and Survival	cell death of neuroglia	6.24E-04
Cell Morphology	abnormal morphology of cytotoxic T cells	6.34E-04
Hematological System Development and Function	abnormal morphology of cytotoxic T cells	6.34E-04
Infectious Disease	infection of lung	6.34E-04

Respiratory Disease	infection of lung	6.34E-04
Infectious Disease	infection of cells	6.44E-04
Hematological System Development and Function	cell movement of granulocytes	6.50E-04
Immune Cell Trafficking	cell movement of granulocytes	6.50E-04
Cellular Movement	cell movement of granulocytes	6.50E-04
Hematological System Development and Function	T cell migration	6.51E-04
Immune Cell Trafficking	T cell migration	6.51E-04
Cell-mediated Immune Response	T cell migration	6.51E-04
Cellular Movement	T cell migration	6.51E-04
Hematological System Development and Function	generation of lymphocytes	6.53E-04
Cellular Growth and Proliferation	generation of lymphocytes	6.53E-04
Tissue Development	generation of lymphocytes	6.53E-04
Hematological System Development and Function	differentiation of helper T lymphocytes	6.56E-04
Cellular Function and Maintenance	differentiation of helper T lymphocytes	6.56E-04
Cellular Development	differentiation of helper T lymphocytes	6.56E-04
Hematopoiesis	differentiation of helper T lymphocytes	6.56E-04
Lymphoid Tissue Structure and Development	differentiation of helper T lymphocytes	6.56E-04
Cell-mediated Immune Response	differentiation of helper T lymphocytes	6.56E-04
Hematological System Development and Function	transmigration of leukocytes	6.75E-04
Immune Cell Trafficking	transmigration of leukocytes	6.75E-04
Cellular Movement	transmigration of leukocytes	6.75E-04
Organismal Functions	recovery of mice	6.93E-04
Humoral Immune Response	quantity of IgG1	6.98E-04
Protein Synthesis	quantity of IgG1	6.98E-04
Lymphoid Tissue Structure and Development	abnormal morphology of lymphoid organ	7.57E-04
Organ Morphology	abnormal morphology of lymphoid organ	7.57E-04
Cancer	adenocarcinoma	7.77E-04
Cell Death and Survival	initiation of apoptosis	8.21E-04
Cell Cycle	arrest in cell cycle progression	8.73E-04
Gene Expression	transactivation	8.88E-04
Hematological System Development and Function	aggregation of phagocytes	8.90E-04
Cell-To-Cell Signaling and Interaction	aggregation of phagocytes	8.90E-04
Immune Cell Trafficking	aggregation of phagocytes	8.90E-04
Inflammatory Response	aggregation of phagocytes	8.90E-04
Tissue Development	aggregation of phagocytes	8.90E-04
Infectious Disease	replication of Picornaviridae	8.90E-04
Hematological System Development and Function	quantity of B lymphocytes	8.95E-04
Tissue Morphology	quantity of B lymphocytes	8.95E-04
Humoral Immune Response	quantity of B lymphocytes	8.95E-04
Cellular Function and Maintenance	function of splenocytes	9.10E-04
Inflammatory Disease	proctitis	9.10E-04
Gastrointestinal Disease	proctitis	9.10E-04
Cell Death and Survival	delay in cell death	9.12E-04
Cancer	Skin Cancer	9.25E-04
Dermatological Diseases and Conditions	Skin Cancer	9.25E-04
Cell Morphology	abnormal morphology of cells	9.41E-04
Hematological System Development and Function	infiltration by T lymphocytes	9.53E-04
Immune Cell Trafficking	infiltration by T lymphocytes	9.53E-04
Cell-mediated Immune Response	infiltration by T lymphocytes	9.53E-04
Cellular Movement	infiltration by T lymphocytes	9.53E-04
Inflammatory Response	antigen presentation	9.96E-04
Antigen Presentation	antigen presentation	9.96E-04

Humoral Immune Response	class switching	1.13E-03
Lymphoid Tissue Structure and Development	abnormal morphology of periarteriolar lymphoid shea	1.13E-03
Organ Morphology	abnormal morphology of periarteriolar lymphoid shea	1.13E-03
Cell Death and Survival	cell death of oligodendrocyte precursor cells	1.13E-03
Cell Death and Survival	cell death of peripheral T lymphocyte	1.13E-03
Lymphoid Tissue Structure and Development	quantity of lymphatic system cells	1.15E-03
Tissue Morphology	quantity of lymphatic system cells	1.15E-03
Immunological Disease	immunodeficiency	1.18E-03
Cell-To-Cell Signaling and Interaction	phagocytosis of cells	1.19E-03
Cellular Function and Maintenance	phagocytosis of cells	1.19E-03
Inflammatory Response	phagocytosis of cells	1.19E-03
Inflammatory Response	cell-mediated response	1.19E-03
Protein Synthesis	quantity of IFNG in blood	1.20E-03
Immunological Disease	lymphoproliferative disorder	1.21E-03
Hematological System Development and Function	differentiation of Th1 cells	1.23E-03
Cellular Function and Maintenance	differentiation of Th1 cells	1.23E-03
Cellular Development	differentiation of Th1 cells	1.23E-03
Hematopoiesis	differentiation of Th1 cells	1.23E-03
Lymphoid Tissue Structure and Development	differentiation of Th1 cells	1.23E-03
Cell-mediated Immune Response	differentiation of Th1 cells	1.23E-03
Immunological Disease	polyarticular juvenile rheumatoid arthritis	1.28E-03
Inflammatory Disease	polyarticular juvenile rheumatoid arthritis	1.28E-03
Connective Tissue Disorders	polyarticular juvenile rheumatoid arthritis	1.28E-03
Skeletal and Muscular Disorders	polyarticular juvenile rheumatoid arthritis	1.28E-03
Inflammatory Response	antiviral response of cells	1.29E-03
Antimicrobial Response	antiviral response of cells	1.29E-03
Cancer	malignant cutaneous melanoma cancer	1.37E-03
Dermatological Diseases and Conditions	malignant cutaneous melanoma cancer	1.37E-03
Tissue Development	morphology of photoreceptor layer	1.38E-03
Embryonic Development	morphology of photoreceptor layer	1.38E-03
Organ Development	morphology of photoreceptor layer	1.38E-03
Organismal Development	morphology of photoreceptor layer	1.38E-03
Organ Morphology	morphology of photoreceptor layer	1.38E-03
Visual System Development and Function	morphology of photoreceptor layer	1.38E-03
Cell-To-Cell Signaling and Interaction	targeting of cells	1.38E-03
Hematological System Development and Function	cell movement of microglia	1.38E-03
Immune Cell Trafficking	cell movement of microglia	1.38E-03
Inflammatory Response	cell movement of microglia	1.38E-03
Cellular Movement	cell movement of microglia	1.38E-03
Cancer	precursor T-cell lymphoblastic leukemia-lymphoma	1.38E-03
Immunological Disease	precursor T-cell lymphoblastic leukemia-lymphoma	1.38E-03
Hematological Disease	precursor T-cell lymphoblastic leukemia-lymphoma	1.38E-03
Lymphoid Tissue Structure and Development	development of lymph node	1.39E-03
Tissue Development	development of lymph node	1.39E-03
Embryonic Development	development of lymph node	1.39E-03
Organ Development	development of lymph node	1.39E-03
Organismal Development	development of lymph node	1.39E-03
Hematological System Development and Function	transmigration of phagocytes	1.39E-03
Immune Cell Trafficking	transmigration of phagocytes	1.39E-03
Inflammatory Response	transmigration of phagocytes	1.39E-03
Cellular Movement	transmigration of phagocytes	1.39E-03
Cell Death and Survival	antiapoptosis	1.42E-03

Humoral Immune Response	quantity of immunoglobulin	1.42E-03
Protein Synthesis	quantity of immunoglobulin	1.42E-03
Gastrointestinal Disease	experimentally-induced diabetes	1.50E-03
Endocrine System Disorders	experimentally-induced diabetes	1.50E-03
Metabolic Disease	experimentally-induced diabetes	1.50E-03
Hematological System Development and Function	quantity of phagocytes	1.55E-03
Inflammatory Response	quantity of phagocytes	1.55E-03
Tissue Morphology	quantity of phagocytes	1.55E-03
Cell Death and Survival	killing of lymphocytes	1.58E-03
Cell-To-Cell Signaling and Interaction	activation of neuroglia	1.60E-03
Nervous System Development and Function	activation of neuroglia	1.60E-03
Hematological System Development and Function	mitogenesis of T lymphocytes	1.65E-03
Cell Cycle	mitogenesis of T lymphocytes	1.65E-03
Inflammatory Disease	swelling of knee joint	1.65E-03
Inflammatory Response	swelling of knee joint	1.65E-03
Skeletal and Muscular Disorders	swelling of knee joint	1.65E-03
Lymphoid Tissue Structure and Development	abnormal morphology of B-cell follicle	1.68E-03
Lymphoid Tissue Structure and Development	abnormal morphology of Peyer's patches	1.68E-03
Tissue Morphology	abnormal morphology of Peyer's patches	1.68E-03
Organ Morphology	abnormal morphology of Peyer's patches	1.68E-03
Digestive System Development and Function	abnormal morphology of Peyer's patches	1.68E-03
Free Radical Scavenging	release of superoxide	1.68E-03
Molecular Transport	release of superoxide	1.68E-03
Organismal Injury and Abnormalities	cytosis	1.72E-03
Hematological System Development and Function	quantity of thymocytes	1.75E-03
Hematopoiesis	quantity of thymocytes	1.75E-03
Tissue Morphology	quantity of thymocytes	1.75E-03
Hematological System Development and Function	quantity of plasma cells	1.90E-03
Tissue Morphology	quantity of plasma cells	1.90E-03
Humoral Immune Response	quantity of plasma cells	1.90E-03
Humoral Immune Response	quantity of IgG2a	1.94E-03
Protein Synthesis	quantity of IgG2a	1.94E-03
Infectious Disease	infection by DNA virus	1.97E-03
Immunological Disease	hypoplasia of thymus gland	2.01E-03
Developmental Disorder	hypoplasia of thymus gland	2.01E-03
Embryonic Development	development of face	2.02E-03
Organismal Development	development of face	2.02E-03
Hematological System Development and Function	migration of leukocyte cell lines	2.02E-03
Immune Cell Trafficking	migration of leukocyte cell lines	2.02E-03
Cellular Movement	migration of leukocyte cell lines	2.02E-03
Hematological System Development and Function	development of hematopoietic progenitor cells	2.02E-03
Cellular Development	development of hematopoietic progenitor cells	2.02E-03
Hematopoiesis	development of hematopoietic progenitor cells	2.02E-03
Hematological System Development and Function	cell movement of macrophages	2.03E-03
Immune Cell Trafficking	cell movement of macrophages	2.03E-03
Inflammatory Response	cell movement of macrophages	2.03E-03
Cellular Movement	cell movement of macrophages	2.03E-03
Cell Death and Survival	apoptosis of thymocytes	2.07E-03
Immunological Disease	eosinophilia	2.12E-03
Hematological Disease	eosinophilia	2.12E-03
Cell Death and Survival	cytotoxicity of lymphocytes	2.18E-03
Hematological System Development and Function	development of B lymphocytes	2.23E-03

Cellular Development	development of B lymphocytes	2.23E-03
Hematopoiesis	development of B lymphocytes	2.23E-03
Lymphoid Tissue Structure and Development	development of B lymphocytes	2.23E-03
Humoral Immune Response	development of B lymphocytes	2.23E-03
Hematological System Development and Function	infiltration by mononuclear leukocytes	2.23E-03
Immune Cell Trafficking	infiltration by mononuclear leukocytes	2.23E-03
Cellular Movement	infiltration by mononuclear leukocytes	2.23E-03
Cell Morphology	abnormal morphology of intraepithelial T lymphocytes	2.26E-03
Hematological System Development and Function	abnormal morphology of intraepithelial T lymphocytes	2.26E-03
Hematological System Development and Function	differentiation of gamma-delta T lymphocytes	2.26E-03
Cellular Function and Maintenance	differentiation of gamma-delta T lymphocytes	2.26E-03
Cellular Development	differentiation of gamma-delta T lymphocytes	2.26E-03
Hematopoiesis	differentiation of gamma-delta T lymphocytes	2.26E-03
Lymphoid Tissue Structure and Development	differentiation of gamma-delta T lymphocytes	2.26E-03
Cell-mediated Immune Response	differentiation of gamma-delta T lymphocytes	2.26E-03
Cellular Function and Maintenance	exocytosis of Weibel-Palade bodies	2.26E-03
Molecular Transport	exocytosis of Weibel-Palade bodies	2.26E-03
Cellular Assembly and Organization	exocytosis of Weibel-Palade bodies	2.26E-03
Infectious Disease	replication of encephalomyocarditis virus	2.26E-03
Cell Death and Survival	apoptosis of neuroglia	2.31E-03
Infectious Disease	endotoxicosis	2.31E-03
Infectious Disease	flu	2.31E-03
Immunological Disease	Sjogren's syndrome	2.38E-03
Gastrointestinal Disease	Sjogren's syndrome	2.38E-03
Ophthalmic Disease	Sjogren's syndrome	2.38E-03
Cancer	infection of tumor cell lines	2.44E-03
Infectious Disease	infection of tumor cell lines	2.44E-03
Cell Death and Survival	cell death of dendritic cells	2.46E-03
Hematological System Development and Function	quantity of double-positive thymocyte	2.46E-03
Hematopoiesis	quantity of double-positive thymocyte	2.46E-03
Tissue Morphology	quantity of double-positive thymocyte	2.46E-03
Neurological Disease	Huntington's disease	2.49E-03
Skeletal and Muscular Disorders	Huntington's disease	2.49E-03
Hereditary Disorder	Huntington's disease	2.49E-03
Hematological System Development and Function	generation of T lymphocytes	2.55E-03
Cellular Growth and Proliferation	generation of T lymphocytes	2.55E-03
Tissue Development	generation of T lymphocytes	2.55E-03
Neurological Disease	neurological signs	2.57E-03
Inflammatory Response	degranulation of cells	2.57E-03
Cellular Compromise	degranulation of cells	2.57E-03
Hematological System Development and Function	aggregation of dendritic cells	2.60E-03
Cell-To-Cell Signaling and Interaction	aggregation of dendritic cells	2.60E-03
Immune Cell Trafficking	aggregation of dendritic cells	2.60E-03
Inflammatory Response	aggregation of dendritic cells	2.60E-03
Tissue Development	aggregation of dendritic cells	2.60E-03
Lymphoid Tissue Structure and Development	lack of Peyer's patches	2.60E-03
Tissue Morphology	lack of Peyer's patches	2.60E-03
Organ Morphology	lack of Peyer's patches	2.60E-03
Digestive System Development and Function	lack of Peyer's patches	2.60E-03
Inflammatory Response	antibody response	2.63E-03
Cancer	skin cancer	2.67E-03
Dermatological Diseases and Conditions	skin cancer	2.67E-03

Hematological System Development and Function	cell movement of leukocyte cell lines	2.72E-03
Immune Cell Trafficking	cell movement of leukocyte cell lines	2.72E-03
Cellular Movement	cell movement of leukocyte cell lines	2.72E-03
Cell Death and Survival	apoptosis of tumor cell lines	2.83E-03
Hematological System Development and Function	activation of neutrophils	2.89E-03
Cell-To-Cell Signaling and Interaction	activation of neutrophils	2.89E-03
Immune Cell Trafficking	activation of neutrophils	2.89E-03
Inflammatory Response	activation of neutrophils	2.89E-03
Cellular Function and Maintenance	ion homeostasis of cells	2.90E-03
Cellular Development	maturation of dendritic cells	2.95E-03
Hematopoiesis	maturation of dendritic cells	2.95E-03
Hematological System Development and Function	quantity of B-2 lymphocytes	2.96E-03
Tissue Morphology	quantity of B-2 lymphocytes	2.96E-03
Humoral Immune Response	quantity of B-2 lymphocytes	2.96E-03
Infectious Disease	replication of Murine herpesvirus 4	2.96E-03
Cell-To-Cell Signaling and Interaction	immune response of T lymphocytes	3.08E-03
Inflammatory Response	immune response of T lymphocytes	3.08E-03
Cell Death and Survival	cell death of tumor cell lines	3.09E-03
Neurological Disease	movement disorder	3.09E-03
Respiratory Disease	edema of lung	3.11E-03
Organismal Injury and Abnormalities	edema of lung	3.11E-03
Immunological Disease	allergy	3.14E-03
Cell Death and Survival	apoptosis of pancreatic cancer cells	3.35E-03
Tumor Morphology	apoptosis of pancreatic cancer cells	3.35E-03
Hematological System Development and Function	chemoattraction of neutrophils	3.35E-03
Cell-To-Cell Signaling and Interaction	chemoattraction of neutrophils	3.35E-03
Immune Cell Trafficking	chemoattraction of neutrophils	3.35E-03
Inflammatory Response	chemoattraction of neutrophils	3.35E-03
Cellular Movement	chemoattraction of neutrophils	3.35E-03
Neurological Disease	damage of axons	3.35E-03
Hereditary Disorder	familial atrial fibrillation type 9	5.11E-03
Cardiac Arrythmia	familial atrial fibrillation type 9	5.11E-03
Cardiovascular Disease	familial atrial fibrillation type 9	5.11E-03
Renal and Urological Disease	septic acute kidney injury	5.11E-03
Kidney Failure	septic acute kidney injury	5.11E-03
Inflammatory Disease	Nephritis	7.87E-03
Inflammatory Response	Nephritis	7.87E-03
Renal and Urological Disease	Nephritis	7.87E-03
Renal Inflammation	Nephritis	7.87E-03
Renal Nephritis	Nephritis	7.87E-03
Inflammatory Disease	Hepatitis	8.43E-03
Gastrointestinal Disease	Hepatitis	8.43E-03
Hepatic System Disease	Hepatitis	8.43E-03
Liver Hepatitis	Hepatitis	8.43E-03
Cellular Movement	infiltration of kidney	1.02E-02
Renal Cellular Infiltration	infiltration of kidney	1.02E-02
Renal and Urological System Development and Functio	infiltration of kidney	1.02E-02
Renal and Urological Disease	ischemic injury of kidney	1.02E-02
Organismal Injury and Abnormalities	ischemic injury of kidney	1.02E-02
Cardiovascular Disease	ischemic injury of kidney	1.02E-02
Renal Damage	ischemic injury of kidney	1.02E-02
Cell Death and Survival	cell death of renal tubule	1.23E-02

Renal Necrosis/Cell Death	cell death of renal tubule	1.23E-02
Inflammatory Disease	alcoholic hepatitis	1.31E-02
Gastrointestinal Disease	alcoholic hepatitis	1.31E-02
Hepatic System Disease	alcoholic hepatitis	1.31E-02
Liver Hepatitis	alcoholic hepatitis	1.31E-02
Hereditary Disorder	short QT syndrome	1.53E-02
Cardiac Arrythmia	short QT syndrome	1.53E-02
Cardiovascular Disease	short QT syndrome	1.53E-02
Cell Death and Survival	cell death of cardiomyocytes	1.67E-02
Cardiac Necrosis/Cell Death	cell death of cardiomyocytes	1.67E-02
Infectious Disease	chronic hepatitis B	2.22E-02
Inflammatory Disease	chronic hepatitis B	2.22E-02
Gastrointestinal Disease	chronic hepatitis B	2.22E-02
Hepatic System Disease	chronic hepatitis B	2.22E-02
Liver Hepatitis	chronic hepatitis B	2.22E-02
Cancer	transformation of endocardial cells	2.53E-02
Cardiac Transformation	transformation of endocardial cells	2.53E-02
Organismal Injury and Abnormalities	bleeding of heart	3.03E-02
Cardiovascular Disease	bleeding of heart	3.03E-02
Cardiac Hemorrhaging	bleeding of heart	3.03E-02
Inflammatory Disease	nephrotoxic nephritis	3.03E-02
Inflammatory Response	nephrotoxic nephritis	3.03E-02
Renal and Urological Disease	nephrotoxic nephritis	3.03E-02
Renal Inflammation	nephrotoxic nephritis	3.03E-02
Renal Nephritis	nephrotoxic nephritis	3.03E-02
Cellular Development	proliferation of renal tubular epithelial cells	3.52E-02
Cellular Growth and Proliferation	proliferation of renal tubular epithelial cells	3.52E-02
Renal and Urological System Development and Functio	proliferation of renal tubular epithelial cells	3.52E-02
Renal Proliferation	proliferation of renal tubular epithelial cells	3.52E-02
Inflammatory Disease	tubular nephritis	3.52E-02
Inflammatory Response	tubular nephritis	3.52E-02
Renal and Urological Disease	tubular nephritis	3.52E-02
Renal Inflammation	tubular nephritis	3.52E-02
Renal Nephritis	tubular nephritis	3.52E-02
Cell Death and Survival	apoptosis of cardiomyocytes	3.71E-02
Cardiac Necrosis/Cell Death	apoptosis of cardiomyocytes	3.71E-02
Renal and Urological Disease	failure of kidney	3.90E-02
Organismal Injury and Abnormalities	failure of kidney	3.90E-02
Kidney Failure	failure of kidney	3.90E-02
Cancer	tumorigenesis of hepatocellular carcinoma	4.01E-02
Gastrointestinal Disease	tumorigenesis of hepatocellular carcinoma	4.01E-02
Hepatic System Disease	tumorigenesis of hepatocellular carcinoma	4.01E-02
Hepatocellular Carcinoma	tumorigenesis of hepatocellular carcinoma	4.01E-02
Liver Hyperplasia/Hyperproliferation	tumorigenesis of hepatocellular carcinoma	4.01E-02
Inflammatory Disease	chronic hepatitis	4.60E-02
Gastrointestinal Disease	chronic hepatitis	4.60E-02
Hepatic System Disease	chronic hepatitis	4.60E-02
Liver Hepatitis	chronic hepatitis	4.60E-02
Cellular Development	proliferation of hepatic stellate cells	4.99E-02
Cellular Growth and Proliferation	proliferation of hepatic stellate cells	4.99E-02
Connective Tissue Development and Function	proliferation of hepatic stellate cells	4.99E-02
Hepatic System Development and Function	proliferation of hepatic stellate cells	4.99E-02

Liver Fibrosis	proliferation of hepatic stellate cells	4.99E-02
Liver Proliferation	proliferation of hepatic stellate cells	4.99E-02
Renal and Urological Disease	ischemic acute renal failure	4.99E-02
Kidney Failure	ischemic acute renal failure	4.99E-02

both group of septic shock patients.

Molecules	Molecules
ANKRD9,AQP3,BAG1,BCL11B,C14orf159,CA2,CARD16,CASP1,CD274,CD52,CFI	66
AGTRAP,ALOX12,ANKRD9,CA2,CAMK1D,CARD16,CD274,CD46,CX3CR1,DDX58	44
CASP1,CD274,CFD,CLEC7A,CX3CR1,DDX58,F2RL1,GZMA,HLA-DQB1,IGHM,IRI	19
BCL11B,CD274,CD46,HLA-DQA1,HLA-DQB1,IGHM,IL7R,IRF1 (includes EG:1636	11
BCL11B,CD274,CD46,HLA-DQA1,HLA-DQB1,IGHM,IL7R,IRF1 (includes EG:1636	11
BCL11B,CD274,CD46,HLA-DQA1,HLA-DQB1,IGHM,IL7R,IRF1 (includes EG:1636	13
APOL6,AQP3,CFD,CLEC7A,DDX58,EPSTI1,FAM26F,FCGR3B,GBP1,GBP2 (inclu	22
BCL11B,CASP1,CD274,CD46,CLEC7A,CX3CR1,F2RL1,GZMA,HLA-DQA1,HLA-D	25
BCL11B,CASP1,CD274,CD46,CLEC7A,CX3CR1,F2RL1,GZMA,HLA-DQA1,HLA-D	25
BTN3A1,BTN3A2,BTN3A3,CASP1,CD274,CLEC2B,F2RL1,GALNT2,GBP2 (include	27
BAG1,BCL11B,CASP1,CD274,FGL2,GIMAP4,GZMA,HSP90AB1,IGHM,IL7R,IRF1	24
CASP1,CD274,CX3CR1,F2RL1,HLA-DQA1,HLA-DQB1,IGHM,IRF1 (includes EG:1	13
CASP1,CD274,CX3CR1,F2RL1,HLA-DQA1,HLA-DQB1,IGHM,IRF1 (includes EG:1	13
CASP1,CD274,CX3CR1,F2RL1,HLA-DQA1,HLA-DQB1,IGHM,IRF1 (includes EG:1	13
ANKRD9,AQP3,BAG1,CA2,CARD16,CASP1,CD274,CFD,EPB42,F2RL1,FGL2,GB	47
AK1,APOL6,AQP3,BCL11B,CA2,CASP1,CD274,CLEC7A,F2RL1,FCGR3B,GIMAP	34
BCL11B,CD274,CD46,DDX58,HLA-DQA1,HLA-DQB1,IGHM,IL7R,IRF1 (includes E	15
ANKRD9,AQP3,BAG1,CA2,CARD16,CASP1,CD274,CFD,EPB42,F2RL1,FGL2,GB	45
BCL11B,CD274,HLA-DQA1,HLA-DQB1,IGHM,IRF1 (includes EG:16362),LCK,LY7	9
BCL11B,CD274,HLA-DQA1,HLA-DQB1,IGHM,IRF1 (includes EG:16362),LCK,LY7	9
BCL11B,CD274,CD46,CLEC7A,F2RL1,GZMA,HLA-DQA1,HLA-DQB1,IGHM,IRF1 (	22
BCL11B,CD274,CD46,CLEC7A,F2RL1,GZMA,HLA-DQA1,HLA-DQB1,IGHM,IRF1 (	22
BCL11B,CD274,CD46,CLEC7A,F2RL1,GZMA,HLA-DQA1,HLA-DQB1,IGHM,IRF1 (	22
BCL11B,CD274,CD46,CLEC7A,F2RL1,GZMA,HLA-DQA1,HLA-DQB1,IGHM,IRF1 (	22
CD274,CLEC2B,F2RL1,GALNT2,GBP2 (includes EG:14468),GZMA,HLA-DPB1,HL	22
CD274,CLEC2B,F2RL1,GALNT2,GBP2 (includes EG:14468),GZMA,HLA-DPB1,HL	22
CD274,CLEC2B,F2RL1,GALNT2,GBP2 (includes EG:14468),GZMA,HLA-DPB1,HL	22
CD274,CLEC2B,F2RL1,GALNT2,GBP2 (includes EG:14468),GZMA,HLA-DPB1,HL	22
ALOX12,ANKRD9,AQP3,BAG1,CA2,CARD16,CASP1,CD274,CFD,EPB42,F2RL1,I	46
BCL11B,CD274,DDX58,HLA-DQA1,HLA-DQB1,IGHM,IRF1 (includes EG:16362),L	12
BCL11B,CD274,CD46,DDX58,EPB42,HLA-DQA1,HLA-DQB1,IGHM,IL7R,IRF1 (inc	16
CASP1,CD274,CLEC2B,F2RL1,GALNT2,GBP2 (includes EG:14468),GZMA,HLA-D	26
CASP1,CD274,CLEC2B,F2RL1,GALNT2,GBP2 (includes EG:14468),GZMA,HLA-D	26
CASP1,CD274,CLEC2B,F2RL1,GALNT2,GBP2 (includes EG:14468),GZMA,HLA-D	26
CASP1,CD274,CLEC7A,CX3CR1,DDX58,GIMAP4,GZMA,HLA-DQB1,IGHM,IRF1 (	18
BCL11B,CASP1,CD274,CD46,CLEC7A,CX3CR1,F2RL1,GZMA,HLA-DQA1,HLA-D	27
ALOX12,BCL11B,CASP1,CD274,DDX58,F2RL1,HLA-DQA1,IGHM,ITGA4,LTB,MM	15
ALOX12,BCL11B,CASP1,CD274,DDX58,F2RL1,HLA-DQA1,IGHM,ITGA4,LTB,MM	15
AK1,BCL11B,BTN3A1,CA2,CASP1,CD52,EPSTI1,F2RL1,GBP1,HLA-DQA1,HLA-D	24
AK1,BCL11B,BTN3A1,CA2,CASP1,CD52,EPSTI1,F2RL1,GBP1,HLA-DQA1,HLA-D	24
BCL11B,CD274,HLA-DQA1,HLA-DQB1,IGHM,IRF1 (includes EG:16362),LCK,LY7	10
CASP1,CD274,CLEC2B,F2RL1,GALNT2,GBP2 (includes EG:14468),GZMA,HLA-D	27
CASP1,CD274,CLEC2B,F2RL1,GALNT2,GBP2 (includes EG:14468),GZMA,HLA-D	27
CASP1,CD274,CLEC2B,F2RL1,GALNT2,GBP2 (includes EG:14468),GZMA,HLA-D	27
BCL11B,CASP1,CD274,GIMAP4,HLA-DQA1,HLA-DQB1,IGHM,IL7R,IRF1 (include	19
BCL11B,CASP1,CD274,GIMAP4,HLA-DQA1,HLA-DQB1,IGHM,IL7R,IRF1 (include	19
BCL11B,CASP1,CD274,GIMAP4,HLA-DQA1,HLA-DQB1,IGHM,IL7R,IRF1 (include	19
CD52,CFD,F2RL1,HLA-DPB1,HLA-DQB1,IRF1 (includes EG:16362),LTB,MX1	8

CD52,CFD,F2RL1,HLA-DPB1,HLA-DQB1,IRF1 (includes EG:16362),LTB,MX1	8
CD52,CFD,F2RL1,HLA-DPB1,HLA-DQB1,IRF1 (includes EG:16362),LTB,MX1	8
BCL11B,CASP1,CD274,GIMAP4,HLA-DQA1,HLA-DQB1,IGHM,IL7R,IRF1 (includes	17
BCL11B,CASP1,CD274,GIMAP4,HLA-DQA1,HLA-DQB1,IGHM,IL7R,IRF1 (includes	17
BCL11B,CASP1,CD274,GIMAP4,HLA-DQA1,HLA-DQB1,IGHM,IL7R,IRF1 (includes	17
BCL11B,CASP1,CD274,GIMAP4,HLA-DQA1,HLA-DQB1,IGHM,IL7R,IRF1 (includes	17
BCL11B,CASP1,CD274,GIMAP4,HLA-DQA1,HLA-DQB1,IL7R,IRF1 (includes EG:1	16
BCL11B,CASP1,CD274,GIMAP4,HLA-DQA1,HLA-DQB1,IL7R,IRF1 (includes EG:1	16
BCL11B,CASP1,CD274,GIMAP4,HLA-DQA1,HLA-DQB1,IL7R,IRF1 (includes EG:1	16
BCL11B,CASP1,CD274,GIMAP4,HLA-DQA1,HLA-DQB1,IL7R,IRF1 (includes EG:1	16
BCL11B,CASP1,CD274,GIMAP4,HLA-DQA1,HLA-DQB1,IL7R,IRF1 (includes EG:1	16
BCL11B,CASP1,CD274,GIMAP4,HLA-DQA1,HLA-DQB1,IL7R,IRF1 (includes EG:1	16
BCL11B,F2RL1,HAL,HLA-DQA1,IGHM,IGKV1D-8,KRT1,LTB,MME,MX1,PDE4B,PE	16
ALOX12,CA2,CARD16,CASP1,CD274,CLEC2B,DDX58,F2RL1,HLA-DQB1,IGHM,I	23
ALOX12,AQP3,BAG1,BCL11B,CA2,CD274,DDX58,GAS2L1,GIMAP4,HEMGN,HLA	35
ALOX12,ANKRD44,BTN3A1,BTN3A2,BTN3A3,CA2,CD274,CD52,CLEC2B,HLA-DF	21
ALOX12,ANKRD44,BTN3A1,BTN3A2,BTN3A3,CA2,CD274,CD52,CLEC2B,HLA-DF	21
ALOX12,ANKRD44,BTN3A1,BTN3A2,BTN3A3,CA2,CD274,CD52,CLEC2B,HLA-DF	21
BCL11B,CASP1,CD274,FGL2,GIMAP4,GZMA,HSP90AB1,IGHM,IL7R,IRF1 (includ	17
BCL11B,CD274,CX3CR1,DDX58,EPB42,F2RL1,HLA-DQA1,HLA-DQB1,IGHM,IL7F	24
BCL11B,CD274,CX3CR1,DDX58,EPB42,F2RL1,HLA-DQA1,HLA-DQB1,IGHM,IL7F	24
ALOX12,APOL6,AQP3,BAG1,BCL11B,CAMK1D,CASP1,CD274,CD46,CX3CR1,DD	54
BCL11B,CASP1,CD274,CD52,DDX58,EPSTI1,HCAR3,IFIT2,IFIT3,IL7R,IRF1 (inclu	22
BCL11B,CASP1,CD274,CD52,DDX58,EPSTI1,HCAR3,IFIT2,IFIT3,IL7R,IRF1 (inclu	22
ALOX12,APOL6,AQP3,BAG1,BCL11B,CAMK1D,CASP1,CD274,CX3CR1,DDX58,F	46
BTN3A1,CD274,CD46,F2RL1,FGL2,HLA-DQA1,HLA-DQB1,IGHM,IL32,IL7R,IRF1 (	22
BTN3A1,CD274,CD46,F2RL1,FGL2,HLA-DQA1,HLA-DQB1,IGHM,IL32,IL7R,IRF1 (	22
BCL11B,CD274,CX3CR1,DDX58,F2RL1,HLA-DQA1,HLA-DQB1,IGHM,IL7R,IRF1 (	22
BCL11B,CD274,CX3CR1,DDX58,F2RL1,HLA-DQA1,HLA-DQB1,IGHM,IL7R,IRF1 (	22
BCL11B,CD274,HLA-DQA1,HLA-DQB1,IGHM,IRF1 (includes EG:16362),LCK,PSM	8
BCL11B,CD274,HLA-DQA1,HLA-DQB1,IGHM,IRF1 (includes EG:16362),LCK,PSM	8
BTN3A1,CD274,CD46,F2RL1,FGL2,HLA-DQA1,HLA-DQB1,IGHM,IL7R,IRF1 (inclu	20
BTN3A1,CD274,CD46,F2RL1,FGL2,HLA-DQA1,HLA-DQB1,IGHM,IL7R,IRF1 (inclu	20
BTN3A1,CD274,CD46,F2RL1,FGL2,HLA-DQA1,HLA-DQB1,IGHM,IL7R,IRF1 (inclu	20
BTN3A1,CD274,CD46,F2RL1,FGL2,HLA-DQA1,HLA-DQB1,IGHM,IL7R,IRF1 (inclu	21
BTN3A1,CD274,CD46,F2RL1,FGL2,HLA-DQA1,HLA-DQB1,IGHM,IL7R,IRF1 (inclu	21
BTN3A1,CD274,CD46,F2RL1,FGL2,HLA-DQA1,HLA-DQB1,IGHM,IL7R,IRF1 (inclu	21
AGTRAP,ALOX12,BAG1,BCL11B,BTN3A1,CASP1,CD274,CD46,CLEC7A,F2RL1,F	53
BAG1,EPB42,IFIT3,LCK,MME,PRKCH,SELENBP1,SLC6A8,TCN1,TPM1 (includes	12
BAG1,EPB42,IFIT3,LCK,MME,PRKCH,SELENBP1,SLC6A8,TCN1,TPM1 (includes	12
CLEC7A,F2RL1,HLA-DQA1,HLA-DQB1,IGHM,LTB,PSMB9,RORA,SLPI,SNCA,STA	11
CLEC7A,F2RL1,HLA-DQA1,HLA-DQB1,IGHM,LTB,PSMB9,RORA,SLPI,SNCA,STA	11
CLEC7A,F2RL1,HLA-DQA1,HLA-DQB1,IGHM,LTB,PSMB9,RORA,SLPI,SNCA,STA	11
CLEC7A,F2RL1,HLA-DQA1,HLA-DQB1,IGHM,LTB,PSMB9,RORA,SLPI,SNCA,STA	11
CASP1,CD52,EPSTI1,F2RL1,HLA-DQA1,HLA-DQB1,IL7R,ITGA4,MX1,TAP2	10
CASP1,CD52,EPSTI1,F2RL1,HLA-DQA1,HLA-DQB1,IL7R,ITGA4,MX1,TAP2	10
CASP1,CD52,EPSTI1,F2RL1,HLA-DQA1,HLA-DQB1,IL7R,ITGA4,MX1,TAP2	10
CASP1,CD52,EPSTI1,F2RL1,HLA-DQA1,HLA-DQB1,HSPB1,IL7R,ITGA4,LRRK2,M	15
BCL11B,CD274,DDX58,GAS2L1,GIMAP4,HLA-DQA1,HLA-DQB1,IGHM,IL7R,IRF1	19
BCL11B,CD274,DDX58,GAS2L1,GIMAP4,HLA-DQA1,HLA-DQB1,IGHM,IL7R,IRF1	19
ALOX12,BAG1,BCL11B,CAMK1D,CASP1,CD274,CX3CR1,DDX58,F2RL1,FGL2,G	35
ALOX12,ANKRD44,BTN3A1,BTN3A2,BTN3A3,CA2,CASP1,CD274,CD52,CFD,CLE	23

AGTRAP,ALOX12,AQP3,BAG1,CA2,CASP1,F2RL1,HSP90AB1,HSPB1,IL7R,LCK,	27
AGTRAP,ALOX12,AQP3,BAG1,CA2,CASP1,F2RL1,HSP90AB1,HSPB1,IL7R,LCK,	28
CASP1,CD274,CLEC7A,DDX58,IRF1 (includes EG:16362),LCN2,LTB,PTGER4,RA	11
ALOX12,BAG1,BCL11B,CAMK1D,CASP1,CD274,CX3CR1,DDX58,F2RL1,GALNT2	32
CARD16,CD274,CD52,GBP2 (includes EG:14468),GZMA,HSP90AB1,IGHM,IL7R,I	21
CARD16,CD274,CD52,GBP2 (includes EG:14468),GZMA,HSP90AB1,IGHM,IL7R,I	21
BTN3A1,BTN3A2,BTN3A3,CD274,CLEC2B,HLA-DPB1,HLA-DQA1,HLA-DQB1,IGH	11
BTN3A1,BTN3A2,BTN3A3,CD274,CLEC2B,HLA-DPB1,HLA-DQA1,HLA-DQB1,IGH	11
BTN3A1,BTN3A2,BTN3A3,CD274,CLEC2B,HLA-DPB1,HLA-DQA1,HLA-DQB1,IGH	11
BTN3A1,BTN3A2,BTN3A3,CD274,CLEC2B,HLA-DPB1,HLA-DQA1,HLA-DQB1,IGH	11
BCL11B,CD274,CD52,DDX58,EPSTI1,HCAR3,IFIT2,IFIT3,IL7R,IRF1 (includes EG	21
ALOX12,BAG1,BCL11B,CASP1,CD274,DDX58,F2RL1,FGL2,GIMAP4,GZMA,HSP	44
BCL11B,CD274,DDX58,GIMAP4,HLA-DQA1,HLA-DQB1,IGHM,IL7R,IRF1 (includes	15
BCL11B,CD274,DDX58,GIMAP4,HLA-DQA1,HLA-DQB1,IGHM,IL7R,IRF1 (includes	15
BCL11B,CD274,DDX58,GIMAP4,HLA-DQA1,HLA-DQB1,IGHM,IL7R,IRF1 (includes	15
BCL11B,F2RL1,HLA-DQA1,IGHM,IGKV1D-8,KRT1,MME,MX1,PDE4B,PECAM1,S	12
DDX58,IFIT2,IRF1 (includes EG:16362),LCN2,MX1,RARRES3,STAT1	7
DDX58,IFIT2,IRF1 (includes EG:16362),LCN2,MX1,RARRES3,STAT1	7
ALOX12,CASP1,CD274,DDX58,F2RL1,IGHM,LTB,MME,PDE4B,PTGER4	10
ALOX12,CASP1,CD274,DDX58,F2RL1,IGHM,LTB,MME,PDE4B,PTGER4	10
ALOX12,CASP1,CD274,DDX58,F2RL1,IGHM,LTB,MME,PDE4B,PTGER4	10
BCL11B,CASP1,F2RL1,HLA-DQA1,IGHM,IGKV1D-8,KRT1,LTB,MME,MX1,PDE4B	13
BCL11B,CASP1,F2RL1,HLA-DQA1,IGHM,IGKV1D-8,KRT1,LTB,MME,MX1,PDE4B	13
BCL11B,CASP1,CD274,FGL2,GIMAP4,GZMA,IGHM,IL7R,LCK,LCN2,PECAM1,PF	13
BCL11B,CD274,DDX58,GIMAP4,HLA-DQA1,HLA-DQB1,IGHM,IL7R,IRF1 (includes	14
BCL11B,CD274,DDX58,GIMAP4,HLA-DQA1,HLA-DQB1,IGHM,IL7R,IRF1 (includes	14
BCL11B,CD274,DDX58,GIMAP4,HLA-DQA1,HLA-DQB1,IGHM,IL7R,IRF1 (includes	14
BCL11B,CAMK1D,CASP1,CD274,CX3CR1,F2RL1,ITGA4,LCK,LRRK2,LTB,OLFM4	20
BCL11B,CAMK1D,CASP1,CD274,CX3CR1,F2RL1,ITGA4,LCK,LRRK2,LTB,OLFM4	20
BCL11B,CAMK1D,CASP1,CD274,CX3CR1,F2RL1,ITGA4,LCK,LRRK2,LTB,OLFM4	20
BCL11B,CASP1,CD274,F2RL1,FGL2,IGHM,IL32,ITGA4,LTB,OLFM4,PF4,PPBP,R	14
BCL11B,CD274,CD46,DDX58,EPB42,HLA-DQA1,HLA-DQB1,IGHM,IL7R,IRF1 (inc	25
CASP1,CD274,CFD,CLEC2B,F2RL1,GZMA,IGHM,LCN2,LTB,OLFM4,RETN,SLPI,	15
BCL11B,CD274,CD52,DDX58,EPSTI1,HCAR3,IFIT2,IFIT3,IL7R,ITGA4,LCK,PDE4	16
BCL11B,CD274,CD52,DDX58,EPSTI1,HCAR3,IFIT2,IFIT3,IL7R,ITGA4,LCK,PDE4	16
CASP1,CD46,FCGR3B,GZMA,LCK,SGK1,SLAMF7,SNCA,STAT1,TAP2	10
CA2,F2RL1,FGL2,GZMA,HSP90AB1,HSPB1,IFIT3,ITGA4,KRT1,LCN2,PI3,PSMB9	17
CA2,F2RL1,FGL2,GZMA,HSP90AB1,HSPB1,IFIT3,ITGA4,KRT1,LCN2,PI3,PSMB9	17
BCL11B,CD274,DDX58,HLA-DQA1,HLA-DQB1,IGHM,IL7R,IRF1 (includes EG:163	14
BCL11B,CD274,DDX58,HLA-DQA1,HLA-DQB1,IGHM,IL7R,IRF1 (includes EG:163	14
CD274,CD46,IL32,ITGA4,PF4,PTGER4	6
CD274,CD46,IL32,ITGA4,PF4,PTGER4	6
ANKRD9,AQP3,CA2,CASP1,CD274,HSP90AB1,IRF1 (includes EG:16362),KCNJ2,	21
ANKRD9,AQP3,CA2,CASP1,CD274,HSP90AB1,IRF1 (includes EG:16362),KCNJ2,	21
CA2,F2RL1,FGL2,GZMA,LCN2,PI3,SLPI,STAT1,TNFAIP2	9
CA2,F2RL1,FGL2,GZMA,LCN2,PI3,SLPI,STAT1,TNFAIP2	9
CAMK1D,CD274,CLEC7A,DDX58,F2RL1,FCGR3B,IGHM,LY75,PECAM1,PF4,UBE	11
BCL11B,CASP1,CD274,FGL2,GIMAP4,GZMA,IGHM,IL7R,LCK,LCN2,PECAM1,ST	12
BCL11B,F2RL1,HLA-DQA1,IGHM,IGKV1D-8,KRT1,MX1,PDE4B,STAT1,TPM1 (inc	10
BCL11B,F2RL1,HLA-DQA1,IGHM,IGKV1D-8,KRT1,MX1,PDE4B,STAT1,TPM1 (inc	10
BCL11B,F2RL1,HLA-DQA1,IGHM,IGKV1D-8,KRT1,MX1,PDE4B,STAT1,TPM1 (inc	10
CASP1,CD274,CLEC7A,DDX58,IRF1 (includes EG:16362),LTB,PTGER4,SLPI,STA	9

CAMK1D,CASP1,CLEC7A,CX3CR1,F2RL1,IGHM,ITGA4,LCN2,LRRK2,LY75,PDE4	18
BCL11B,CD274,CX3CR1,DDX58,EPB42,F2RL1,HLA-DQA1,HLA-DQB1,HSPB1,IG	29
BCL11B,CASP1,CD274,GIMAP4,GZMA,IGHM,IL7R,LCK,LCN2,PECAM1,STAT1	11
BCL11B,CD274,DDX58,HLA-DQA1,HLA-DQB1,IGHM,IL7R,IRF1 (includes EG:163	16
BCL11B,CD274,DDX58,HLA-DQA1,HLA-DQB1,IGHM,IL7R,IRF1 (includes EG:163	16
HLA-DQB1,IL7R,ITGA4,LTB,PSMB9,RORA,STAT1	7
HLA-DQB1,IL7R,ITGA4,LTB,PSMB9,RORA,STAT1	7
BAG1,CD274,CX3CR1,ITGA4,PECAM1,PPBP,SLPI	7
BCL11B,CD274,CX3CR1,F2RL1,ITGA4,LCK,LTB,OLFM4,PECAM1,PF4,RASGRP1	13
BCL11B,CD274,CX3CR1,F2RL1,ITGA4,LCK,LTB,OLFM4,PECAM1,PF4,RASGRP1	13
BCL11B,CD274,CX3CR1,F2RL1,ITGA4,LCK,LTB,OLFM4,PECAM1,PF4,RASGRP1	13
CD274,CD46,IL32,ITGA4,PTGER4	5
CD274,CD46,IL32,ITGA4,PTGER4	5
PI3,SLPI	2
PI3,SLPI	2
IRF1 (includes EG:16362),STAT1	2
BCL11B,CASP1,CD274,GIMAP4,GZMA,IL7R,LCK,LCN2,PECAM1,STAT1	10
CX3CR1,HLA-DQA1,LTB,ZFP36L2	4
CAMK1D,CASP1,CX3CR1,F2RL1,ITGA4,LRRK2,LTB,OLFM4,PDE4B,PECAM1,PF	15
CAMK1D,CASP1,CX3CR1,F2RL1,ITGA4,LRRK2,LTB,OLFM4,PDE4B,PECAM1,PF	15
CAMK1D,CASP1,CX3CR1,F2RL1,ITGA4,LRRK2,LTB,OLFM4,PDE4B,PECAM1,PF	15
BTN3A1,CD274,CD46,F2RL1,FGL2,HLA-DQA1,HLA-DQB1,IGHM,IL7R,IRF1 (inclu	15
BTN3A1,CD274,CD46,F2RL1,FGL2,HLA-DQA1,HLA-DQB1,IGHM,IL7R,IRF1 (inclu	15
BTN3A1,CD274,CD46,F2RL1,FGL2,HLA-DQA1,HLA-DQB1,IGHM,IL7R,IRF1 (inclu	15
CLEC7A,F2RL1,IGHM,IRF1 (includes EG:16362),LCK,PARP14,RARRES3,RASGR	9
CLEC7A,F2RL1,IGHM,IRF1 (includes EG:16362),LCK,PARP14,RARRES3,RASGR	9
AGTRAP,DDX58,F2RL1,GPR146,HSP90AB1,IRF1 (includes EG:16362),LCK,MX1,	14
ITGA4,LCK,LCN2,LEF1,SLPI,TCN1	6
ITGA4,LCK,LCN2,LEF1,SLPI,TCN1	6
IL7R,IRF1 (includes EG:16362),LCK	3
IL7R,IRF1 (includes EG:16362),LCK	3
CD274,CX3CR1,GIMAP4,HLA-DQB1,IGHM,IRF1 (includes EG:16362),LY75,RASG	10
CD274,CX3CR1,GIMAP4,HLA-DQB1,IGHM,IRF1 (includes EG:16362),LY75,RASG	10
CASP1,CD46,CX3CR1,GZMA,LCK,MME,PF4,SLAMF7,STAT1	9
CD52,GBP2 (includes EG:14468),GZMA,IGHM,IL7R,IRF1 (includes EG:16362),LE	13
CD52,GBP2 (includes EG:14468),GZMA,IGHM,IL7R,IRF1 (includes EG:16362),LE	13
IGHM,IL7R,LEF1,STAT1	4
IGHM,IL7R,LEF1,STAT1	4
IGHM,IL7R,LEF1,STAT1	4
IGHM,IL7R,LEF1,STAT1	4
IGHM,IL7R,LEF1,STAT1	4
BCL11B,CD274,CD46,CLEC7A,GZMA,IGHM,IRF1 (includes EG:16362),LCK,PECA	12
BCL11B,CD274,CD46,CLEC7A,GZMA,IGHM,IRF1 (includes EG:16362),LCK,PECA	12
BCL11B,CD274,CD46,CLEC7A,GZMA,IGHM,IRF1 (includes EG:16362),LCK,PECA	12
BCL11B,CD274,CD46,CLEC7A,GZMA,IGHM,IRF1 (includes EG:16362),LCK,PECA	12
KRT1,LCN2,PI3,SLPI,STAT1,TCN1	6
CD274,HLA-DQA1,HLA-DQB1,IGHM,IRF1 (includes EG:16362),LCK,SEMA4D	7
CD274,HLA-DQA1,HLA-DQB1,IGHM,IRF1 (includes EG:16362),LCK,SEMA4D	7
DDX58,STAT1	2
DDX58,STAT1	2
DDX58,STAT1	2
DDX58,STAT1	2

RARRES3,STAT1	2
ANKRD9,AQP3,BAG1,CA2,CASP1,CD274,HSP90AB1,IRF1 (includes EG:16362),K	25
ANKRD9,AQP3,BAG1,CA2,CASP1,CD274,HSP90AB1,IRF1 (includes EG:16362),K	25
CAMK1D,CASP1,ITGA4,PDE4B,PF4,PPBP,SLPI	7
CAMK1D,CASP1,ITGA4,PDE4B,PF4,PPBP,SLPI	7
CAMK1D,CASP1,ITGA4,PDE4B,PF4,PPBP,SLPI	7
CAMK1D,CASP1,ITGA4,PDE4B,PF4,PPBP,SLPI	7
CAMK1D,CASP1,CX3CR1,F2RL1,ITGA4,LCK,LEF1,LRRK2,PDE4B,PF4,PPBP,SE	13
BCL11B,CD274,HLA-DQA1,IL7R,IRF1 (includes EG:16362),LCK,LEF1,PTGER4,R	10
BCL11B,CD274,HLA-DQA1,IL7R,IRF1 (includes EG:16362),LCK,LEF1,PTGER4,R	10
BCL11B,CD274,HLA-DQA1,IL7R,IRF1 (includes EG:16362),LCK,LEF1,PTGER4,R	10
BCL11B,CD274,HLA-DQA1,IL7R,IRF1 (includes EG:16362),LCK,LEF1,PTGER4,R	10
BCL11B,CD274,HLA-DQA1,IL7R,IRF1 (includes EG:16362),LCK,LEF1,PTGER4,R	10
BCL11B,CD274,HLA-DQA1,IL7R,IRF1 (includes EG:16362),LCK,LEF1,PTGER4,R	10
F2RL1,HAL,IGHM,MX1,PI3,PTGER4	6
CLEC7A,F2RL1,GZMA,LCN2,LTB,PF4,RORA,SLPI,SNCA,STAT1	10
CLEC7A,F2RL1,GZMA,LCN2,LTB,PF4,RORA,SLPI,SNCA,STAT1	10
CLEC7A,F2RL1,GZMA,LCN2,LTB,PF4,RORA,SLPI,SNCA,STAT1	10
CLEC7A,F2RL1,GZMA,LCN2,LTB,PF4,RORA,SLPI,SNCA,STAT1	10
CASP1,CD46,IRF1 (includes EG:16362),RARRES3,STAT1	5
CASP1,CD46,IRF1 (includes EG:16362),RARRES3,STAT1	5
CASP1,CD46,IRF1 (includes EG:16362),RARRES3,STAT1	5
AGTRAP,DDX58,F2RL1,GPR146,MX1,RETN,SGK1,SLPI,STAT1	9
CD52,EPSTI1,ITGA4,MX1	4
CD52,EPSTI1,ITGA4,MX1	4
CD52,EPSTI1,ITGA4,MX1	4
CD274,HLA-DQB1,IGHM,IRF1 (includes EG:16362),PSMB9	5
AK1,BCL11B,BTN3A1,CA2,CASP1,GBP1,HSPB1,LRRK2,MAN1A1,OSBPL8,PDE4	16
ANKRD9,AQP3,CA2,CD274,KCNJ2,LCN2,MME,OLFM4,PECAM1,POLB,PTGER4,	17
ANKRD9,AQP3,CA2,CD274,KCNJ2,LCN2,MME,OLFM4,PECAM1,POLB,PTGER4,	17
CAMK1D,CASP1,CX3CR1,F2RL1,ITGA4,LRRK2,LTB,PDE4B,PECAM1,PF4,PPBP	14
CAMK1D,CASP1,CX3CR1,F2RL1,ITGA4,LRRK2,LTB,PDE4B,PECAM1,PF4,PPBP	14
CAMK1D,CASP1,CX3CR1,F2RL1,ITGA4,LRRK2,LTB,PDE4B,PECAM1,PF4,PPBP	14
CAMK1D,CASP1,CX3CR1,F2RL1,ITGA4,LRRK2,LTB,PDE4B,PECAM1,PF4,PPBP	14
IGHM,IL7R,LEF1,LY75,PECAM1,SEMA4D,SLAMF7,SLPI,STAT1	9
IGHM,IL7R,LEF1,LY75,PECAM1,SEMA4D,SLAMF7,SLPI,STAT1	9
IGHM,IL7R,LEF1,LY75,PECAM1,SEMA4D,SLAMF7,SLPI,STAT1	9
IGHM,IL7R,LEF1,LY75,PECAM1,SEMA4D,SLAMF7,SLPI,STAT1	9
HLA-DPB1,HLA-DQB1,IL7R,LTB,TBC1D9	5
HLA-DPB1,HLA-DQB1,IL7R,LTB,TBC1D9	5
HLA-DPB1,HLA-DQB1,IL7R,LTB,TBC1D9	5
HLA-DPB1,HLA-DQB1,IL7R,LTB,TBC1D9	5
HLA-DPB1,HLA-DQB1,IL7R,LTB,TBC1D9	5
BCL11B,CD274,CD46,CLEC7A,GZMA,LCK,PECAM1,PTGER4,SEMA4D,STAT1	10
BCL11B,CD274,CD46,CLEC7A,GZMA,LCK,PECAM1,PTGER4,SEMA4D,STAT1	10
BCL11B,CD274,CD46,CLEC7A,GZMA,LCK,PECAM1,PTGER4,SEMA4D,STAT1	10
BCL11B,CD274,CD46,CLEC7A,GZMA,LCK,PECAM1,PTGER4,SEMA4D,STAT1	10
IL7R,LEF1,PARP14,PF4,PPBP,SEMA4D,STAT1	7
CASP1,F2RL1,IL32,MME,PDE4B	5
CASP1,F2RL1,IL32,MME,PDE4B	5
CASP1,F2RL1,IL32,MME,PDE4B	5
F2RL1,IL32,PECAM1,STAT1	4

IGHM,SEMA4D	2
IGHM,SEMA4D	2
ALOX12,F2RL1	2
ALOX12,F2RL1	2
ALOX12,F2RL1	2
ALOX12,F2RL1	2
ALOX12,F2RL1	2
ALOX12,F2RL1	2
IL7R,PTGER4	2
F2RL1,FCGR3B,PF4,PPBP	4
F2RL1,FCGR3B,PF4,PPBP	4
BCL11B,CD274,CX3CR1,F2RL1,ITGA4,LCK,LTB,PECAM1,RASGRP1,SEMA4D	10
BCL11B,CD274,CX3CR1,F2RL1,ITGA4,LCK,LTB,PECAM1,RASGRP1,SEMA4D	10
BCL11B,CD274,CX3CR1,F2RL1,ITGA4,LCK,LTB,PECAM1,RASGRP1,SEMA4D	10
CASP1,HSP90AB1,IRF1 (includes EG:16362),LCN2,PF4,PTGER4,STAT1	7
IL7R,LEF1,STAT1	3
CAMK1D,CASP1,CX3CR1,ITGA4,LRRK2,PDE4B,PF4,PPBP,SLPI	9
CAMK1D,CASP1,CX3CR1,ITGA4,LRRK2,PDE4B,PF4,PPBP,SLPI	9
CAMK1D,CASP1,CX3CR1,ITGA4,LRRK2,PDE4B,PF4,PPBP,SLPI	9
CAMK1D,CASP1,CX3CR1,ITGA4,LRRK2,PDE4B,PF4,PPBP,SLPI	9
HLA-DQB1,IGHM,LTB	3
HLA-DQB1,IGHM,LTB	3
CAMK1D,CASP1,CX3CR1,ITGA4,LRRK2,PDE4B,PF4,PPBP,SLPI	9
CAMK1D,CASP1,CX3CR1,ITGA4,LRRK2,PDE4B,PF4,PPBP,SLPI	9
CAMK1D,CASP1,CX3CR1,ITGA4,LRRK2,PDE4B,PF4,PPBP,SLPI	9
CAMK1D,CASP1,CX3CR1,ITGA4,LRRK2,PDE4B,PF4,PPBP,SLPI	9
CD274,CD46,IL32,PTGER4	4
CD274,CD46,IL32,PTGER4	4
CASP1,CLEC7A,DDX58,LCN2,MX1	5
BCL11B,CASP1,CD274,F2RL1,ITGA4,LTB,OLFM4,PF4,PPBP,RETN,STAT1	11
BCL11B,CASP1,CD274,F2RL1,ITGA4,LTB,OLFM4,PF4,PPBP,RETN,STAT1	11
BCL11B,CASP1,CD274,F2RL1,ITGA4,LTB,OLFM4,PF4,PPBP,RETN,STAT1	11
CX3CR1,F2RL1,IGHM,IRF1 (includes EG:16362),LCN2,LTB,PI3,SNCA	8
CX3CR1,F2RL1,IGHM,IRF1 (includes EG:16362),LCN2,LTB,PI3,SNCA	8
BCL11B,EPB42,HLA-DQB1,IGHM,IL7R,IRF1 (includes EG:16362),LTB,PECAM1,S	9
BCL11B,EPB42,HLA-DQB1,IGHM,IL7R,IRF1 (includes EG:16362),LTB,PECAM1,S	9
BCL11B,EPB42,HLA-DQB1,IGHM,IL7R,IRF1 (includes EG:16362),LTB,PECAM1,S	9
BCL11B,EPB42,HLA-DQB1,IGHM,IL7R,IRF1 (includes EG:16362),LTB,PECAM1,S	9
GAS2L1,LEF1,MME,PECAM1,PTGER4,STAT1	6
ALOX12,CD46,FCGR3B,IGHM,ITGA4,LCK,PECAM1,PF4,PPBP,PTGER4,RASGRI	11
ALOX12,CD46,FCGR3B,IGHM,ITGA4,LCK,PECAM1,PF4,PPBP,PTGER4,RASGRI	11
CAMK1D,CASP1,CX3CR1,F2RL1,ITGA4,LRRK2,PDE4B,PF4,PPBP,SLPI	10
CAMK1D,CASP1,CX3CR1,F2RL1,ITGA4,LRRK2,PDE4B,PF4,PPBP,SLPI	10
CAMK1D,CASP1,CX3CR1,F2RL1,ITGA4,LRRK2,PDE4B,PF4,PPBP,SLPI	10
CAMK1D,CASP1,CX3CR1,F2RL1,ITGA4,LRRK2,PDE4B,PF4,PPBP,SLPI	10
IL7R,LEF1,MX1,PARP14,PF4,PPBP,SEMA4D,STAT1	8
CD274,CLEC7A,DDX58,LTB,PTGER4	5
CASP1,IRF1 (includes EG:16362),RORA,STAT1	4
CASP1,IRF1 (includes EG:16362),RORA,STAT1	4
CASP1,IRF1 (includes EG:16362),RORA,STAT1	4
CASP1,IRF1 (includes EG:16362),RORA,STAT1	4
CASP1,IRF1 (includes EG:16362),RORA,STAT1	4

CASP1,IRF1 (includes EG:16362),RORA,STAT1	4
IRF1 (includes EG:16362),RARRES3,STAT1	3
IRF1 (includes EG:16362),RARRES3,STAT1	3
IRF1 (includes EG:16362),RARRES3,STAT1	3
ITGA4,LCK,PI3,PKN2,SLPI	5
ITGA4,LCK,PI3,PKN2,SLPI	5
CD274,ITGA4,PECAM1,PF4,PPBP	5
CD274,ITGA4,PECAM1,PF4,PPBP	5
CD274,ITGA4,PECAM1,PF4,PPBP	5
CASP1,IRF1 (includes EG:16362),RARRES3,STAT1	4
CASP1,IRF1 (includes EG:16362),RARRES3,STAT1	4
CASP1,IRF1 (includes EG:16362),RARRES3,STAT1	4
BCL11B,CD274,CX3CR1,F2RL1,ITGA4,LCK,LTB,PECAM1,RASGRP1	9
BCL11B,CD274,CX3CR1,F2RL1,ITGA4,LCK,LTB,PECAM1,RASGRP1	9
BCL11B,CD274,CX3CR1,F2RL1,ITGA4,LCK,LTB,PECAM1,RASGRP1	9
CLEC7A,STAT1	2
CLEC7A,STAT1	2
CLEC7A,STAT1	2
IGHM,LTB	2
IGHM,LTB	2
IGHM,LTB	2
IGHM,LTB	2
IGHM,LTB	2
LCN2,PECAM1	2
IRF1 (includes EG:16362),OLFM4	2
IRF1 (includes EG:16362),LCK	2
PF4,PPBP	2
PF4,PPBP	2
PF4,PPBP	2
PF4,PPBP	2
CASP1,HSP90AB1,IRF1 (includes EG:16362),LCN2,PF4,PTGER4,SLPI,STAT1	8
CD274,EPSTI1,HCAR3,IFIT2,IFIT3,IGHM,PF4,PSMB9,TNS1	9
BCL11B,EPB42,HLA-DQB1,IGHM,IL7R,IRF1 (includes EG:16362),LCK,LCN2,PEC	10
BCL11B,EPB42,HLA-DQB1,IGHM,IL7R,IRF1 (includes EG:16362),LCK,LCN2,PEC	10
BCL11B,EPB42,HLA-DQB1,IGHM,IL7R,IRF1 (includes EG:16362),LCK,LCN2,PEC	10
BCL11B,CASP1,GIMAP4,GZMA,IGHM,LCN2,PF4	7
CD274,EPSTI1,HCAR3,IFIT2,IFIT3,PF4	6
CD274,EPSTI1,HCAR3,IFIT2,IFIT3,PF4	6
CD274,EPSTI1,HCAR3,IFIT2,IFIT3,PF4	6
CASP1,CLEC7A,DDX58,IRF1 (includes EG:16362),PTGER4,SLPI,STAT1	7
CASP1,CLEC7A,DDX58,IRF1 (includes EG:16362),PTGER4,SLPI,STAT1	7
ALOX12,CD46,FCGR3B,ITGA4,LCK,PECAM1,PF4,PPBP,PTGER4,RASGRP1	10
ALOX12,CD46,FCGR3B,ITGA4,LCK,PECAM1,PF4,PPBP,PTGER4,RASGRP1	10
ALOX12,CD46,FCGR3B,ITGA4,LCK,PECAM1,PF4,PPBP,PTGER4,RASGRP1	10
ALOX12,CD46,FCGR3B,ITGA4,LCK,PECAM1,PF4,PPBP,PTGER4,RASGRP1	10
F2RL1,IGHM,IRF1 (includes EG:16362),LTB,PI3	5
F2RL1,IGHM,IRF1 (includes EG:16362),LTB,PI3	5
F2RL1,IGHM,IRF1 (includes EG:16362),LTB,PI3	5
CASP1,CLEC7A,IRF1 (includes EG:16362),PTGER4,SLPI,STAT1	6
CASP1,CLEC7A,IRF1 (includes EG:16362),PTGER4,SLPI,STAT1	6
PF4,PPBP	2
PF4,PPBP	2

IGHM,IL7R,PECAM1,SEMA4D	4
IGHM,IL7R,PECAM1,SEMA4D	4
IGHM,IL7R,PECAM1,SEMA4D	4
IRF1 (includes EG:16362),LCN2,STAT1	3
BCL11B,EPB42,HLA-DQB1,IGHM,IL7R,IRF1 (includes EG:16362),LTB	7
BCL11B,EPB42,HLA-DQB1,IGHM,IL7R,IRF1 (includes EG:16362),LTB	7
BCL11B,EPB42,HLA-DQB1,IGHM,IL7R,IRF1 (includes EG:16362),LTB	7
BCL11B,EPB42,HLA-DQB1,IGHM,IL7R,IRF1 (includes EG:16362),LTB	7
BCL11B,EPB42,HLA-DQB1,IGHM,IL7R,IRF1 (includes EG:16362),LTB	7
BCL11B,DDX58,HLA-DQA1,HLA-DQB1,IGHM,IL7R,LCK,LTB,RASGRP1	9
BCL11B,DDX58,HLA-DQA1,HLA-DQB1,IGHM,IL7R,LCK,LTB,RASGRP1	9
CAMK1D,CASP1,F2RL1,ITGA4,PDE4B,PECAM1,PF4,PPBP,SLPI	9
CAMK1D,CASP1,F2RL1,ITGA4,PDE4B,PECAM1,PF4,PPBP,SLPI	9
CAMK1D,CASP1,F2RL1,ITGA4,PDE4B,PECAM1,PF4,PPBP,SLPI	9
CAMK1D,CASP1,F2RL1,ITGA4,PDE4B,PECAM1,PF4,PPBP,SLPI	9
IL7R,LTB,PECAM1,PPBP,STAT1	5
IRF1 (includes EG:16362),LCK,SGK1	3
CASP1,IRF1 (includes EG:16362),RARRES3,STAT1	4
CASP1,IRF1 (includes EG:16362),RARRES3,STAT1	4
CASP1,IRF1 (includes EG:16362),RARRES3,STAT1	4
CD274,F2RL1,HLA-DQB1,IGHM,LCK,LTB,RASGRP1,SEMA4D	8
CD274,F2RL1,HLA-DQB1,IGHM,LCK,LTB,RASGRP1,SEMA4D	8
BCL11B,DDX58,HLA-DQA1,HLA-DQB1,IGHM,IL7R,LCK,LTB,RASGRP1,STAT1	10
CASP1,FCGR3B,GZMA,LCK,STAT1,TAP2	6
ALOX12,CASP1,HSP90AB1	3
CAMK1D,CLEC7A,F2RL1,FCGR3B,IGHM,LY75,PECAM1,PF4,SNCA	9
DDX58,GPR146,IRF1 (includes EG:16362),RARRES3	4
CASP1,GBP1,GBP2 (includes EG:14468),HSP90AB1,IRF1 (includes EG:16362),L(	8
CASP1,CD274,CLEC7A,HLA-DQB1,IL7R,LTB,PF4	7
BCL11B,IL7R,LCK	3
BCL11B,IL7R,LCK	3
LRRK2,SNCA	2
LRRK2,SNCA	2
LRRK2,SNCA	2
PF4,PPBP	2
PF4,PPBP	2
PF4,PPBP	2
IGHM,IL32,IL7R,LEF1,PF4,RASGRP1,STAT1	7
IGHM,IL32,IL7R,LEF1,PF4,RASGRP1,STAT1	7
IGHM,IL32,IL7R,LEF1,PF4,RASGRP1,STAT1	7
IGHM,IL32,IL7R,LEF1,PF4,RASGRP1,STAT1	7
F2RL1,GZMA,LCN2,PF4,RORA,SLPI,SNCA,STAT1	8
F2RL1,GZMA,LCN2,PF4,RORA,SLPI,SNCA,STAT1	8
F2RL1,GZMA,LCN2,PF4,RORA,SLPI,SNCA,STAT1	8
F2RL1,GZMA,LCN2,PF4,RORA,SLPI,SNCA,STAT1	8
CD274,EPSTI1,HCAR3,IFIT2,IFIT3,PF4,PSMB9,TNS1	8
CD274,EPSTI1,HCAR3,IFIT2,IFIT3,PF4,PSMB9,TNS1	8
CD274,EPSTI1,HCAR3,IFIT2,IFIT3,PF4,PSMB9,TNS1	8
ALOX12,HSP90AB1,IRF1 (includes EG:16362),LCN2,PRKCH,STAT1	6
CD274,LY75,PSMB9	3
CD274,LY75,PSMB9	3
CD274,IRF1 (includes EG:16362),STAT1	3

CD274,IRF1 (includes EG:16362),STAT1	3
ANKRD9,CA2,CAMK1D,CARD16,CASP1,CD46,DDX58,DDX60L,IRF1 (includes EG	15
CAMK1D,CASP1,F2RL1,ITGA4,OLFM4,PDE4B,PECAM1,PF4,PPBP,SLPI	10
CAMK1D,CASP1,F2RL1,ITGA4,OLFM4,PDE4B,PECAM1,PF4,PPBP,SLPI	10
CAMK1D,CASP1,F2RL1,ITGA4,OLFM4,PDE4B,PECAM1,PF4,PPBP,SLPI	10
BCL11B,CD274,F2RL1,ITGA4,LCK,LTB,RASGRP1	7
BCL11B,CD274,F2RL1,ITGA4,LCK,LTB,RASGRP1	7
BCL11B,CD274,F2RL1,ITGA4,LCK,LTB,RASGRP1	7
BCL11B,CD274,F2RL1,ITGA4,LCK,LTB,RASGRP1	7
HLA-DQB1,IL7R,ITGA4,PSMB9,RORA	5
HLA-DQB1,IL7R,ITGA4,PSMB9,RORA	5
HLA-DQB1,IL7R,ITGA4,PSMB9,RORA	5
CD274,IRF1 (includes EG:16362),LEF1,PTGER4,RORA,STAT1	6
CD274,IRF1 (includes EG:16362),LEF1,PTGER4,RORA,STAT1	6
CD274,IRF1 (includes EG:16362),LEF1,PTGER4,RORA,STAT1	6
CD274,IRF1 (includes EG:16362),LEF1,PTGER4,RORA,STAT1	6
CD274,IRF1 (includes EG:16362),LEF1,PTGER4,RORA,STAT1	6
CD274,IRF1 (includes EG:16362),LEF1,PTGER4,RORA,STAT1	6
CD274,ITGA4,PECAM1,PPBP,SLPI	5
CD274,ITGA4,PECAM1,PPBP,SLPI	5
CD274,ITGA4,PECAM1,PPBP,SLPI	5
GZMA,IL7R,LCN2	3
CD274,HLA-DQB1,IGHM,LCK,LTB	5
CD274,HLA-DQB1,IGHM,LCK,LTB	5
DDX58,HLA-DQA1,HLA-DQB1,IGHM,IL7R,LCK,LTB,RASGRP1	8
DDX58,HLA-DQA1,HLA-DQB1,IGHM,IL7R,LCK,LTB,RASGRP1	8
CA2,CARD16,F2RL1,FGL2,GZMA,HSP90AB1,LCN2,LTB,OLFM4,PI3,SEMA4D,SL	15
CASP1,IRF1 (includes EG:16362),LCK,LEF1,MX1,STAT1	6
AK1,CD274,GAS2L1,IGHM,LCK,LEF1,PI3,PRKCH,STAT1	9
BAG1,CAMK1D,DDX58,DDX60L,HERC5,IRF1 (includes EG:16362),LEF1,MDFIC,M	12
LCK,PECAM1,PTGER4	3
LCK,PECAM1,PTGER4	3
LCK,PECAM1,PTGER4	3
LCK,PECAM1,PTGER4	3
LCK,PECAM1,PTGER4	3
DDX58,LCK,RARRES3	3
CD274,HLA-DQA1,IGHM,IL7R,LCN2,PECAM1,RASGRP1,SEMA4D	8
CD274,HLA-DQA1,IGHM,IL7R,LCN2,PECAM1,RASGRP1,SEMA4D	8
CD274,HLA-DQA1,IGHM,IL7R,LCN2,PECAM1,RASGRP1,SEMA4D	8
CX3CR1,HLA-DQA1	2
ALOX12,LCN2	2
ALOX12,LCN2	2
ALOX12,CASP1,GIMAP4,LEF1	4
GBP1,GBP2 (includes EG:14468),HSP90AB1,IRF1 (includes EG:16362),LCK,PEC	9
GBP1,GBP2 (includes EG:14468),HSP90AB1,IRF1 (includes EG:16362),LCK,PEC	9
BCL11B,CD274,DDX58,EPB42,HLA-DQA1,HLA-DQB1,IGHM,IRF1 (includes EG:1	15
BCL11B,F2RL1,ITGA4,LTB	4
BCL11B,F2RL1,ITGA4,LTB	4
BCL11B,F2RL1,ITGA4,LTB	4
BCL11B,F2RL1,ITGA4,LTB	4
HSP90AB1,IGHM,LY75,UBE2L6	4
HSP90AB1,IGHM,LY75,UBE2L6	4

IGHM,LTB,SLPI,STAT1	4
HLA-DQB1,IGHM	2
HLA-DQB1,IGHM	2
HSP90AB1,STAT1	2
CD274,GIMAP4	2
CD274,IGHM,IL7R,LCN2,LTB,PECAM1,ZFP36L2	7
CD274,IGHM,IL7R,LCN2,LTB,PECAM1,ZFP36L2	7
IGHM,IL7R,RASGRP1,TAP2	4
CAMK1D,CLEC7A,F2RL1,FCGR3B,LY75,PECAM1,PF4	7
CAMK1D,CLEC7A,F2RL1,FCGR3B,LY75,PECAM1,PF4	7
CAMK1D,CLEC7A,F2RL1,FCGR3B,LY75,PECAM1,PF4	7
CASP1,CD274,CLEC7A,HLA-DQB1,IL7R,IRF1 (includes EG:16362)	6
CASP1,IRF1 (includes EG:16362),STAT1	3
BCL11B,CD52,IL7R,IRF1 (includes EG:16362),ITGA4,LCK,POLB	7
CD274,IRF1 (includes EG:16362),LEF1,STAT1	4
CD274,IRF1 (includes EG:16362),LEF1,STAT1	4
CD274,IRF1 (includes EG:16362),LEF1,STAT1	4
CD274,IRF1 (includes EG:16362),LEF1,STAT1	4
CD274,IRF1 (includes EG:16362),LEF1,STAT1	4
CD274,IRF1 (includes EG:16362),LEF1,STAT1	4
CD274,GZMA,MME,PECAM1	4
CD274,GZMA,MME,PECAM1	4
CD274,GZMA,MME,PECAM1	4
CD274,GZMA,MME,PECAM1	4
DDX58,IRF1 (includes EG:16362),STAT1	3
DDX58,IRF1 (includes EG:16362),STAT1	3
GBP1,GBP2 (includes EG:14468),HSP90AB1,IRF1 (includes EG:16362),LCK,PEC/	7
GBP1,GBP2 (includes EG:14468),HSP90AB1,IRF1 (includes EG:16362),LCK,PEC/	7
CX3CR1,LCK	2
CX3CR1,LCK	2
CX3CR1,LCK	2
CX3CR1,LCK	2
CX3CR1,LCK	2
CX3CR1,LCK	2
IRF1 (includes EG:16362),KCNJ2	2
CX3CR1,LRRK2,SEMA4D	3
CX3CR1,LRRK2,SEMA4D	3
CX3CR1,LRRK2,SEMA4D	3
CX3CR1,LRRK2,SEMA4D	3
BCL11B,IL7R,POLB	3
BCL11B,IL7R,POLB	3
BCL11B,IL7R,POLB	3
HLA-DQB1,IGHM,IL7R,LTB	4
HLA-DQB1,IGHM,IL7R,LTB	4
HLA-DQB1,IGHM,IL7R,LTB	4
HLA-DQB1,IGHM,IL7R,LTB	4
HLA-DQB1,IGHM,IL7R,LTB	4
ITGA4,PECAM1,PPBP,SLPI	4
ITGA4,PECAM1,PPBP,SLPI	4
ITGA4,PECAM1,PPBP,SLPI	4
ITGA4,PECAM1,PPBP,SLPI	4
ALOX12,BAG1,HSPB1,POLB,SEMA4D,SNCA	6

CD274,F2RL1,HLA-DQB1,IGHM,LCK,LTB,RASGRP1	7
CD274,F2RL1,HLA-DQB1,IGHM,LCK,LTB,RASGRP1	7
ALOX12,HSPB1,IRF1 (includes EG:16362),LCN2	4
ALOX12,HSPB1,IRF1 (includes EG:16362),LCN2	4
ALOX12,HSPB1,IRF1 (includes EG:16362),LCN2	4
CX3CR1,F2RL1,IGHM,IRF1 (includes EG:16362),LCN2,LTB,PDE4B,PI3,ZFP36L2	9
CX3CR1,F2RL1,IGHM,IRF1 (includes EG:16362),LCN2,LTB,PDE4B,PI3,ZFP36L2	9
CX3CR1,F2RL1,IGHM,IRF1 (includes EG:16362),LCN2,LTB,PDE4B,PI3,ZFP36L2	9
CD274,SLAMF7,STAT1	3
CASP1,CX3CR1,F2RL1,SNCA,STAT1	5
CASP1,CX3CR1,F2RL1,SNCA,STAT1	5
IL7R,STAT1	2
IL7R,STAT1	2
F2RL1,IL32	2
F2RL1,IL32	2
F2RL1,IL32	2
HLA-DQB1,IGHM,LTB	3
DDX58,IL7R,LTB	3
DDX58,IL7R,LTB	3
DDX58,IL7R,LTB	3
DDX58,IL7R,LTB	3
ITGA4,PECAM1,PPBP	3
ITGA4,PECAM1,PPBP	3
CASP1,EPB42,ITGA4,LRRK2,MME,PDE4B,SNCA	7
BCL11B,HLA-DQB1,IL7R,IRF1 (includes EG:16362),LCK,RASGRP1	6
BCL11B,HLA-DQB1,IL7R,IRF1 (includes EG:16362),LCK,RASGRP1	6
BCL11B,HLA-DQB1,IL7R,IRF1 (includes EG:16362),LCK,RASGRP1	6
CD274,IL7R,RASGRP1	3
CD274,IL7R,RASGRP1	3
CD274,IL7R,RASGRP1	3
CD274,IGHM,LCK,LTB	4
CD274,IGHM,LCK,LTB	4
CD46,IRF1 (includes EG:16362),PI3,POLB,STAT1	5
BCL11B,IGHM,IL7R,LCK	4
BCL11B,IGHM,IL7R,LCK	4
BCL11B,ITGA4,LEF1	3
BCL11B,ITGA4,LEF1	3
LCK,PPBP,SEMA4D	3
LCK,PPBP,SEMA4D	3
LCK,PPBP,SEMA4D	3
BCL11B,IGHM,IL7R,IRF1 (includes EG:16362),PECAM1	5
BCL11B,IGHM,IL7R,IRF1 (includes EG:16362),PECAM1	5
BCL11B,IGHM,IL7R,IRF1 (includes EG:16362),PECAM1	5
CASP1,CX3CR1,LRRK2,LTB,PF4,RETN,SEMA4D	7
CASP1,CX3CR1,LRRK2,LTB,PF4,RETN,SEMA4D	7
CASP1,CX3CR1,LRRK2,LTB,PF4,RETN,SEMA4D	7
CASP1,CX3CR1,LRRK2,LTB,PF4,RETN,SEMA4D	7
BCL11B,CASP1,GIMAP4,GZMA,LCN2	5
ITGA4,LY75,RORA,SELENBP1,SLPI	5
ITGA4,LY75,RORA,SELENBP1,SLPI	5
FCGR3B,GZMA,LCK,STAT1,TAP2	5
IGHM,IL7R,ITGA4,RORA	4

IGHM,IL7R,ITGA4,RORA	4
IGHM,IL7R,ITGA4,RORA	4
IGHM,IL7R,ITGA4,RORA	4
IGHM,IL7R,ITGA4,RORA	4
BCL11B,F2RL1,ITGA4,LTB,PF4	5
BCL11B,F2RL1,ITGA4,LTB,PF4	5
BCL11B,F2RL1,ITGA4,LTB,PF4	5
IGHM,LCK	2
IGHM,LCK	2
IL7R,LCK	2
IL7R,LCK	2
IL7R,LCK	2
IL7R,LCK	2
IL7R,LCK	2
IL7R,LCK	2
F2RL1,SNCA	2
F2RL1,SNCA	2
F2RL1,SNCA	2
DDX58,RARRES3	2
IRF1 (includes EG:16362),LCN2,PRKCH,STAT1	4
CASP1,F2RL1,GZMA,STAT1	4
F2RL1,HEMGN,IFIT2,MS4A4A	4
IRF1 (includes EG:16362),PPBP,STAT1,TAP2	4
IRF1 (includes EG:16362),PPBP,STAT1,TAP2	4
IRF1 (includes EG:16362),PPBP,STAT1,TAP2	4
ANKRD9,CA2,CAMK1D,CARD16,CD46,DDX58,DDX60L,LCN2,PKN2,STAT1	10
ANKRD9,CA2,CAMK1D,CARD16,CD46,DDX58,DDX60L,LCN2,PKN2,STAT1	10
CASP1,HSP90AB1,PF4,PTGER4	4
BCL11B,IL7R,IRF1 (includes EG:16362),LCK	4
BCL11B,IL7R,IRF1 (includes EG:16362),LCK	4
BCL11B,IL7R,IRF1 (includes EG:16362),LCK	4
AK1,BCL11B,BTN3A1,CA2,CASP1,GBP1,MAN1A1,OSBPL8,PSMB9,RARRES3,S[illegible]	11
AK1,BCL11B,BTN3A1,CA2,CASP1,GBP1,MAN1A1,OSBPL8,PSMB9,RARRES3,S[illegible]	11
AK1,BCL11B,BTN3A1,CA2,CASP1,GBP1,MAN1A1,OSBPL8,PSMB9,RARRES3,S[illegible]	11
HLA-DQB1,ITGA4,PSMB9,RORA	4
HLA-DQB1,ITGA4,PSMB9,RORA	4
HLA-DQB1,ITGA4,PSMB9,RORA	4
AK1,BCL11B,BTN3A1,CA2,CASP1,GBP1,MAN1A1,MME,OSBPL8,PSMB9,RARR[illegible]	12
F2RL1,FCGR3B,PECAM1,PF4,PILRA,PPBP	6
F2RL1,FCGR3B,PECAM1,PF4,PILRA,PPBP	6
LCK,PECAM1	2
LCK,PECAM1	2
LCK,PECAM1	2
LCK,PECAM1	2
LCK,PECAM1	2
IL7R,LTB	2
IL7R,LTB	2
IL7R,LTB	2
IL7R,LTB	2
CASP1,HLA-DQB1,IGHM,LTB,RASGRP1	5
GBP1,GBP2 (includes EG:14468),HSP90AB1,IRF1 (includes EG:16362),LCK,PEC[illegible]	8
GBP1,GBP2 (includes EG:14468),HSP90AB1,IRF1 (includes EG:16362),LCK,PEC[illegible]	8

ITGA4,LCK,PPBP,SEMA4D	4
ITGA4,LCK,PPBP,SEMA4D	4
ITGA4,LCK,PPBP,SEMA4D	4
ALOX12,BAG1,CASP1,DDX58,HSP90AB1,HSPB1,IGHM,IL32,IRF1 (includes EG:1	20
F2RL1,LCN2,PF4,SLPI	4
F2RL1,LCN2,PF4,SLPI	4
F2RL1,LCN2,PF4,SLPI	4
F2RL1,LCN2,PF4,SLPI	4
CA2,CASP1,CLEC7A,F2RL1,FCGR3B,IGHM,PECAM1,PPBP,PRKCH,PTGER4,SC	11
CLEC7A,F2RL1,IRF1 (includes EG:16362),RARRES3,STAT1	5
CLEC7A,F2RL1,IRF1 (includes EG:16362),RARRES3,STAT1	5
IGHM,PECAM1	2
IGHM,PECAM1	2
IGHM,PECAM1	2
DDX58,IRF1 (includes EG:16362)	2
CASP1,CD274,HLA-DQB1,IL7R	4
CASP1,CD274,HLA-DQB1,IL7R	4
ALOX12,BAG1,CASP1,DDX58,HSP90AB1,HSPB1,IGHM,IL32,IRF1 (includes EG:1	23
AK1,BCL11B,BTN3A1,CA2,CASP1,GBP1,HSPB1,LRRK2,MAN1A1,OSBPL8,PDE4	16
F2RL1,PECAM1,PTGER4	3
F2RL1,PECAM1,PTGER4	3
BCL11B,CASP1,CD274,HLA-DQA1,HLA-DQB1,SELENBP1	6
CASP1,IRF1 (includes EG:16362)	2
CASP1,IRF1 (includes EG:16362)	2
PF4,PPBP	2
PF4,PPBP	2
PF4,PPBP	2
PF4,PPBP	2
PF4,PPBP	2
F2RL1,SNCA	2
KCNJ2	1
KCNJ2	1
KCNJ2	1
LCN2	1
LCN2	1
F2RL1,HLA-DQB1,IGHM,ITGA4,LRRK2,PDE4B	6
F2RL1,HLA-DQB1,IGHM,ITGA4,LRRK2,PDE4B	6
F2RL1,HLA-DQB1,IGHM,ITGA4,LRRK2,PDE4B	6
F2RL1,HLA-DQB1,IGHM,ITGA4,LRRK2,PDE4B	6
F2RL1,HLA-DQB1,IGHM,ITGA4,LRRK2,PDE4B	6
CASP1,CD274,LTB,PDE4B,PF4,POLB	6
CASP1,CD274,LTB,PDE4B,PF4,POLB	6
CASP1,CD274,LTB,PDE4B,PF4,POLB	6
CASP1,CD274,LTB,PDE4B,PF4,POLB	6
LTB	1
LTB	1
LTB	1
LCN2	1
LCN2	1
LCN2	1
LCN2	1
CASP1,LRRK2	2

CASP1,LRRK2	2
PDE4B,PF4	2
PDE4B,PF4	2
PDE4B,PF4	2
PDE4B,PF4	2
KCNJ2	1
KCNJ2	1
KCNJ2	1
BAG1,CASP1,HSPB1,LCN2,STAT1	5
BAG1,CASP1,HSPB1,LCN2,STAT1	5
CD274,POLB	2
CD274,POLB	2
CD274,POLB	2
CD274,POLB	2
CD274,POLB	2
PECAM1	1
PECAM1	1
ITGA4	1
ITGA4	1
ITGA4	1
HLA-DQB1	1
HLA-DQB1	1
HLA-DQB1	1
HLA-DQB1	1
HLA-DQB1	1
LCN2	1
LCN2	1
LCN2	1
LCN2	1
IGHM	1
IGHM	1
IGHM	1
IGHM	1
IGHM	1
BAG1,CASP1,LCN2,STAT1	4
BAG1,CASP1,LCN2,STAT1	4
CASP1,TNS1	2
CASP1,TNS1	2
CASP1,TNS1	2
LTB,PSMB9	2
LTB,PSMB9	2
LTB,PSMB9	2
LTB,PSMB9	2
LTB,PSMB9	2
CD274,LTB,POLB	3
CD274,LTB,POLB	3
CD274,LTB,POLB	3
CD274,LTB,POLB	3
F2RL1,STAT1	2
F2RL1,STAT1	2
F2RL1,STAT1	2
F2RL1,STAT1	2

F2RL1,STAT1	2
F2RL1,STAT1	2
LCN2	1
LCN2	1
